# Variations in biocorona formation related to defects in the structure of single walled carbon nanotubes and the hyperlipidemic disease state

**DOI:** 10.1038/s41598-017-08896-w

**Published:** 2017-08-16

**Authors:** Achyut J. Raghavendra, Kristofer Fritz, Sherleen Fu, Jared M. Brown, Ramakrishna Podila, Jonathan H. Shannahan

**Affiliations:** 1 Department of Physics and Astronomy, Clemson University, Clemson, South Carolina 29634 USA; 2 Clemson Nanomaterials Center and COMSET, Clemson University, Anderson, South Carolina 29625 USA; 3 Department of Pharmaceutical Sciences, Skaggs School of Pharmacy and Pharmaceutical Sciences, The University of Colorado Anschutz Medical Campus, Aurora, Colorado 80045 USA; 4 School of Health Sciences, College of Human and Health Sciences, Purdue University, West Lafayette, IN 47907 USA; 5 Colorado Center for Nanomedicine and Nanosafety, Skaggs School of Pharmacy and Pharmaceutical Sciences, The University of Colorado Anschutz Medical Campus, Aurora, Colorado 80045 USA

## Abstract

Ball-milling utilizes mechanical stress to modify properties of carbon nanotubes (CNTs) including size, capping, and functionalization. Ball-milling, however, may introduce structural defects resulting in altered CNT-biomolecule interactions. Nanomaterial-biomolecule interactions result in the formation of the biocorona (BC), which alters nanomaterial properties, function, and biological responses. The formation of the BC is governed by the nanomaterial physicochemical properties and the physiological environment. Underlying disease states such as cardiovascular disease can alter the biological milieu possibly leading to unique BC identities. In this *ex vivo* study, we evaluated variations in the formation of the BC on single-walled CNTs (SWCNTs) due to physicochemical alterations in structure resulting from ball-milling and variations in the environment due to the high-cholesterol disease state. Increased ball-milling time of SWCNTs resulted in enhanced structural defects. Following incubation in normal mouse serum, label-free quantitative proteomics identified differences in the biomolecular content of the BC due to the ball-milling process. Further, incubation in cholesterol-rich mouse serum resulted in the formation of unique BCs compared to SWCNTs incubated in normal serum. Our study demonstrates that the BC is modified due to physicochemical modifications such as defects induced by ball-milling and physiological disease conditions, which may result in variable biological responses.

## Introduction

Single walled carbon nanotubes (SWCNTs) are one-dimensional structures with unique optical and electronic properties relevant for many biomedical applications^1–3^. Particularly, the sharp densities of electronic states at the so-called van Hove singularities (vHS) in SWCNTs impart strong resonant optical absorption and emission of visible and near-infrared light, which makes them invaluable for applications in photothermal therapy, multimodal imaging (e.g., Raman, fluorescence and photoacoustic), and cancer drug delivery^4, 5^. While much research has focused on exploiting SWCNT properties for biomedical applications, fundamental understanding of SWCNT biological interactions and mechanisms of toxicity still remain elusive^6–8^.

In physiological environments, SWCNTs interact with cells through the biocorona (BC), which consists of a layer of inadvertently physi- and chemisorbed biomolecules (viz., proteins, lipids, peptides, etc.) on the surface of the SWCNTs^9–12^. The addition of the BC alters not only the surface and properties of the SWCNTs but may also modify their cellular interactions similar to what has been shown with other nanoparticles^13–17^. Ultimately, this means that the BC can interfere with the intended biological applications of SWCNTs (viz., imaging or drug delivery) by altering their biodistribution, clearance, and/or toxicity. Specifically, research has demonstrated that the targeting benefits of functionalizing nanoparticles with transferrin for specific interactions with transferrin receptors is lost due to the addition of the BC^18^. In general, it has been demonstrated that the physicochemical properties of nanomaterials (size, surface coatings, zeta potential, etc.) influence the formation and the content of BC, however, the impact of structural defects on the BC has not been fully evaluated^19–22^.

The formation of the BC on SWCNTs is fundamentally intriguing due to the presence of vHS in their electronic structure. Previously, we showed that the vHS in SWCNTs participated in charge-transfer interactions with proteins such as fibrinogen and thereby elicited undesired thrombosis^23^. The electronic structure of SWCNTs is highly sensitive to defects, which are often unintentionally introduced in SWCNTs while processing them through mechanical or chemical functionalization for biological applications^24, 25^. We hypothesize that the presence of defects alters SWCNT biomolecular interactions through charge-transfer interactions and could ultimately change the composition of BC. Understanding BC compositional differences due to defects in SWCNTs will allow for new avenues of control regarding nanoparticle-biomolecule interactions. This control is needed to mitigate toxicity as well as utilize the BC in therapeutic and diagnostic applications.

In addition to the defects in the SWCNT structure, the composition of physiological environment also has a significant impact on the formation of the BC with implications in SWCNT-biomolecular interactions and subsequent cellular responses. In individuals suffering from underlying diseases (e.g., cardiovascular diseases such as high cholesterol), which modify serum biomolecule content, SWCNTs and other nanomaterials are likely to form unique BCs as compared to healthy individuals. Individuals suffering from high cholesterol constitute a prominent and growing subpopulation in our society. Understanding BC formation in this subpopulation is necessary for the safe and effective use of nanoparticles in biomedical applications. For example, our experiments utilizing Fe_3_O_4_ nanoparticles (NPs) have demonstrated that unique BCs form following incubation in high-cholesterol serum compared to the BCs formed in normal serum^26^. This unique BC on Fe_3_O_4_ NPs that formed in high cholesterol serum exacerbated the inflammatory response of endothelial cells following exposure, when compared to Fe_3_O_4_ NPs with a normal serum BC^26^. This finding demonstrates that disease-induced alterations in the physiological environment can impact NP biological response by altering the BC. Thereby, based on these observations, it is imperative to evaluate differences in the BC that forms under these increasingly prominent disease states for a comprehensive assessment of nanotoxicity.

In the current evaluation of the BC we hypothesized that the defects in SWCNTs will result in differential association of biomolecules forming the BC. Additionally, we hypothesized that disease-associated differences in the physiological media would also alter BC formation. To examine the role of defects and a high cholesterol environment on the formation of the SWCNT-BC, we utilized a quantitative proteomics approach with label-free mass spectrometry. We prepared SWCNTs with different defects using a planetary ball-milling approach. The defects in SWCNTs were characterized using a comprehensive array of tools including transmission electron microscopy, Raman and photoluminescence spectroscopy, and gas-adsorption isotherms. We observed that increased ball-milling time of SWCNTs resulted in more defects introduced in the structure of SWCNTs. Following incubation in normal mouse serum, quantitative label-free mass spectrometry identified differences in the biomolecular content of the BC resulting from the ball-milling process. Further, incubation in cholesterol-rich mouse serum resulted in the formation of unique BCs compared to SWCNTs incubated in normal serum. Ultimately our study demonstrates that the BC is modified due to physicochemical modifications in SWCNT structure induced by ball-milling and due to physiological disease conditions. These alterations in the BC may result in variable biological responses.

## Results and Discussion

### Description of Single-Walled Carbon Nanotubes following Ball Milling

**Figure 1:**
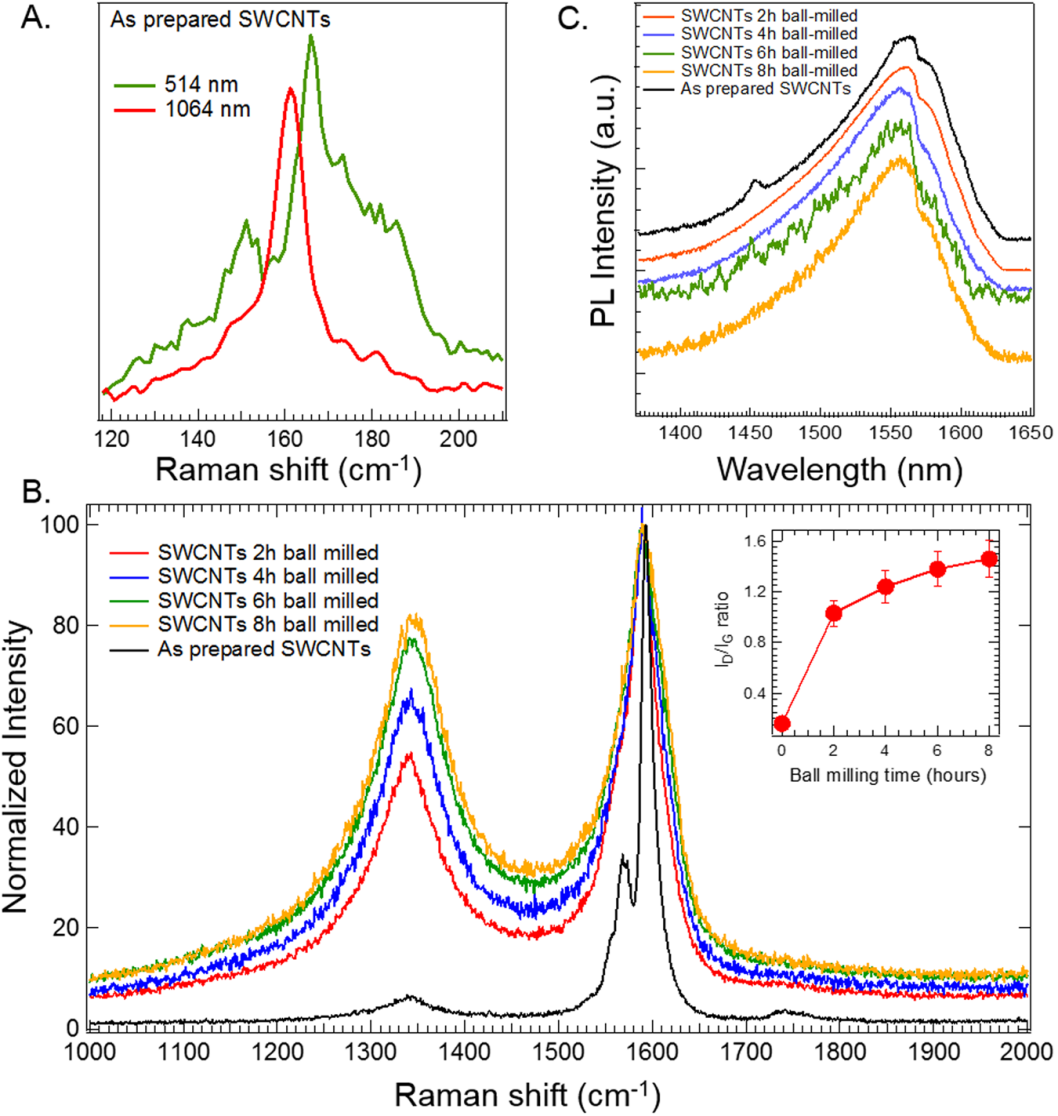
(**A**) Radial breathing modes from the Raman spectra of As Prepared SWCNTs suggest a diameter distribution ~1.4 nm. The spectra were collected using both 514 and 1064 nm to probe all SWCNT populations. (**B**) Alterations in the defect band (D-band ~1350 cm-1) in ball milled SWCNTs evidenced by their Raman spectra. All the spectra were normalized to the graphitic or G-band to obtain the ID/IG ratio that is indicative of the defect density. Also plot of ID/IG vs. duration of ball milling (inset) shows that there is an initial rapid increase for 2 h ball milling followed by a saturation in the defects with increasing ball milling time. The spectra shown in (**A**) and (**B**) are averaged over at least three different sets. (**C**) The photoluminescence spectra of As Prepared and ball milled SWCNTs showed an emission peak ~1560 nm. The presence of strong emission, unique to the tubular structure of SWCNTs, confirms that the defects were introduced while still retaining the tubular morphology. All characterization experiments were performed with an n ≥ 3.

SWCNTs were generated using a previously described chemical vapor deposition process^27^. The Raman spectrum of SWCNTs displays a unique radial breathing mode (RBM), which could be used to accurately determine the diameter distribution. As shown in Fig. 1A, the resonant Raman spectra of as-prepared SWCNTs at two excitation wavelengths (514 and 1064 nm) showed RBMs between 150–180 cm^−1^ indicating a sharp diameter distribution ~1.3–1.6 nm in agreement with transmission electron microscope (TEM) images (Supplemental Fig. 1). Ball milling is expected to induce structural defects by opening SWCNT end caps in addition to creating atomic vacancies and non-hexagonal rings in SWCNTs. Indeed, it has been previously shown that intensive ball milling for ~50 h completely disrupts SWCNT tubular structure leading to multi-layered polyaromatic carbon materials^28^. Accordingly, the ball milling time in this study was limited to ~8 h to induce defects while retaining the tubular structure of SWCNTs. The Raman spectrum of SWCNTs also was used to quantify the defect density based on the ratio of the disorder (or D-band) to graphitic band (or G-band) intensity (Fig. 1B). From Fig. 1B and C, it is evident that the D-to-G band intensity ratio (I_D_/I_G_) increased with increasing ball milling time from 0.16 in as-prepared SWCNTs to ~1.5 in 8 h ball milled samples. While SWCNTs ball milled for 2 h showed a rapid increase in I_D_/I_G_ ratio, higher ball milling times (4, 6, and 8 h) led to monotonic increase that appeared to be saturated by ~8 h (Fig. 1B: I_D_/I_G_ Ratio). All ball milled SWCNTs were found to retain their tubular structure, as evidenced by the photoluminescence (PL) spectra and TEM (Fig. 1C and Supplemental Fig. 1). The presence of the van Hove singularities (vHS) in SWCNT-electronic density of states results in strong PL emission in the infrared region^29^. As shown in Fig. 1C, a strong PL emission was observed for all SWCNTs ~1560 nm when excited at 1064 nm. This observation suggests that the one-dimensional tubular structure of SWCNTs, necessary for the presence of vHS, was retained in all the ball milled samples. The ball milling process is known to increase the specific surface area of SWCNTs through structural defects. To ascertain such changes in surface area, N_2_ gas adsorption experiments were performed. The surface area was found to have a montonic increase with ball milling time up to 6 h (Table 1). Indeed, the surface area for 6 h ball milled SWCNTs was found to increase by almost an order of magnitude. The significant decrease in surface area of 8 h-ball milled SWCNTs could be attributed to the formation of amorphous carbon that gets compacted during the milling process.

**Table 1:** Alterations in surface area of SWCNTs due to ball milling measured by BET analysis.

SWCNTs	Surface Area
As Prepared	169.668 m^2^/g
2 h ball milled	301.728 m^2^/g
4 h ball milled	351.015 m^2^/g
6 h ball milled	1091.402 m^2^/g
8 h ball milled	118.165 m^2^/g

### Proteomic Assessment of Variations in Biocorona Formation on SWCNTs due to Ball Milling and Differences in the Physiological Environment

Serum from mice fed with a normal or high cholesterol western diet were collected to evaluate the BCs that formed on SWCNTs. Specifically, following incubation in either 10% normal or hyperlipidemic serum, individual biocoronal proteins on SWCNTs were identified utilizing label-free mass spectrometry. This approach allowed for the identification of specific proteins included in the BCs of SWCNTs following incubation in either normal (Supplemental Table 1) or hyperlipidemic (Supplemental Table 2) serum. Following identification and assessment of relative quantitative differences between protein components of the BC, we evaluated alterations in the BC due to 1) ball milling (As Prepared vs Ball milled in normal or hyperlipidemic serum), and 2) physiological environment (normal vs hyperlipidemic serum). These comparisons were determined based on the common processing of SWCNTs by ball milling for manufacturing and the significant portion of our population that exist with hyperlipidemia. Ultimately, these comparisons describe differential biological interactions that may occur due to typical alterations in SWCNT structure and common disease-related environments.

### Proteomic Evaluation of the Variations in Biocorona Formation on SWCNTs due to Ball Milling

**Figure 2:**
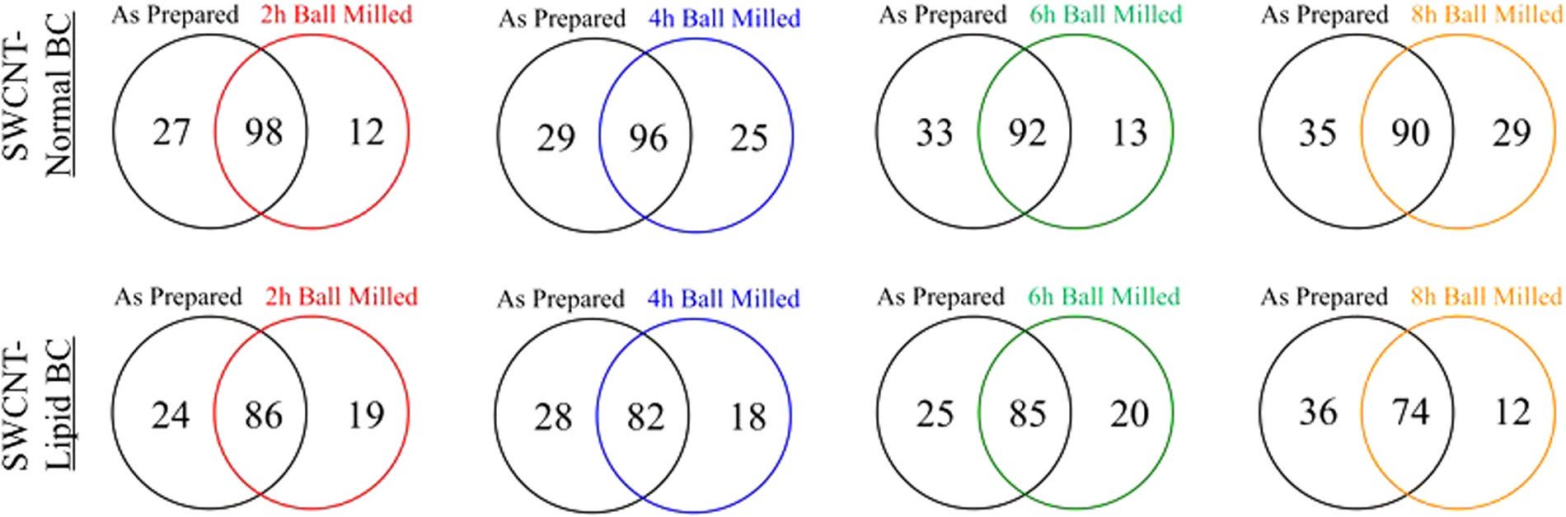
Venn diagrams representing the number of proteins found to associate with SWCNTs following incubation in either 10% normal or 10% hyperlipidemic serum comparing As Prepared SWCNTs to SWCNTs that have undergone ball milling (n = 4/group).

We hypothesized that the presence of structural defects in SWCNTs will alter biomolecule interactions. To evaluate these ball-milled induced changes, we compared the protein components of the BC following incubation in either normal or hyperlipidemic serum. Comparisons between the proteins of the BC that formed in normal and hyperlipidemic serum are included in the subsequent section. The identity of the majority of proteins forming the normal and lipid BC were found to be unchanged due to ball milling (Fig. 2, Supplemental Tables 3 and 4). These commonly identified proteins that did not depend upon the defect content included alpha-2-HS-glycoprotein, complement C3, fibrinogen, fibronectin, plasminogen, serum albumin and others in the normal BC (Supplemental Table 3) and alpha-2-HS-glycoprotein, complement C3, fibrinogen, fibronectin, serum albumin and others in the lipid BC (Supplemental Table 4). These proteins are similar to our previous findings of the proteins of the BC that form on multi-walled carbon nanotubes of differing characteristics and also metal- and metal-oxide nanoparticles (e.g., Ag and Fe_2_O_3_)^12, 26, 30^. These shared proteins are likely associated with the engineered nanoparticles due to their relatively high abundance in serum. Although many proteins found to associate with SWCNTs were common between as-prepared and defective ball milled SWCNTs, there were a number of unique protein components within each BC. Specifically, defective SWCNTs in general resulted in the association of platelet like factor 4, serum amyloid, carboxylesterase 1 C, metalloproteinase inhibitor and others during incubation in normal serum. The presence of defects in SWCNTs (as indicated by the I_D_/I_G_ ratio in Fig. 1C) also resulted in the unique association of apolipoprotein B-100, platelet like factor 4, glutathione peroxidase, phospholipid transfer protein, alpha-2 antiplasmin and others during incubation in hyperlipidemic serum. Interestingly, many proteins unique to the BC of ball milled SWCNTs, in both normal and hyperlipidemic serum, are known to function through redox and charge transfer mechanisms. For instance, glutathione peroxidase converts H_2_O_2_ into water through charge transfer reaction while metalloproteinase inhibitors chelate metal ions^31, 32^. Upon ball milling, the defects created in SWCNTs are often charged with exoelectrons and the Ni catalyst used in SWCNTs synthesis may be exposed unlike as-prepared SWCNTs^33^. It is plausible that the charged defects and exposed metal catalyst increased the affinity for unique proteins observed in the BC.

Further, for samples incubated in normal serum, fewer proteins were found to be in common between As-Prepared and ball milled SWCNTs with higher I_D_/I_G_ ratio (Figs 1C and 2). This trend was also evident in the SWCNTs incubated in hyperlipidemic serum with the exception of the 6 h ball milled group (Fig. 2). This suggests that a more diverse BC may form on the surface of SWCNTs due to the presence of defects. While the majority of proteins identified were found to be in common between As Prepared and defective ball milled SWCNTs, it is likely that these minor changes in protein identity may influence biological responses as we have previously demonstrated with silver nanoparticles. For example, serum albumin is the primary component of the BC that forms on silver nanoparticles due to its abundance^30^. However, when the inflammatory response of macrophages was evaluated following exposure to either silver nanoparticles with a BC that only consisted of albumin or a complex BC, differences were evident^34^. This suggests that even minor changes in the identity of the BC can elicit significant changes in biological responses.

**Figure 3:**
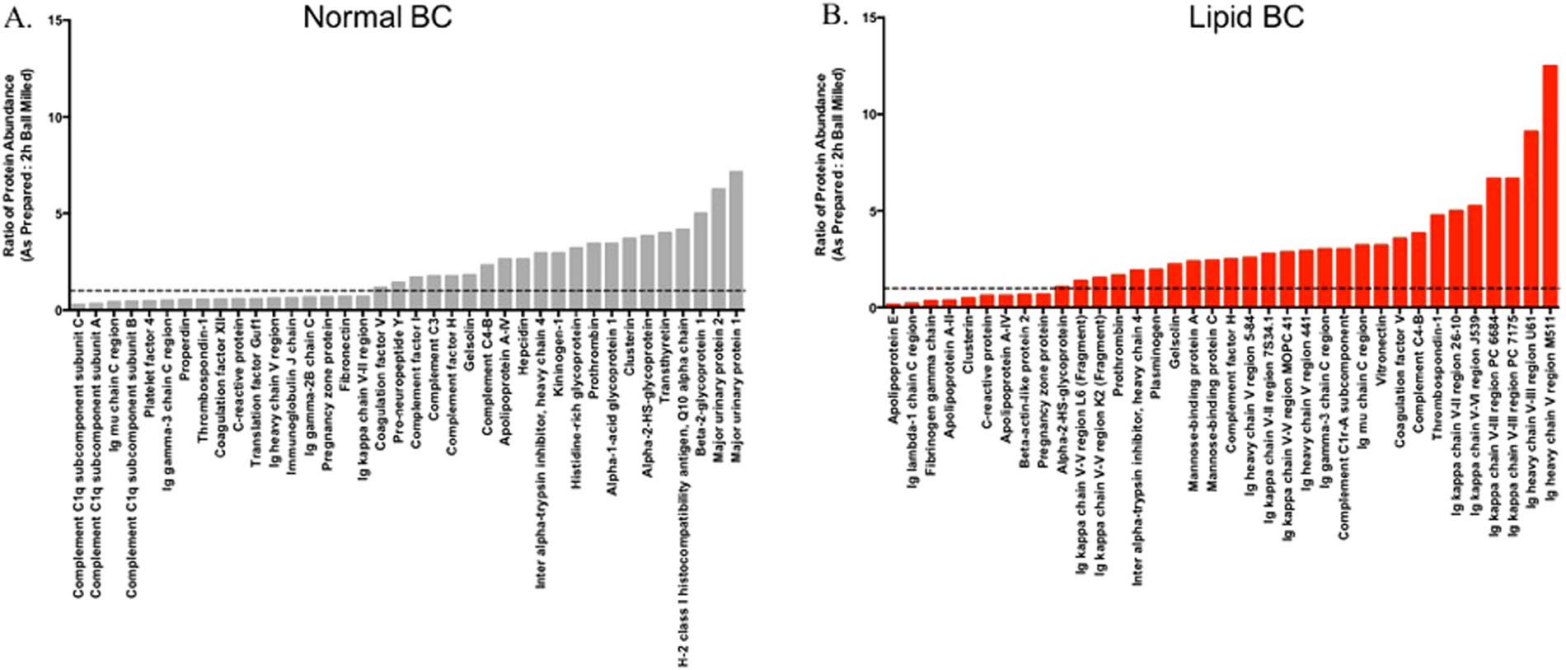
Relative abundance of proteins found to associate with 2 h ball milled SWCNTs compared to As Prepared SWCNTs in either normal (**A**) or hyperlipidemic (**B**) serum (n = 4/group). Additional data for other ball milled SWCNT samples can be found in Table S5 (normal serum) and Table S6 (hyperlipidemic serum).

Many common individual protein components were identified in the BCs, therefore, we next evaluated whether ball milling influences their abundance in the SWCNT BC. Differences in protein abundances for proteins found in common between As Prepared and defective ball milled SWCNTs following incubation in either normal (Supplemental Table 5) or hyperlipidemic (Supplemental Table 6) serum were evaluated. Representative data comparing the abundance of commonly associated proteins between As Prepared and 2 h ball milled SWCNTs following incubation in normal or hyperlipidemic serum is shown in Fig. 3. Ball milling resulted in the decreased abundance of many of these common proteins (higher ratios of As Prepared: 2 h ball milled). The abundance of many proteins associated with the complement system were altered in the BC that formed on 2 h ball milled SWCNTs compared to As Prepared SWCNTs in normal serum suggesting modified interactions with the complement system. The complement system is a part of the innate immune system and is involved in the removal of foreign objects as well as the inflammatory response^35^. These alterations in the interactions with the complement system due to the presence of defects in ball milled SWCNTs may result in modified activation of the immune system thereby altering the SWCNT inflammatory response and clearance. Further, many changes in protein abundance due to ball milling of SWCNTs and incubation in hyperlipidemic serum involved various immunoglobulins. Specifically, fewer immunoglobulins were found to associate with SWCNTs following ball milling after incubation in hyperlipidemic serum. This finding implies that ball milled SWCNTs with higher defects are less likely to interfere with the adaptive immune response and less likely to trigger an immune response. In summary, although many proteins were determined to commonly associate with SWCNTs, defects induced by ball milling influenced their abundance within the BC and may ultimately influence cellular responses.

**Figure 4:**
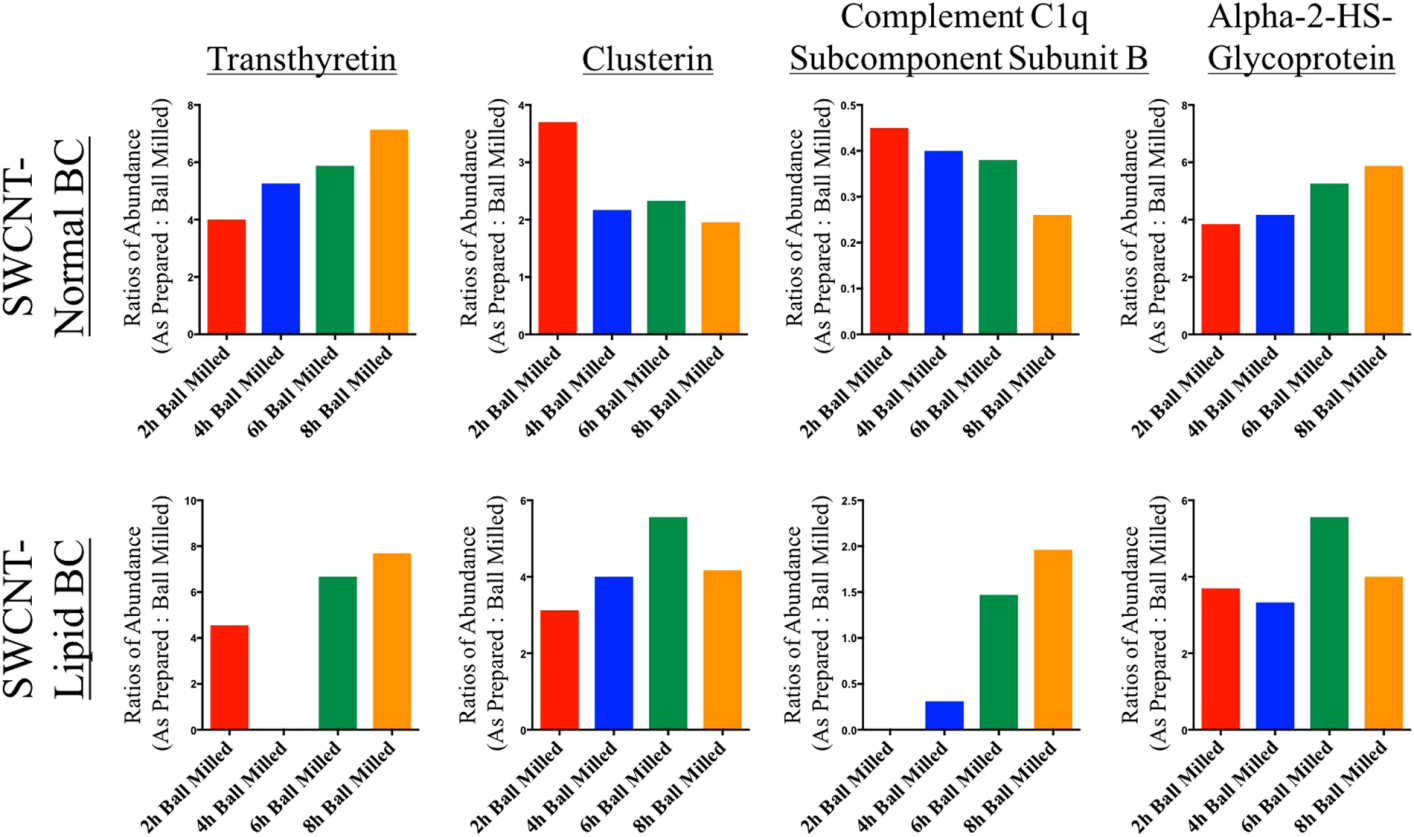
Differences in relative abundance of specific proteins (Transthyretin, Clusterin, Complement C1q Subcomponent Subunit B, and Alpha-2-HS-Glycoprotein) found to associate with SWCNTs in either normal or hyperlipidemic serum due to ball milling time. All ratios are As Prepared SWCNTs: Ball Milled SWCNTs (n = 4/group).

Progressive alterations in individual protein abundance due to ball milling time and increase in defects were also analyzed (Fig. 4). For example, as ball milling time and defects increased, the amount of transthyretin was found to decrease on the surface of SWCNTs following incubation in normal or hyperlipidemic serum. Transthyretin is produced by the liver and is a carrier of the thyroid-derived hormone throxine which is responsible for metabolism regulation^36^. Our data suggests that As Prepared SWCNTs may result in increased disruption of the endocrine system by binding transthyretin and interfering with the transport of thyroxine as compared to defective ball milled SWCNTs. In addition, increased ball milling time led to increased association of clusterin (molecular chaperone protein and lipid transport)^37^ and complement C1q subcomponent subunit B (complement cascade) following incubation in normal serum but decreased association following incubation in hyperlipidemic serum. This data demonstrates that depending on the physiological environment ball milling differentially affects association of individual proteins. Increased ball milling time corresponded to decreased association of alpha-2-HS-glycoprotein abundance following incubation in normal serum. Since alpha-2-HS-glycoprotein is a large and abundant protein in serum it is likely that as its association decreases in the BC it is being replaced by smaller and lesser abundant proteins. This enrichment of lesser abundant proteins may alter the cellular interactions of the BC. Ultimately, these representative changes in commonly associated individual protein abundance demonstrate differences in the BC of the SWCNT that are dependent on the process of ball milling. These abundance changes and alterations in protein identities support differential biological interactions due to ball milling and the likelihood of variable cellular and physiological responses.

Proteins found to associate with As Prepared and ball milled SWCNTs incubated in either normal or hyperlipidemic serum were evaluated for biological pathways and common protein characteristics via gene ontology (Supplemental Table 9). This assessment of the data was performed to examine specific pathways and protein characteristics which may be potentially altered due to ball milling of SWCNTs. As Prepared SWCNTs incubated in normal serum significantly interacted with the biological pathways of blood coagulation and the plasminogen activating cascade. Other proteins interacting with the As Prepared SWCNTs in normal serum are involved in the pathways of Huntington’s disease, integrin signaling pathway, vitamin D metabolism, and others (Supplemental Table 9). Following ball milling, SWCNTs incubated in normal serum associated additional unique proteins, which were also significantly associated with the pathways of blood coagulation, and the plasminogen activating cascade. Specifically, the addition of defects by ball milling increased the number of proteins involved with the biological pathways of blood coagulation, the plasminogen activating cascade, integrin signaling, inflammation mediated by chemokine and cytokine signaling, and others (Supplemental Table 9). Overall, the unique proteins associated with SWCNTs following ball milling only added more proteins to pathways already identified in the As Prepared sample. For example, 11 proteins associated with blood coagulation were found in As Prepared SWCNT samples, while samples with defects introduced by ball milling were found to associate with 13 proteins. Specifically, ball milled SWCNTs where found to associate alpha-1-antitrypsin, glycoprotein 1b platelet alpha subunit, and fibrinogen beta chain uniquely while the As Prepared SWCNT uniquely associated coagulation factor XIII B chain. Overall, these findings suggest that ball milling increases the interactions with pathways SWCNTs already may perturb prior to ball milling. As Prepared SWCNTs incubated in hyperlipidemic serum interacted with proteins that were significantly related to pathways of blood coagulation, and the plasminogen activating cascade (Supplemental Table 9). Ball milling resulted in increased association of proteins related to the biological pathways of blood coagulation, integrin signaling, plasminogen activating cascade (Supplemental Table 9). The process of ball milling also resulted in interactions with proteins related to unique pathways compared to the As Prepared sample. Ball milled specific pathways following incubation in hyperlipidemic serum include cadherin signaling, Alzheimer disease-presenilin, Huntington disease, inflammation-mediated by chemokine and cytokine signaling, cytoskeletal regulation by Rho GTPase, and Nicotinic acetylcholine receptor signaling (Supplemental Table 9). These findings demonstrate that in hyperlipidemic serum the process of ball milling enhances SWCNT interactions with proteins involved in biological pathways while also associating proteins that are related to distinct biological pathways.

To examine commonalities between proteins that associate with the surface of SWCNTs and the role of ball milling, gene ontology was again utilized to assess protein classes (Supplemental Table 9). In normal serum, As Prepared SWCNTs associated protein classes involved in enzyme modulation, transfer/carrier, hydrolase, defense/immunity, signaling, receptors and others (Supplemental Table 9). Following ball milling and incubation in normal serum SWCNTs associated an increased number of proteins related to enzyme modulation, defense/immunity, signaling, hydrolase and others. Following ball milling, SWCNTs also interacted with proteins classified as structural while As Prepared SWCNTs did not associate with structural proteins (Supplemental Table 9). Incubation of As Prepared SWCNTs in hyperlipidemic serum resulted in association of protein classes of enzyme modulation, hydrolase, transfer/carrier, receptor, defense/immunity, signaling, extracellular matrix, cytoskeletal, and others. Following the process of ball milling, additional numbers of unique proteins from classes of enzyme modulation, transfer/carrier, defense/immunity, signaling, and cytoskeletal were found to associate. Further, following ball milling, SWCNTs were determined to also associate with structural proteins in hyperlipidemic serum while the As Prepared SWCNTs did not associate with any structural proteins (Supplemental Table 9). These findings demonstrate that following ball milling, SWCNTs incubated in normal serum interact with primarily with the same classes of proteins as the As Prepared SWCNTs. The unique proteins found to interact with SWCNTs following ball milling (Fig. 3) consist of additional proteins from these classes. Ball milling however appears to increase interactions with structural proteins.

### Proteomic Evaluation of the Variations in Biocorona Formation on SWCNTs due to Hyperlipi-demia

A substantial portion of our population exist with an underlying health condition. These underlying conditions often modify the biomolecular content of the individual’s serum. Since formation of the BC is dependent on both the physicochemical properties of the engineered nanomaterial as well as the physiological environment, it is likely that underlying disease states will result in unique BC formation^26^. One highly prevalent and growing disease state is hyperlipidemia or high cholesterol. Previous evaluation has demonstrated that incubation of iron oxide nanoparticles in hyperlipidemic serum results in the formation of unique BCs compared to incubation in normal serum^26^. Further, addition of the hyperlipidemic BC was determined to induce greater inflammatory responses in endothelial cells suggesting increased toxicity of nanoparticles in individuals with hyperlipidemia. In our current study, we evaluated the protein components of the BC that forms on SWCNTs that have been either untreated or ball milled in both normal or hyperlipidemic serum. In order to fully understand nanoparticle-biomolecular interactions, it is necessary to examine common disease states.

**Figure 5:**
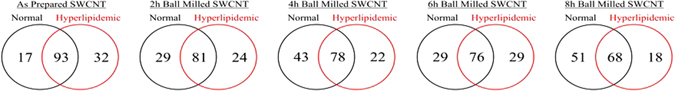
Venn diagrams representing the number of proteins found to associate with SWCNTs due to incubation in either normal or hyperlipidemic serum (n = 4/group).

A proteomic comparison of the protein components of the BC that forms on SWCNTs following incubation in either normal or hyperlipidemic serum demonstrated that the majority of proteins were in common (Fig. 5 and Supplemental Table 7). These common proteins included apolipoprotein AI, C reactive protein, fibronectin, kinnogen-1, plasminogen, and others. Unique to the normal serum BC were proteins alpha-1 antitrypsin 1–4, hemoglobin subunit A, and others. Unique to the hyperlipidemic BC were hepcidin, extracellular matrix protein 1, antithrombin-III, and others. Interestingly, as ball milling time was increased, less proteins were shared between the normal and hyperlipidemic serum BCs. This corresponded with a trend of more unique proteins in the normal serum BC compared to the hyperlipidemic serum BC as ball milling time increased (Fig. 5). Further, the hyperlipidemic serum BC demonstrated less unique proteins as compared to the normal serum BC as ball milling time increased. It is likely that due to the amount of cholesterol present in the hyperlipidemic serum, that cholesterol outcompeted proteins for binding sites on the SWCNTs. This competition may explain the decreases in common proteins between the normal and hyperlipidemic BCs, as increased ball milling time enhanced the surface area of SWCNTs resulting in more cholesterol association. To date, few studies have evaluated the lipid content of the BC that forms on engineered nanomaterials. Future research is needed regarding the association of lipids on engineered nanomaterials in physiological environments^38^. It is likely that they not only modify the association of other biomolecules but also affect the nanomaterial-cellular interactions.

**Figure 6:**
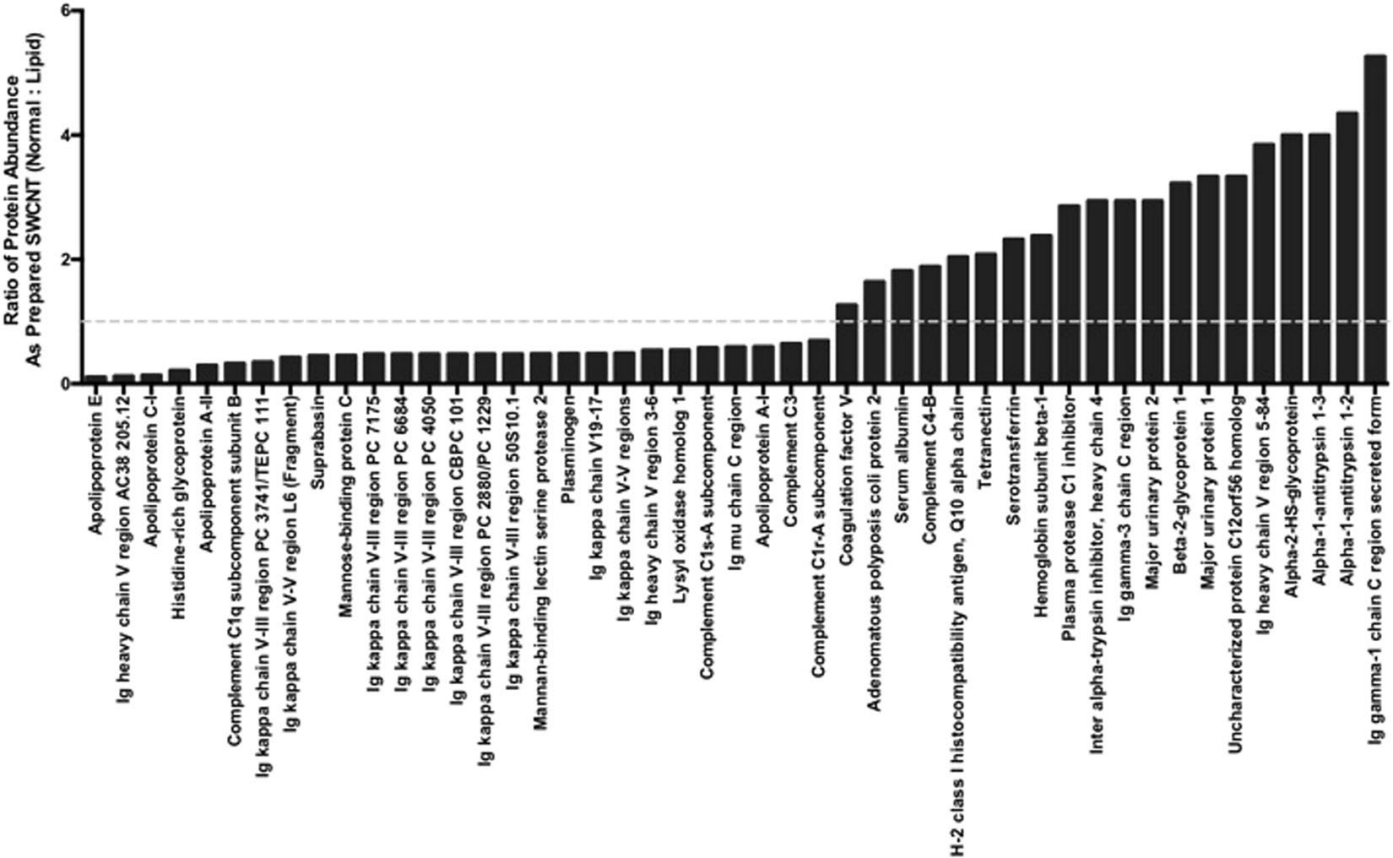
Relative abundance of proteins found to associate with As Prepared SWCNTs due to incubation in either normal or hyperlipidemic serum (n = 4/group). Additional data for other ball milled SWCNT samples can be found in Table S8.

Although a majority of proteins were shared between the normal and hyperlipidemic BCs on SWCNTs, differences were determined regarding their abundance (Fig. 7 and Supplemental Table 8). Many proteins such as immunoglobulin kappa V-III, apolipoproteins E, C-I, A-II, and A-I, and complement C1q subcomponent subunit B, complement C1s-A subcomponent, complement C3 and complement C1r-A subcomponent were found to be enriched within the hyperlidemic BC on As Prepared SWCNTs. As Prepared SWCNTs were found to bind increased amounts of coagulation factor V, serum albumin, serotransferrin, alpha-2-HS-glycoprotein, and alpha-1-antitrypsin 1 (Fig. 6). As Prepared SWCNTs in hyperlipidemic serum associated greater amounts of apolipoproteins and complement factors likely due to their increased abundance in hyperlipidemic serum. Recently, it has also been demonstrated that nanoparticles can associate biomolecules such as lipoproteins in a manner that results in the presentation of functional motifs allowing for recognition by low-density lipoprotein receptors^39^. Our current study suggests that in diseases such as hyperlipidemia, where lipoprotein content is elevated and is more likely to bind the nanoparticle, that interactions with specific lipoprotein receptors may be increased. Many of the shared proteins found to be decreased in the hyperlipidemic compared to the normal BC appear to be high abundant serum proteins. This suggests that the increased lipid content maybe competing with them for binding sites on the SWCNTs. Further, this data demonstrates that the BC is not only different due to structural modification induced by ball milling but also due to the physiological environment. Although, they share numerous proteins the abundance of these proteins are not uniform. These variations in abundance further demonstrate altered biomolecular interactions, distinct BC formation, and the possibility for modified cellular responses. Data regarding the abundance differences of shared proteins for ball milled SWCNTs in normal or hyperlipidemic serums can be found in Supplemental Table 8.

To further interrogate proteomic interactions in our study, gene ontology was used to evaluate proteins related to specific biological pathways and classes of proteins that associated with SWCNTs. For this comparison of biological pathways and protein classes, all proteins that were found to associate under either serum condition regardless of the SWCNT sample were compiled into lists for analysis. SWCNTs in either normal or hyperlipidemic serum were both found to significantly associate proteins related to the pathways of blood coagulation and the plasminogen activating cascade (Supplemental Table 10). Other pathways identified in common include integrin signaling, inflammation mediated by chemokine and cytokine signaling, and others (Supplemental Table 10). The hyperlipidemic BC, on SWCNTs however uniquely associated glyceraldehyde-3-phosphate dehydrogenase which is involved in the biological pathway of glycolysis. Although most of the proteins that associated with SWCNTs in normal and hyperlipidemic serums were associated with the same biological pathways, it is interesting that there were unique proteins that bound to the SWCNTs. Specifically, in terms of the blood coagulation pathway, the normal BC uniquely associated glycoprotein 1b platelet alpha subunit while the lipid BC uniquely associated anti 2-antiplasmin and alpha-1-antitrypsin. To determine how serum conditions, alter the types of proteins that associate with SWCNTs a similar evaluation of protein characteristics was performed. Classes of proteins that were identified in common between the normal and lipid BCs were enzyme modulation, transfer/carrier, defense/immunity, signaling, transporter, receptor and others (Supplemental Table 10). In comparison to normal serum, SWCNTs incubated in hyperlipdemic serum were found to associate less proteins related to enzyme modulation, signaling, hydrolase, and extracellular matrix while binding more proteins related to transfer/carrier, cytoskeletal, transporter, oxidoreductase, isomerase, transcription factor, and nucleic acid binding. For example, the lipid BC was found to associate with single proteins from the classes of transcription factor and nucleic acid binding (transcription factor HIVEP2), and isomerase (major urinary protein-1). Again, this assessment demonstrated that although proteins that bound to the SWCNTs were from similar protein classes, specific proteins that bound to SWCNTs were unique. Also, these data further highlight the differences in the formation of the BC due to alterations in the environment.

## Conclusions

Defects in nanomaterials can play an important role in determining biomolecular interactions with serum proteins. SWCNTs with different defect densities were prepared by the ball milling process to study the influence of defects on BC formation. Our study demonstrates that charged defects induced by the ball milling process result in the formation of distinctive BCs through the differential association of proteins. Specifically, proteins that function through charge transfer and redox mechanisms were observed to bind to the defected SWCNTs. A common disease-induced alteration in the serum environment can also result in modified BC formation. Further, these disease-related changes in the physiological environment demonstrate differences due to ball milling when compared to normal conditions. Toxicological experimentation of exposures to nanomaterials such as SWCNTs often focus on end product exposure and healthy/normal physiological conditions. However, nanomaterials are often modified through processes such as ball milling which may influence nanomaterial-biomolecule interactions. Future research in our laboratory intends to understand the toxicological impact of the alterations in the SWCNT-BC described in this study. Exposures also are likely to increase in individuals with underlying disease states as they become a more significant proportion of our population. In order to protect the population from unintended adverse responses, it is necessary to understand how these modifications in the nanomaterial’s surface and the biological environment can alter nanomaterial-biomolecule interactions which may govern biological responses such as toxicity, biodistribution, and clearance.

## Materials and Methods

### Animals

C57BL/6J (Stock No: 000664) and B6.129S7-Ldlr^tm1Her^/J (Stock No: 002207) mice were acquired from Jackson Laboratories (Bar Harbor, ME, USA) at 12 weeks of age. Breeding pairs of B6.129S7-Ldlr^tm1Her^/J mice were established and maintained at the University of Colorado Anschutz Medical Campus. B6.129S7-Ldlr^tm1Her^/J mice are homozygous for the Ldlr^tm1Her^ mutation resulting in a knockout of the low-density lipoprotein receptor. B6.129S7-Ldlr^tm1Her^/J pups were weaned 4 weeks following birth and placed on a high cholesterol (1%) western diet (Catalog No. 5TJT, TestDiet, St. Louis MO, USA). Briefly, this diet has a protein to fat ratio of 2:5 and has 1% total cholesterol added. B6.129S7-Ldlr^tm1Her^/J mice were allowed the high cholesterol (1%) western diet and water ad libitum for 14 weeks. At 18 weeks of age C57BL6/J and B6.129S7-Ldlr^tm1Her^/J mice were anesthetized with an overdose of ketamine/xylazine (300 mg/kg) via i.p. injection and blood was collected by cardiac puncture. Serum was then isolated from blood by centrifugation (3,500 g at 4 °C for 10 min). Serum samples were then pooled according to animal strain and stored at −80 °C. C57BL/6J were utilized to produce the normal serum whereas B6.129S7-Ldlr^tm1Her^/J mice were utilized to produce the hyperlipidemic serum. Serum from C57BL/6 J mice fed a normal diet demonstrated a cholesterol content of 3.43 ± 1.89 mg/ml whereas B6.129S7-Ldlr^tm1Her^/J mice fed a high cholesterol western diet to produce hyperlipidemic serum demonstrated a cholesterol content of 34.39 ± 7.01 mg/ml. C57BL/6J are the appropriate control or normal serum strain for comparison as they are the genetic background for the B6.129S7-Ldlr^tm1Her^/J. All animal procedures were conducted in accordance with the National Institutes of Health Guidelines and approved by the University of Colorado Anschutz Medical Campus Institutional Animal Care and Use Committee. All animals were treated humanely and with regard for alleviation of suffering.

### Synthesis and characterization of SWCNTs

SWCNTs were prepared using chemical vapor deposition^27^. A MTI planetary ball mill was used to induced defects in SWCNTs. High-resolution transmission electron micrographs were obtained using Hitachi H-9600. A Renishaw InVia micro-Raman (coupled to 514.5 nm Ar + laser) and Bruker IFS V/66 FT-Raman equipped with Nd:YAG 1064 nm were used to obtain the Raman and PL spectra of SWCNTs. The surface area of SWCNTs was characterized using Quantachrome Autosorb instrument. All the spectroscopic measurements were performed in triplicates.

### Formation of the SWCNT-Biocoronas

BCs were formed on SWCNTs (n = 4/group) using a modified Tenzer’s method, as described in our recent publications and publications by other laboratories^12, 30, 40, 41^. Individual SWCNTs were suspended water at a concentration of 1 mg/ml and incubated in either 10% normal or 10% hyperlipidemic serum while being mixed constantly for 8 h at 4 °C. SWCNTs were incubated with proteins for 8 h to ensure a stable equilibrium was reached and at 4 °C to prevent any entropy-driven or thermally induced unfolding of proteins. Specifically, SWCNTs following suspension were sonicated for 5 min in an inverted cup-horn sonicator at an amplitude of 75. 250 µl of SWCNTs (1 mg/ml), 650 µl of water, and 100 µl of serum were combined in a 1.5 ml tube. Following the 8 h incubation SWCNTs were pelleted via centrifugation 14,000 rpm (20,817 × g) 10 min and washed with PBS. This washing process was repeated three times. Following the final centrifugation, the supernatant was removed and SWCNTs were resuspended with 250 µl of water to their initial concentration of 1 mg/ml. The BC formed following incubation of SWCNTs in normal serum is referred to as SWCNT-Normal BC whereas the BC formed following incubation in hyperlipidemic serum is referred to as SWCNT-Lipid BC. This study was specifically designed to investigate differences in the BC due to SWCNT defects and alterations in serum conditions. An 8 h incubation of SWCNTs was selected to allow for the BCs on the surface of SWCNTs to reach an equilibrium. Future studies are required to evaluate time dependent alterations in formation of the BC due to defects and alterations in serum content as it is likely that the kinetics of interactions differ in these scenerios^42–44^.

### Evaluation of the Protein Components of the SWCNT-Biocoronas

Utilizing a proteomics approach, individual protein components of the BCs were identified similar to our previous studies evaluating the BC^26^. Briefly, following addition of the BCs, SWCNTs were washed and pelleted three times with PBS (10 min 14,000 rpm/20,817 × g). Proteins were then solubilized using 8 M urea and 10 mM dithiothreitol (DTT) for 45 min at 60 °C. SWCNTs were then pelleted via centrifugation at 15,000 × g for 10 min and supernatant containing solubilized proteins was collected. Protein samples were then treated with 30 mM iodoacetamide for 30 min in the dark at room temp followed by the addition of 10 mM DTT in 50 mM ammonium bicarbonate (ABC) for 30 min at room temperature. Finally, 50 mM ABC was used to dilute remaining urea to less than 1 M. Samples were then proteolyzed using porcine trypsin (0.2 ng/μl) overnight at 37 °C, heated to 90 °C to deactivate trypsin, and concentrated to dryness. Samples were then resuspended in 0.1% trifluoroacetic acid in water, purified via Ziptip Pipette Tips (Millipore, Billerica, MA), and concentrated to dryness. Samples were then resuspended in 0.1% Formic Acid in HPLC-grade water and injected onto a nHPLC utilizing a flow rate of 800 nL/min with a gradient of 5–50% 0.1% formic acid in acetonitrile (ACN) over 30 min on C 18 trapping (20 × 0.1 mm^2^) and analytical columns (150 × 0.1 mm^2^). The nLC was coupled to a nano-ESI source and Impact HD Q-TOF mass spectrometer (Bruker Daltonics, Inc., Billerica, MA). The acquired data were searched using a MASCOT (ver. 2.4.1) search engine with a peptide cutoff of 10 ppm and a minimum peptide score of 20 and a protein score of 40. The false discovery rate for samples was <1% and the significance threshold was p < 0.05. These identified proteins were then assessed for biological pathways SWCNTs may interfere with and common protein characteristics by via the Gene Ontology Consortium database (geneontology.org). Briefly, all proteins found to associate with the As Prepared SWCNT sample following incubation in either normal or hyperlipidemic serum were uploaded into geneontology’s enrichment analysis tool, biological process and Mus Musculus were selected. Mapped identifiers were then used within the PANTER Pathway and PANTHER Protein Class tool to identify pathways and protein characteristics shared by the proteins associated with the As Prepared sample. Evaluation of how SWCNT defects resulting from ball milling may modify these interactions was performed by producing a single list of all unique proteins that absorbed following ball milling. This list was then analyzed in the same way as the As Prepared protein list was assessed. A similar procedure using gene ontology was performed to understand differences in biological pathways and protein characteristics for SWCNTs to compare differences in the BC due to incubation in normal or hyperlipidemic serum. All analysis utilizing gene ontology used the Bonferroni correction for multiple testing with statistical significance determined by p < 0.05. To determine differences in abundance of common protein components of the BCs, ratios of individual proteins were assessed utilizing ProteinScape and ProfileAnalysis software packages (Bruker Daltonic, Billerica, MA). Four samples of each BC were analyzed individually and injected 4 separate times. Peptides to be considered for evaluation had to be found within 5 of 8 samples with a minimum of 2 per BC type. Only peptides determined to be statistically significant were utilized to calculate ratios and more than one peptide had to be identified for a protein for that individual protein to be quantified. All relative quantification data are presented as ratios in the supplemental files along with the coefficient of variation percentages for each comparison. Data generated allowed for comparisons of the BCs between SWCNTs that had undergone ball milling resulting in altered defect loads and comparisons between the BCs that formed under the differing physiological conditions (normal vs hyperlipidemic).

### Data Availability

All data is available upon request.

## Electronic supplementary material

Supplemental Dataset
