# Supplementary material for: Variations in biocorona formation related to defects in the structure of single walled carbon nanotubes and the hyperlipidemic disease state
Source: Sci Rep. 2017 Aug 16;7:8382. doi: 10.1038/s41598-017-08896-w (PMC5559455; doi:10.1038/s41598-017-08896-w)
Supplement: Supplementary file 1 — Supplemental Dataset [file 41598_2017_8896_MOESM1_ESM.pdf]

Supplemental Information:

Variations in biocorona formation related to defects in the structure of single walled carbon nanotubes and the hyperlipidemic disease state

Achyut J. Raghavendra,[1,2] Kristofer Fritz,[3] Sherleen Fu,[4] Jared M. Brown,[5] Ramakrishna Podila,[1,2*] Jonathan H. Shannahan[4*]

1 Department of Physics and Astronomy, Clemson University, Clemson, South Carolina, 29634, USA

2 Clemson Nanomaterials Center and COMSET, Clemson University, Anderson, South Carolina, 29625, USA

3 Department of Pharmaceutical Sciences, Skaggs School of Pharmacy and Pharmaceutical Sciences, The University of Colorado Anschutz Medical Campus, Aurora, Colorado, 80045, USA

4 School of Health Sciences, College of Human and Health Sciences, Purdue University, West Lafayette, IN 47907, USA

5 Colorado Center for Nanomedicine and Nanosafety, Skaggs School of Pharmacy and Pharmaceutical Sciences, The University of Colorado Anschutz Medical Campus, Aurora, Colorado, 80045, USA

* Corresponding Authors:

Jonathan Shannahan, School of Health Sciences, Purdue University, 550 Stadium Mall Dr. 47907 West Lafayette Indiana, USA; jshannah@purdue.edu; 765-494-2326

Ramakrishna Podila, Department of Physics and Astronomy and Clemson Nanomaterials Center and COMSET, Clemson University, Anderson, South Carolina, 29625, USA; rpodila@g.clemson.edu; 864-656-0805

Supporting Information Legends

Supplemental Figure 1. Transmission electron microscopy (TEM) images of SWCNTs As Prepared and following ball milling. (A) Low magnification and (B) high magnification TEM images of As Prepared SWCNTs. TEM images of SWCNTs following ball milling for 2h (C), 4h (D), 6h (E), or 8h (F).

Supplemental Table 1. Identification of all proteins found to associate in the biocorona of each SWCNT following incubation in normal serum by label-free mass spectrometry.

Supplemental Table 2. Identification of all proteins found to associate in the biocorona of each SWCNT following incubation in hyperlipidemic serum by label-free mass spectrometry.

Supplemental Table 3. Comparison of proteins identified to associate with As Prepared SWCNTs or ball milled SWCNTs with defects in normal serum.

Supplemental Table 4. Comparison of proteins identified to associate with As Prepared SWCNTs or ball milled SWCNTs with defects in hyperlipidemic serum.

Supplemental Table 5. Quantification of commonly associated proteins of the biocorona on SWCNTs following incubation in normal serum.

Supplemental Table 6. Quantification of commonly associated proteins of the biocorona on SWCNTs following incubation in hyperlipidemic serum.

Supplemental Table 7. Comparison of proteins identified to associate with SWCNTs or ball milled SWCNTs with defects following incubation in either normal or hyperlipdemic serum.

Supplemental Table 8. Quantification of commonly associated proteins of the biocorona on SWCNTs following incubation in either normal or hyperlipidemic serum.

Supplemental Table 9. Categorization of biological pathways and protein characteristics of the biocorona on SWCNTs due to ball milling utilizing gene ontology PANTHER pathway and protein class analysis tools.

Supplemental Table 10. Categorization of biological pathways and protein characteristics of the biocorona on SWCNTs due to differences in serum utilizing gene ontology PANTHER pathway and protein class analysis tools.

Supplemental Figure 1.

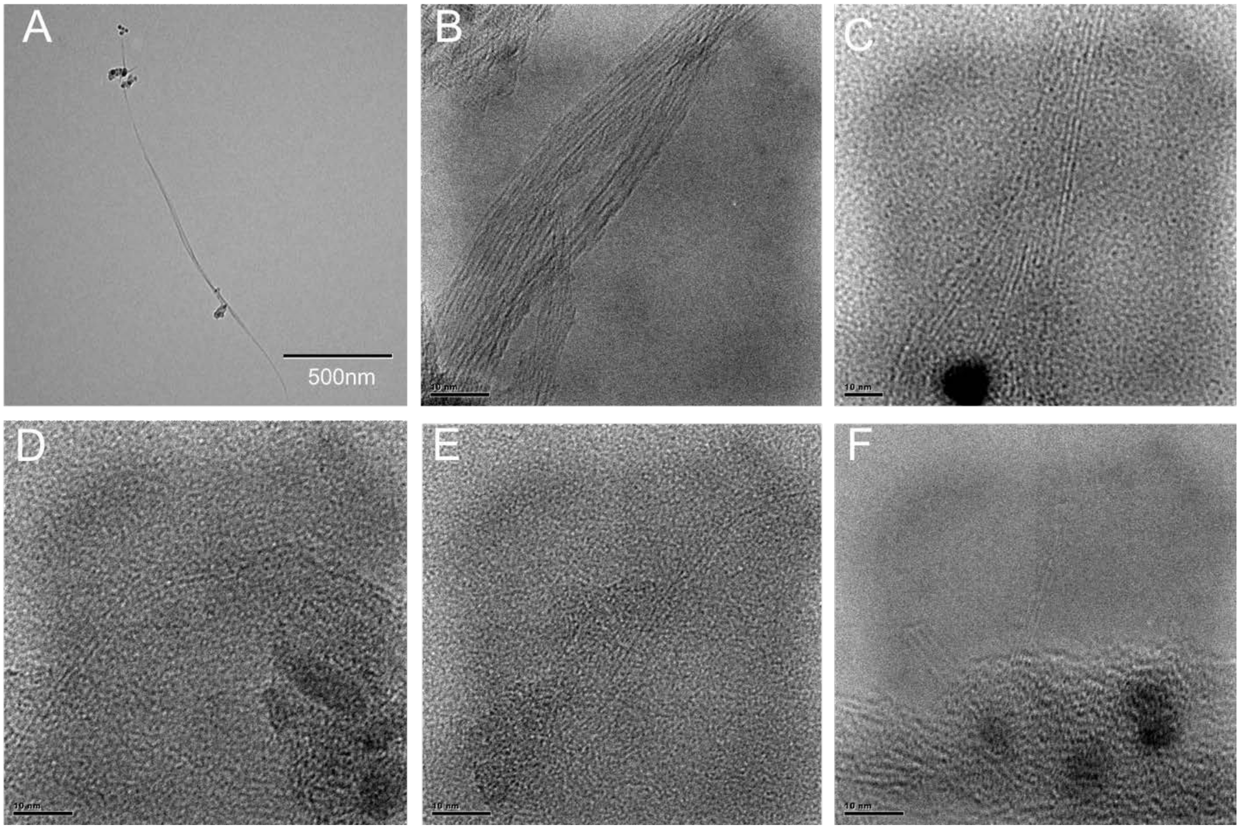

Supplemental Table 1. All Identified Proteins on SWCNTs following Incubation in Normal Serum

As Prepared SWCNTs

Accession	Name	Meta Score A	Meta Score B	Peptides A	Peptides B	SC [%] A	SC [%] B	RMS90 [ppm] A	RMS90 [ppm] B	Rank A	Rank B
ACTB_MOUSE	Actin, cytoplasmic 1 OS=Mus musculus GN=Actb PE=1 SV=1	59.2	52	2	1	7.2	4.3	6.33	3.38	107	121
A1AG1_MOUSE	Alpha-1-acid glycoprotein 1 OS=Mus musculus GN=Orm1 PE=1 SV=1	72	101.9	1	1	7.2	7.2	3.02	0.28	102	97
A1AT2_MOUSE	Alpha-1-antitrypsin 1-2 OS=Mus musculus GN=Serpina1b PE=1 SV=2	691	790.1	12	11	36.6	38	4.51	4.88	17	21
A1AT3_MOUSE	Alpha-1-antitrypsin 1-3 OS=Mus musculus GN=Serpina1c PE=1 SV=2	749.9	868.3	12	12	36.7	40.5	3.9	4.72	15	16
FETUA_MOUSE	Alpha-2-HS-glycoprotein OS=Mus musculus GN=Ahsg PE=1 SV=1	713.3	1281	10	15	35.4	50.7	4.02	3.32	16	11
ANT3_MOUSE	Antithrombin-III OS=Mus musculus GN=Serpinc1 PE=1 SV=1	50.5	92	1	2	2.4	7.1	7.58	7.03	113	101
APOA1_MOUSE	Apolipoprotein A-I OS=Mus musculus GN=Apoa1 PE=1 SV=2	964.8	1357.8	18	22	66.3	69.3	3.29	4.35	11	10
APOA2_MOUSE	Apolipoprotein A-II OS=Mus musculus GN=Apoa2 PE=1 SV=2	56.5	84.9	1	2	9.8	14.7	1.81	1.47	110	107
APOA4_MOUSE	Apolipoprotein A-IV OS=Mus musculus GN=Apoa4 PE=1 SV=3	681.8	903	13	13	46.6	47.3	4.59	4.26	18	15
APOC1_MOUSE	Apolipoprotein C-I OS=Mus musculus GN=Apoc1 PE=1 SV=1	81.9	89.8	2	2	20.5	20.5	2.97	5.48	98	102
APOC2_MOUSE	Apolipoprotein C-II OS=Mus musculus GN=Apoc2 PE=2 SV=1	178.6	270.7	3	4	30.9	33	3.94	4.95	70	59
APOC3_MOUSE	Apolipoprotein C-III OS=Mus musculus GN=Apoc3 PE=1 SV=2	342.7	397.3	5	5	50.5	50.5	3.89	6.21	40	41
APOE_MOUSE	Apolipoprotein E OS=Mus musculus GN=Apoe PE=1 SV=2	409.3	512.3	9	10	26	40.8	2.87	2.59	33	33
APOH_MOUSE	Beta-2-glycoprotein 1 OS=Mus musculus GN=Apoh PE=1 SV=1	276.1	857.7	8	15	29	46.7	5.22	3.28	48	17
B2MG_MOUSE	Beta-2-microglobulin OS=Mus musculus GN=B2m PE=1 SV=2	0	155	0	4	0	42	0	3	0	80
CRP_MOUSE	C-reactive protein OS=Mus musculus GN=Crp PE=1 SV=2	375.3	270	8	7	36.4	35.1	3.35	4.72	35	60
C4BPA_MOUSE	C4b-binding protein OS=Mus musculus GN=C4bpa PE=1 SV=3	267.4	334.7	5	8	16	23	5.98	3.51	52	44
CD5L_MOUSE	CD5 antigen-like OS=Mus musculus GN=Cd5l PE=1 SV=3	1474.7	1245.5	23	20	58	56.5	3.98	3.44	7	12
CERU_MOUSE	Ceruloplasmin OS=Mus musculus GN=Cp PE=1 SV=2	0	56.4	0	1	0	0.8	0	4.34	0	119
CLUS_MOUSE	Clusterin OS=Mus musculus GN=Clu PE=1 SV=1	138.7	788.3	3	14	11.4	32.4	4.78	2.45	76	22
FA5_MOUSE	Coagulation factor V OS=Mus musculus GN=F5 PE=1 SV=1	588.7	361.6	14	7	8.5	4.1	4.96	3.59	21	43
FA12_MOUSE	Coagulation factor XII OS=Mus musculus GN=F12 PE=1 SV=2	0	153	0	3	0	8	0	3.56	0	81
F13B_MOUSE	Coagulation factor XIII B chain OS=Mus musculus GN=F13b PE=1 SV=2	86.6	275.2	3	8	6.9	20.2	4.36	4.87	96	58
C1QA_MOUSE	Complement C1q subcomponent subunit A OS=Mus musculus GN=C1qa PE=1 SV=2	282	144.5	6	2	29	11.8	3.97	7.28	47	87
C1QB_MOUSE	Complement C1q subcomponent subunit B OS=Mus musculus GN=C1qb PE=1 SV=2	371.5	211.8	5	4	21.7	16.6	2.56	2.59	37	71
C1QC_MOUSE	Complement C1q subcomponent subunit C OS=Mus musculus GN=C1qc PE=1 SV=2	300.4	122.4	4	2	21.5	11.4	6.36	3.44	45	92
C1RA_MOUSE	Complement C1r-A subcomponent OS=Mus musculus GN=C1ra PE=1 SV=1	933	822.7	17	16	42.1	40.7	3.91	4.53	12	19
CS1A_MOUSE	Complement C1s-A subcomponent OS=Mus musculus GN=C1sa PE=2 SV=2	906.1	614.4	17	11	36.2	25.6	4.46	5.19	13	28
CO3_MOUSE	Complement C3 OS=Mus musculus GN=C3 PE=1 SV=3	2717.1	2923.1	56	53	40.6	40	3.9	3.1	3	2
CO4B_MOUSE	Complement C4-B OS=Mus musculus GN=C4b PE=1 SV=3	1943.7	1749.4	38	37	31.2	32.2	3.88	3.5	6	7
CFAB_MOUSE	Complement factor B OS=Mus musculus GN=Cfb PE=1 SV=2	234.5	278.9	6	6	11	13.3	3.68	3.35	60	55
CFAD_MOUSE	Complement factor D OS=Mus musculus GN=Cfd PE=1 SV=1	0	61	0	2	0	10	0	2.73	0	118
CFAH_MOUSE	Complement factor H OS=Mus musculus GN=Cfh PE=1 SV=2	1425.6	2042.6	34	39	35.8	43.7	4.14	3.71	8	4
CFAI_MOUSE	Complement factor I OS=Mus musculus GN=Cfi PE=1 SV=3	43.9	113.3	1	3	2.2	5	4.57	3.87	119	95
ECM1_MOUSE	Extracellular matrix protein 1 OS=Mus musculus GN=Ecm1 PE=1 SV=2	0	61.2	0	2	0	4.8	0	4.99	0	117
FETUB_MOUSE	Fetuin-B OS=Mus musculus GN=Fetub PE=1 SV=1	59.5	141.2	1	3	2.3	12.4	6.57	4.18	106	88
FIBA_MOUSE	Fibrinogen alpha chain OS=Mus musculus GN=Fga PE=1 SV=1	207.5	321.7	4	5	7.5	13.2	6.76	3.2	65	46
FIBG_MOUSE	Fibrinogen gamma chain OS=Mus musculus GN=Fgg PE=1 SV=1	272.4	302.7	8	7	31.4	24.1	3.42	4.59	51	50
FINC_MOUSE	Fibronectin OS=Mus musculus GN=Fn1 PE=1 SV=4	572.1	317.5	14	8	9.4	4	4.54	3.32	23	48
GELS_MOUSE	Gelsolin OS=Mus musculus GN=Gsn PE=1 SV=3	761.6	1100.6	17	24	29.6	42.6	3.6	4.14	14	13
G3P_MOUSE	Glyceraldehyde-3-phosphate dehydrogenase OS=Mus musculus GN=Gapdh PE=1 SV=2	49.5	50.3	2	2	10.8	7.8	4.73	6.79	114	123
HA10_MOUSE	H-2 class I histocompatibility antigen, Q10 alpha chain OS=Mus musculus GN=H2-Q10 PE=1 SV=3	124	725.9	3	14	11.7	44.3	2.74	4.72	81	24
HPT_MOUSE	Haptoglobin OS=Mus musculus GN=Hp PE=1 SV=1	97.4	140.3	3	4	11.8	15.9	5.95	4.73	93	89
HBB1_MOUSE	Hemoglobin subunit beta-1 OS=Mus musculus GN=Hbb-b1 PE=1 SV=2	48.3	89.5	1	3	6.8	30.6	2.04	8.11	117	103
HEMO_MOUSE	Hemopexin OS=Mus musculus GN=Hpx PE=1 SV=2	553.2	1060.2	12	22	35.2	50.9	4.38	4.1	25	14
HEPC_MOUSE	Hepcidin OS=Mus musculus GN=Hamp PE=2 SV=1	0	50.7	0	1	0	14.5	0	0.45	0	122
HRG_MOUSE	Histidine-rich glycoprotein OS=Mus musculus GN=Hrg PE=1 SV=2	234.4	666.7	6	13	11.6	29.3	3.62	4.31	61	26
IGHG1_MOUSE	Ig gamma-1 chain C region secreted form OS=Mus musculus GN=Ighg1 PE=1 SV=1	188.5	309.2	3	6	10.2	36.1	5.6	5.06	68	49

GCAM_MOUSE	Ig gamma-2A chain C region, membrane-bound form OS=Mus musculus GN=Igh-1a PE=1 SV=3	348.7	480.1	7	9	25.4	29.9	4.07	4.33	39	36
IGG2B_MOUSE	Ig gamma-2B chain C region OS=Mus musculus GN=Igh-3 PE=1 SV=3	355.1	294.2	7	6	26.7	22.8	4.22	2.9	38	52
IGHG3_MOUSE	Ig gamma-3 chain C region OS=Mus musculus PE=1 SV=2	1162	812	18	14	56.8	47	3.19	4.88	9	20
HVM06_MOUSE	Ig heavy chain V region 102 OS=Mus musculus PE=1 SV=1	179.4	151	3	2	29.1	18.8	4.85	4.82	69	83
HVM05_MOUSE	Ig heavy chain V region 3 OS=Mus musculus GN=Ighv1-61 PE=1 SV=1	87.7	111.6	2	2	26.5	26.5	4.71	4.37	95	96
HVM60_MOUSE	Ig heavy chain V region 3-6 OS=Mus musculus GN=Ighv3-6 PE=1 SV=1	130.4	92.6	1	1	13.8	13.8	8.69	0.23	79	100
HVM54_MOUSE	Ig heavy chain V region 5-84 OS=Mus musculus PE=1 SV=1	145	148.1	3	4	35.9	36.8	6	4.58	74	84
HVM57_MOUSE	Ig heavy chain V region 6.96 OS=Mus musculus PE=4 SV=1	72.6	86.1	2	2	24.5	24.5	2.1	5.77	101	105
HVM56_MOUSE	Ig heavy chain V region 914 OS=Mus musculus PE=1 SV=1	135.3	93.7	4	3	53.6	33	3.79	2.67	77	99
HVM51_MOUSE	Ig heavy chain V region AC38 205.12 OS=Mus musculus PE=1 SV=1	427.9	289.9	5	3	59.3	33.9	2.77	4.28	30	53
HVM13_MOUSE	Ig heavy chain V region J558 OS=Mus musculus PE=1 SV=1	0	216.2	0	3	0	42.7	0	6.81	0	69
HVM21_MOUSE	Ig heavy chain V region M511 OS=Mus musculus PE=1 SV=1	478.2	437.8	7	7	68.9	71.3	2.8	2.65	29	40
HVM45_MOUSE	Ig heavy chain V region MC101 OS=Mus musculus PE=1 SV=1	0	52.2	0	2	0	33.6	0	6.33	0	120
HVM16_MOUSE	Ig heavy chain V region MOPC 21 (Fragment) OS=Mus musculus PE=1 SV=1	172.5	226.3	4	4	26.5	26.5	4.27	4.54	71	67
HVM17_MOUSE	Ig heavy chain V region MOPC 47A OS=Mus musculus PE=1 SV=1	212.9	224.2	4	4	35	35	5.38	2.21	64	68
HVM00_MOUSE	Ig heavy chain V region OS=Mus musculus PE=1 SV=1	48.8	40.5	1	1	11.4	11.4	5.89	7.51	116	124
HVM53_MOUSE	Ig heavy chain V region RF OS=Mus musculus PE=1 SV=1	126.3	117.6	3	3	23.1	23.1	4.23	6	80	93
HVM37_MOUSE	Ig heavy chain V region X44 OS=Mus musculus PE=1 SV=1	0	392.6	0	8	0	68.1	0	5.38	0	42
HVM32_MOUSE	Ig heavy chain V-III region J606 OS=Mus musculus PE=1 SV=1	0	261.3	0	6	0	49.6	0	2.63	0	61
IGKC_MOUSE	Ig kappa chain C region OS=Mus musculus PE=1 SV=1	2203	1759.2	15	16	78.3	78.3	3.85	3.75	5	6
KV2A5_MOUSE	Ig kappa chain V-II region 17S29.1 OS=Mus musculus PE=1 SV=1	0	40	0	1	0	11.5	0	3.18	0	125
KV2A7_MOUSE	Ig kappa chain V-II region 26-10 OS=Mus musculus PE=1 SV=1	371.7	299.5	5	4	46.9	43.4	3.92	2.67	36	51
KV2A6_MOUSE	Ig kappa chain V-II region 7S34.1 OS=Mus musculus PE=1 SV=1	165.5	156.7	2	2	21.2	21.2	1.96	6.81	72	79
KV3A4_MOUSE	Ig kappa chain V-III region 50S10.1 OS=Mus musculus PE=1 SV=1	243.7	228.9	3	3	40.5	40.5	3.06	1.58	57	65
KV3AM_MOUSE	Ig kappa chain V-III region PC 2154 OS=Mus musculus PE=1 SV=1	102.4	80.1	1	1	11.1	11.1	1.1	3.25	89	111
KV3A8_MOUSE	Ig kappa chain V-III region PC 3741/TEPC 111 OS=Mus musculus PE=1 SV=1	240.7	194.8	4	3	53.2	30.6	5.37	4.52	59	76
KV3AB_MOUSE	Ig kappa chain V-III region PC 4050 OS=Mus musculus PE=1 SV=1	298.7	260.3	4	3	53.2	30.6	6.12	5.39	46	62
KV3AI_MOUSE	Ig kappa chain V-III region PC 6684 OS=Mus musculus PE=1 SV=1	496.3	474	5	5	88.3	88.3	4.78	4.82	27	37
KV3AJ_MOUSE	Ig kappa chain V-III region PC 7175 OS=Mus musculus PE=1 SV=1	503.2	522.1	5	5	88.3	88.3	3.89	5.6	26	32
KV3AG_MOUSE	Ig kappa chain V-III region PC 7210 OS=Mus musculus PE=1 SV=1	0	203.8	0	2	0	30.9	0	1.51	0	75
KV4A1_MOUSE	Ig kappa chain V-IV region S107B OS=Mus musculus PE=4 SV=1	51.4	61.6	1	1	8.5	8.5	1.64	2.24	111	115
KV5AB_MOUSE	Ig kappa chain V-V region HP R16.7 OS=Mus musculus PE=1 SV=1	323.3	276.9	5	5	59.3	59.3	6.22	4.97	42	57
KV5A3_MOUSE	Ig kappa chain V-V region K2 (Fragment) OS=Mus musculus PE=1 SV=1	246.1	206.8	4	4	40.9	40.9	3.75	1.41	56	74
KV5A6_MOUSE	Ig kappa chain V-V region L6 (Fragment) OS=Mus musculus PE=4 SV=1	274.1	210.4	4	3	40.9	35.7	4.75	4.8	50	72
KV5A9_MOUSE	Ig kappa chain V-V region L7 (Fragment) OS=Mus musculus GN=Gm10881 PE=1 SV=1	102.3	65.5	2	2	18.3	18.3	2.78	1.21	90	114
KV5A4_MOUSE	Ig kappa chain V-V region MOPC 149 OS=Mus musculus PE=1 SV=1	242.8	228.8	2	2	25.9	25.9	3.16	1.28	58	66
KV5A7_MOUSE	Ig kappa chain V-V region MOPC 41 OS=Mus musculus GN=Gm5571 PE=1 SV=1	379	334.4	6	4	33.1	28.5	3.17	5.89	34	45
KV5AG_MOUSE	Ig kappa chain V-V regions OS=Mus musculus PE=1 SV=1	0	99.5	0	3	0	20.4	0	5.09	0	98
KV6A5_MOUSE	Ig kappa chain V-VI region J539 OS=Mus musculus PE=1 SV=1	116.2	84.2	2	2	24.3	24.3	2.19	4.83	82	108
KV6A7_MOUSE	Ig kappa chain V-VI region NQ2-48.2.2 OS=Mus musculus PE=2 SV=1	143.2	75.4	3	2	33.6	14	3.3	3.64	75	113
KV6AB_MOUSE	Ig kappa chain V-VI region NQ2-6.1 OS=Mus musculus PE=2 SV=1	98.4	133.4	1	1	14.8	14.8	6	4.37	92	90
KV5A1_MOUSE	Ig kappa chain V19-17 OS=Mus musculus GN=Igk-V19-17 PE=1 SV=1	226.2	178	5	4	20.8	16.1	3.92	5.33	62	77
LV1B_MOUSE	Ig lambda-1 chain V regions MOPC 104E/RPC20/J558/S104 OS=Mus musculus PE=1 SV=1	0	83.8	0	2	0	32.6	0	7.2	0	110
IGHM_MOUSE	Ig mu chain C region OS=Mus musculus GN=Ighm PE=1 SV=2	2753.4	2512.5	37	38	70	69.6	3.9	3.03	2	3
IGJ_MOUSE	Immunoglobulin J chain OS=Mus musculus GN=Jchain PE=1 SV=4	576.4	579.3	10	11	59.1	59.1	4.11	4.1	22	30
IBP2_MOUSE	Insulin-like growth factor-binding protein 2 OS=Mus musculus GN=Igfbp2 PE=2 SV=2	0	84.2	0	3	0	16.1	0	3.88	0	109
IBP4_MOUSE	Insulin-like growth factor-binding protein 4 OS=Mus musculus GN=Igfbp4 PE=1 SV=2	0	85	0	3	0	16.5	0	5.59	0	106
ITIH4_MOUSE	Inter alpha-trypsin inhibitor, heavy chain 4 OS=Mus musculus GN=Itih4 PE=1 SV=2	481.1	1445.4	9	26	12.1	45.5	4.48	4.35	28	8
KNG1_MOUSE	Kininogen-1 OS=Mus musculus GN=Kng1 PE=1 SV=1	202.7	623.2	5	11	9.4	21.3	4.67	4.83	66	27
LOXL1_MOUSE	Lysyl oxidase homolog 1 OS=Mus musculus GN=Loxl1 PE=2 SV=3	111.5	145.5	2	3	4.8	7.9	8.13	1.65	85	86
MUP1_MOUSE	Major urinary protein 1 OS=Mus musculus GN=Mup1 PE=1 SV=1	0	497.2	0	8	0	64.4	0	4.86	0	34
MUP2_MOUSE	Major urinary protein 2 OS=Mus musculus GN=Mup2 PE=1 SV=1	0	470.9	0	8	0	62.8	0	4.78	0	38

MASP1_MOUSE	Mannan-binding lectin serine protease 1 OS=Mus musculus GN=Masp1 PE=1 SV=2	275.5	214.3	7	5	14.9	8.8	4.71	3.59	49	70
MASP2_MOUSE	Mannan-binding lectin serine protease 2 OS=Mus musculus GN=Masp2 PE=1 SV=1	420.2	237.2	9	6	18.4	12.8	3.71	3.77	32	63
MBL1_MOUSE	Mannose-binding protein A OS=Mus musculus GN=Mbl1 PE=1 SV=1	301.9	146.3	4	3	25.9	13.4	3.51	3.05	44	85
MBL2_MOUSE	Mannose-binding protein C OS=Mus musculus GN=Mbl2 PE=1 SV=2	599.9	598.7	9	10	38.9	44.7	4.51	4.49	20	29
MUG1_MOUSE	Murinoglobulin-1 OS=Mus musculus GN=Mug1 PE=1 SV=3	247.3	458	5	9	4.8	10.7	4.77	5.56	55	39
IC1_MOUSE	Plasma protease C1 inhibitor OS=Mus musculus GN=Serping1 PE=1 SV=3	337.7	284.6	6	5	16.3	14.1	4.46	6.17	41	54
PLMN_MOUSE	Plasminogen OS=Mus musculus GN=Plg PE=1 SV=3	132.1	207	2	5	3.3	6.9	3.14	4.66	78	73
PZP_MOUSE	Pregnancy zone protein OS=Mus musculus GN=Pzp PE=1 SV=3	562	732.7	13	20	12.8	22	3.98	4.61	24	23
NPY_MOUSE	Pro-neuropeptide Y OS=Mus musculus GN=Npy PE=1 SV=2	70.6	76.1	1	2	13.4	21.6	9.11	3.65	103	112
PROP_MOUSE	Properdin OS=Mus musculus GN=Cfp PE=2 SV=2	308.4	278	6	6	21.8	19.6	1.96	2.47	43	56
THRB_MOUSE	Prothrombin OS=Mus musculus GN=F2 PE=1 SV=1	265.8	682.4	6	12	10.8	19.7	5.14	4.35	53	25
RET4_MOUSE	Retinol-binding protein 4 OS=Mus musculus GN=Rbp4 PE=1 SV=2	0	152.8	0	3	0	24.9	0	2.25	0	82
SEPP1_MOUSE	Selenoprotein P OS=Mus musculus GN=Sepp1 PE=1 SV=3	0	86.9	0	3	0	14.7	0	3.45	0	104
SPA3K_MOUSE	Serine protease inhibitor A3K OS=Mus musculus GN=Serpina3k PE=1 SV=2	654.8	823	13	14	39.7	41.9	3.89	4.07	19	18
TRFE_MOUSE	Serotransferrin OS=Mus musculus GN=Tf PE=1 SV=1	1108.9	1368.7	21	25	38.9	45.3	2.84	4.48	10	9
ALBU_MOUSE	Serum albumin OS=Mus musculus GN=Alb PE=1 SV=3	3017.8	3116.6	47	46	76	76.8	3.99	3.52	1	1
SAA1_MOUSE	Serum amyloid A-1 protein OS=Mus musculus GN=Saa1 PE=1 SV=2	95.1	130.1	3	2	29.5	15.6	7.03	8.47	94	91
SAMP_MOUSE	Serum amyloid P-component OS=Mus musculus GN=Apcs PE=1 SV=2	108.9	117.4	3	4	17	20.1	4.98	4.07	87	94
QSOX1_MOUSE	Sulfhydryl oxidase 1 OS=Mus musculus GN=Qsox1 PE=1 SV=1	0	163.1	0	4	0	5.6	0	3.16	0	78
SBSN_MOUSE	Suprabasin OS=Mus musculus GN=Sbsn PE=2 SV=1	0	61.5	0	2	0	4	0	3.25	0	116
TETN_MOUSE	Tetranectin OS=Mus musculus GN=Clec3b PE=1 SV=2	0	319.4	0	6	0	52	0	2.85	0	47
TSP1_MOUSE	Thrombospondin-1 OS=Mus musculus GN=Thbs1 PE=1 SV=1	2489.4	1965	45	41	45.3	43.8	4.27	3.8	4	5
TTHY_MOUSE	Transthyretin OS=Mus musculus GN=Ttr PE=1 SV=1	109.2	522.9	2	9	32	66.7	7.02	2.74	86	31
VTDB_MOUSE	Vitamin D-binding protein OS=Mus musculus GN=Gc PE=1 SV=2	216	480.2	4	10	13.9	37.4	4.48	3.96	63	35
VTNC_MOUSE	Vitronectin OS=Mus musculus GN=Vtn PE=1 SV=2	98.5	235.9	2	5	6.7	18.8	3.9	2.79	91	64

2 h Ball Milled SWCNTs

Accession	Name	Meta Score A	Meta Score B	Peptides A	Peptides B	SC [%] A	SC [%] B	RMS90 [ppm] A	RMS90 [ppm] B	Rank A	Rank B
ACTB_MOUSE	Actin, cytoplasmic 1 OS=Mus musculus GN=Actb PE=1 SV=1	51	52	1	1	4.3	4.3	8.2	3.38	105	121
A1AG1_MOUSE	Alpha-1-acid glycoprotein 1 OS=Mus musculus GN=Orm1 PE=1 SV=1	76.5	101.9	1	1	7.2	7.2	7.86	0.28	93	97
A1AT2_MOUSE	Alpha-1-antitrypsin 1-2 OS=Mus musculus GN=Serpina1b PE=1 SV=2	627.1	790.1	13	11	41.9	38	4.2	4.88	19	21
A1AT3_MOUSE	Alpha-1-antitrypsin 1-3 OS=Mus musculus GN=Serpina1c PE=1 SV=2	723	868.3	15	12	48.5	40.5	4.28	4.72	15	16
FETUA_MOUSE	Alpha-2-HS-glycoprotein OS=Mus musculus GN=Ahsg PE=1 SV=1	604.8	1281	8	15	33.3	50.7	3.88	3.32	21	11
APOA1_MOUSE	Apolipoprotein A-I OS=Mus musculus GN=Apoa1 PE=1 SV=2	1053.6	1357.8	19	22	64	69.3	3.13	4.35	10	10
APOA2_MOUSE	Apolipoprotein A-II OS=Mus musculus GN=Apoa2 PE=1 SV=2	63.4	84.9	1	2	9.8	14.7	5.3	1.47	98	107
APOA4_MOUSE	Apolipoprotein A-IV OS=Mus musculus GN=Apoa4 PE=1 SV=3	495.6	903	10	13	39.7	47.3	4.24	4.26	26	15
APOC1_MOUSE	Apolipoprotein C-I OS=Mus musculus GN=Apoc1 PE=1 SV=1	94.8	89.8	2	2	20.5	20.5	6.24	5.48	86	102
APOC2_MOUSE	Apolipoprotein C-II OS=Mus musculus GN=Apoc2 PE=2 SV=1	216.6	270.7	4	4	33	33	2.2	4.95	55	59
APOC3_MOUSE	Apolipoprotein C-III OS=Mus musculus GN=Apoc3 PE=1 SV=2	337.8	397.3	4	5	50.5	50.5	3.25	6.21	36	41
APOE_MOUSE	Apolipoprotein E OS=Mus musculus GN=Apoe PE=1 SV=2	408.2	512.3	10	10	34.1	40.8	2.93	2.59	29	33
APOH_MOUSE	Beta-2-glycoprotein 1 OS=Mus musculus GN=Apoh PE=1 SV=1	198.9	857.7	4	15	14.8	46.7	5.89	3.28	61	17
BPIA2_MOUSE	BPI fold-containing family A member 2 OS=Mus musculus GN=Bpifa2 PE=1 SV=1	45.3	0	1	0	12.8	0	5.81	0	108	0
CRP_MOUSE	C-reactive protein OS=Mus musculus GN=Crp PE=1 SV=2	303	270	7	7	36	35.1	3.82	4.72	40	60
C4BPA_MOUSE	C4b-binding protein OS=Mus musculus GN=C4bpa PE=1 SV=3	236.5	334.7	5	8	15.4	23	5.37	3.51	49	44
CD5L_MOUSE	CD5 antigen-like OS=Mus musculus GN=Cd5l PE=1 SV=3	1319.2	1245.5	22	20	58	56.5	2.69	3.44	8	12
CLUS_MOUSE	Clusterin OS=Mus musculus GN=Clu PE=1 SV=1	237.5	788.3	4	14	14.7	32.4	5.63	2.45	48	22
FA5_MOUSE	Coagulation factor V OS=Mus musculus GN=F5 PE=1 SV=1	539.2	361.6	13	7	8.2	4.1	4.87	3.59	23	43
C1QA_MOUSE	Complement C1q subcomponent subunit A OS=Mus musculus GN=C1qa PE=1 SV=2	254.3	144.5	6	2	29	11.8	4.27	7.28	45	87
C1QB_MOUSE	Complement C1q subcomponent subunit B OS=Mus musculus GN=C1qb PE=1 SV=2	370.4	211.8	5	4	21.7	16.6	4.49	2.59	32	71
C1QC_MOUSE	Complement C1q subcomponent subunit C OS=Mus musculus GN=C1qc PE=1 SV=2	227.6	122.4	4	2	20.3	11.4	1.92	3.44	52	92
C1RA_MOUSE	Complement C1r-A subcomponent OS=Mus musculus GN=C1ra PE=1 SV=1	759.4	822.7	15	16	37.9	40.7	3.93	4.53	14	19

CS1A_MOUSE	Complement C1s-A subcomponent OS=Mus musculus GN=C1sa PE=2 SV=2	648.1	614.4	12	11	25.7	25.6	5.59	5.19	16	28
CO3_MOUSE	Complement C3 OS=Mus musculus GN=C3 PE=1 SV=3	2152.1	2923.1	44	53	34.9	40	3.73	3.1	3	2
CO4B_MOUSE	Complement C4-B OS=Mus musculus GN=C4b PE=1 SV=3	1747.5	1749.4	35	37	28.4	32.2	3.53	3.5	5	7
CFAB_MOUSE	Complement factor B OS=Mus musculus GN=Cfb PE=1 SV=2	174.7	278.9	5	6	9.5	13.3	3.82	3.35	67	55
CFAH_MOUSE	Complement factor H OS=Mus musculus GN=Cfh PE=1 SV=2	1117.4	2042.6	28	39	29.5	43.7	4.31	3.71	9	4
CFAI_MOUSE	Complement factor I OS=Mus musculus GN=Cfi PE=1 SV=3	47.2	113.3	1	3	2.3	5	0.91	3.87	107	95
FETUB_MOUSE	Fetuin-B OS=Mus musculus GN=Fetub PE=1 SV=1	83.5	141.2	2	3	9.5	12.4	5.54	4.18	90	88
FIBA_MOUSE	Fibrinogen alpha chain OS=Mus musculus GN=Fga PE=1 SV=1	208.9	321.7	4	5	8.4	13.2	5.13	3.2	59	46
FIBB_MOUSE	Fibrinogen beta chain OS=Mus musculus GN=Fgb PE=1 SV=1	98.2	0	2	0	5	0	5.77	0	84	0
FIBG_MOUSE	Fibrinogen gamma chain OS=Mus musculus GN=Fgg PE=1 SV=1	194.9	302.7	6	7	20.9	24.1	3.82	4.59	63	50
FINC_MOUSE	Fibronectin OS=Mus musculus GN=Fn1 PE=1 SV=4	627.9	317.5	15	8	8.1	4	4.05	3.32	18	48
GELS_MOUSE	Gelsolin OS=Mus musculus GN=Gsn PE=1 SV=3	634.9	1100.6	16	24	32.1	42.6	2.79	4.14	17	13
HA10_MOUSE	H-2 class I histocompatibility antigen, Q10 alpha chain OS=Mus musculus GN=H2-Q10 PE=1 SV=3	139.2	725.9	4	14	15.1	44.3	2.78	4.72	71	24
HPT_MOUSE	Haptoglobin OS=Mus musculus GN=Hp PE=1 SV=1	121.3	140.3	2	4	9.2	15.9	4.36	4.73	78	89
HBA_MOUSE	Hemoglobin subunit alpha OS=Mus musculus GN=Hba PE=1 SV=2	124.2	0	4	0	22.5	0	4	0	76	0
HEMO_MOUSE	Hemopexin OS=Mus musculus GN=Hpx PE=1 SV=2	619.7	1060.2	13	22	33.9	50.9	5.63	4.1	20	14
HRG_MOUSE	Histidine-rich glycoprotein OS=Mus musculus GN=Hrg PE=1 SV=2	327.2	666.7	7	13	12.8	29.3	4.34	4.31	37	26
IGHA_MOUSE	Ig alpha chain C region OS=Mus musculus PE=1 SV=1	74.6	0	2	0	15.4	0	8.06	0	94	0
IGHG1_MOUSE	Ig gamma-1 chain C region secreted form OS=Mus musculus GN=Ighg1 PE=1 SV=1	123.1	309.2	3	6	19.4	36.1	4.14	5.06	77	49
GCAM_MOUSE	Ig gamma-2A chain C region, membrane-bound form OS=Mus musculus GN=Igh-1a PE=1 SV=3	282.9	480.1	7	9	25.4	29.9	2.29	4.33	41	36
IGG2B_MOUSE	Ig gamma-2B chain C region OS=Mus musculus GN=Igh-3 PE=1 SV=3	222	294.2	5	6	19.8	22.8	5.19	2.9	54	52
IGHG3_MOUSE	Ig gamma-3 chain C region OS=Mus musculus PE=1 SV=2	860.7	812	13	14	47	47	3.87	4.88	13	20
HVM06_MOUSE	Ig heavy chain V region 102 OS=Mus musculus PE=1 SV=1	139.2	151	3	2	29.1	18.8	4.47	4.82	70	83
HVM09_MOUSE	Ig heavy chain V region 186-1 OS=Mus musculus PE=4 SV=2	82.8	0	2	0	18.8	0	3.51	0	91	0
HVM05_MOUSE	Ig heavy chain V region 3 OS=Mus musculus GN=Ighv1-61 PE=1 SV=1	87	111.6	2	2	26.5	26.5	4.4	4.37	89	96
HVM60_MOUSE	Ig heavy chain V region 3-6 OS=Mus musculus GN=Ighv3-6 PE=1 SV=1	138.2	92.6	1	1	13.8	13.8	1.46	0.23	72	100
HVM36_MOUSE	Ig heavy chain V region 441 OS=Mus musculus PE=4 SV=1	367.2	0	8	0	72.4	0	5.5	0	33	0
HVM54_MOUSE	Ig heavy chain V region 5-84 OS=Mus musculus PE=1 SV=1	126.9	148.1	3	4	35.9	36.8	2.61	4.58	75	84
HVM57_MOUSE	Ig heavy chain V region 6.96 OS=Mus musculus PE=4 SV=1	51.6	86.1	2	2	24.5	24.5	1.98	5.77	103	105
HVM56_MOUSE	Ig heavy chain V region 914 OS=Mus musculus PE=1 SV=1	98.1	93.7	3	3	33	33	1.78	2.67	85	99
HVM51_MOUSE	Ig heavy chain V region AC38 205.12 OS=Mus musculus PE=1 SV=1	275.8	289.9	3	3	33.9	33.9	3	4.28	42	53
HVM21_MOUSE	Ig heavy chain V region M511 OS=Mus musculus PE=1 SV=1	366	437.8	6	7	67.2	71.3	2.16	2.65	34	40
HVM45_MOUSE	Ig heavy chain V region MC101 OS=Mus musculus PE=1 SV=1	70.3	52.2	2	2	33.6	33.6	3.05	6.33	95	120
HVM16_MOUSE	Ig heavy chain V region MOPC 21 (Fragment) OS=Mus musculus PE=1 SV=1	151.6	226.3	3	4	26.5	26.5	2.74	4.54	69	67
HVM17_MOUSE	Ig heavy chain V region MOPC 47A OS=Mus musculus PE=1 SV=1	200.9	224.2	4	4	35	35	2.43	2.21	60	68
HVM00_MOUSE	Ig heavy chain V region OS=Mus musculus PE=1 SV=1	51.5	40.5	1	1	11.4	11.4	7.09	7.51	104	124
HVM44_MOUSE	Ig heavy chain V region PJ14 OS=Mus musculus PE=1 SV=1	52.1	0	2	0	33.9	0	5.62	0	102	0
HVM27_MOUSE	Ig heavy chain V-III region A4 OS=Mus musculus PE=1 SV=1	131	0	3	0	26.5	0	2.19	0	74	0
IGKC_MOUSE	Ig kappa chain C region OS=Mus musculus PE=1 SV=1	1538.9	1759.2	14	16	78.3	78.3	4.26	3.75	6	6
KVM5_MOUSE	Ig kappa chain V region Mem5 (Fragment) OS=Mus musculus PE=1 SV=1	53.9	0	1	0	5.8	0	3.03	0	101	0
KV2A7_MOUSE	Ig kappa chain V-II region 26-10 OS=Mus musculus PE=1 SV=1	326	299.5	5	4	46.9	43.4	3.95	2.67	38	51
KV2A6_MOUSE	Ig kappa chain V-II region 7S34.1 OS=Mus musculus PE=1 SV=1	161.6	156.7	2	2	21.2	21.2	3.91	6.81	68	79
KV3A4_MOUSE	Ig kappa chain V-III region 50S10.1 OS=Mus musculus PE=1 SV=1	209.2	228.9	2	3	30.6	40.5	3.02	1.58	58	65
KV3AM_MOUSE	Ig kappa chain V-III region PC 2154 OS=Mus musculus PE=1 SV=1	104.1	80.1	1	1	11.1	11.1	1.24	3.25	83	111
KV3A8_MOUSE	Ig kappa chain V-III region PC 3741/TEPC 111 OS=Mus musculus PE=1 SV=1	189	194.8	3	3	30.6	30.6	3.74	4.52	64	76
KV3AI_MOUSE	Ig kappa chain V-III region PC 6684 OS=Mus musculus PE=1 SV=1	530.3	474	5	5	88.3	88.3	3.45	4.82	24	37
KV3AJ_MOUSE	Ig kappa chain V-III region PC 7175 OS=Mus musculus PE=1 SV=1	524.8	522.1	5	5	88.3	88.3	4.34	5.6	25	32
KV3AG_MOUSE	Ig kappa chain V-III region PC 7210 OS=Mus musculus PE=1 SV=1	195.1	203.8	2	2	30.9	30.9	4.57	1.51	62	75
KV4A1_MOUSE	Ig kappa chain V-IV region S107B OS=Mus musculus PE=4 SV=1	61.5	61.6	1	1	8.5	8.5	1.53	2.24	100	115
KV5AB_MOUSE	Ig kappa chain V-V region HP R16.7 OS=Mus musculus PE=1 SV=1	251.9	276.9	5	5	59.3	59.3	3.2	4.97	46	57
KV5A3_MOUSE	Ig kappa chain V-V region K2 (Fragment) OS=Mus musculus PE=1 SV=1	235.5	206.8	4	4	40.9	40.9	2.37	1.41	50	74
KV5A6_MOUSE	Ig kappa chain V-V region L6 (Fragment) OS=Mus musculus PE=4 SV=1	227.8	210.4	3	3	35.7	35.7	2.33	4.8	51	72

KV5A9_MOUSE	Ig kappa chain V-V region L7 (Fragment) OS=Mus musculus GN=Gm10881 PE=1 SV=1	108.6	65.5	2	2	23.5	18.3	6.35	1.21	81	114
KV5A4_MOUSE	Ig kappa chain V-V region MOPC 149 OS=Mus musculus PE=1 SV=1	244.4	228.8	2	2	25.9	25.9	6.82	1.28	47	66
KV5A7_MOUSE	Ig kappa chain V-V region MOPC 41 OS=Mus musculus GN=Gm5571 PE=1 SV=1	271.7	334.4	4	4	33.1	28.5	2.05	5.89	43	45
KV6A5_MOUSE	Ig kappa chain V-VI region J539 OS=Mus musculus PE=1 SV=1	104.3	84.2	2	2	24.3	24.3	4.6	4.83	82	108
KV6A7_MOUSE	Ig kappa chain V-VI region NQ2-48.2.2 OS=Mus musculus PE=2 SV=1	91.6	75.4	2	2	14	14	3.84	3.64	88	113
KV6AB_MOUSE	Ig kappa chain V-VI region NQ2-6.1 OS=Mus musculus PE=2 SV=1	81.7	133.4	1	1	14.8	14.8	6.92	4.37	92	90
KV5A1_MOUSE	Ig kappa chain V19-17 OS=Mus musculus GN=Igk-V19-17 PE=1 SV=1	212.2	178	5	4	20.8	16.1	4.05	5.33	57	77
LAC1_MOUSE	Ig lambda-1 chain C region OS=Mus musculus PE=1 SV=1	62.2	0	2	0	35.2	0	2.36	0	99	0
IGHM_MOUSE	Ig mu chain C region OS=Mus musculus GN=Ighm PE=1 SV=2	2495.3	2512.5	35	38	74.9	69.6	4.05	3.03	2	3
IGJ_MOUSE	Immunoglobulin J chain OS=Mus musculus GN=Jchain PE=1 SV=4	461.7	579.3	9	11	36.5	59.1	1.85	4.1	28	30
ITIH4_MOUSE	Inter alpha-trypsin inhibitor, heavy chain 4 OS=Mus musculus GN=Itih4 PE=1 SV=2	495.6	1445.4	12	26	15.1	45.5	3.76	4.35	27	8
K2C6A_MOUSE	Keratin, type II cytoskeletal 6A OS=Mus musculus GN=Krt6a PE=1 SV=3	68.9	0	1	0	2.2	0	0.52	0	97	0
KNG1_MOUSE	Kininogen-1 OS=Mus musculus GN=Kng1 PE=1 SV=1	177	623.2	5	11	9.5	21.3	4.31	4.83	66	27
LOXL1_MOUSE	Lysyl oxidase homolog 1 OS=Mus musculus GN=Loxl1 PE=2 SV=3	50.7	145.5	1	3	3.1	7.9	1.42	1.65	106	86
MASP1_MOUSE	Mannan-binding lectin serine protease 1 OS=Mus musculus GN=Masp1 PE=1 SV=2	181.2	214.3	4	5	7	8.8	2.6	3.59	65	70
MASP2_MOUSE	Mannan-binding lectin serine protease 2 OS=Mus musculus GN=Masp2 PE=1 SV=1	338.5	237.2	7	6	14	12.8	3.69	3.77	35	63
MBL1_MOUSE	Mannose-binding protein A OS=Mus musculus GN=Mbl1 PE=1 SV=1	216.2	146.3	4	3	25.9	13.4	3.95	3.05	56	85
MBL2_MOUSE	Mannose-binding protein C OS=Mus musculus GN=Mbl2 PE=1 SV=2	560.4	598.7	7	10	29.9	44.7	3.45	4.49	22	29
MUG1_MOUSE	Murinoglobulin-1 OS=Mus musculus GN=Mug1 PE=1 SV=3	374.4	458	6	9	5.4	10.7	3.04	5.56	31	39
IC1_MOUSE	Plasma protease C1 inhibitor OS=Mus musculus GN=Serping1 PE=1 SV=3	223.4	284.6	3	5	10.3	14.1	4.13	6.17	53	54
PLMN_MOUSE	Plasminogen OS=Mus musculus GN=Plg PE=1 SV=3	113.7	207	4	5	5.4	6.9	2.1	4.66	80	73
PLF4_MOUSE	Platelet factor 4 OS=Mus musculus GN=Pf4 PE=1 SV=1	43.7	0	1	0	11.4	0	6.74	0	109	0
PZP_MOUSE	Pregnancy zone protein OS=Mus musculus GN=Pzp PE=1 SV=3	1019.8	732.7	23	20	24.9	22	3.21	4.61	11	23
NPY_MOUSE	Pro-neuropeptide Y OS=Mus musculus GN=Npy PE=1 SV=2	69.7	76.1	2	2	13.4	21.6	1.09	3.65	96	112
PROP_MOUSE	Properdin OS=Mus musculus GN=Cfp PE=2 SV=2	382	278	7	6	24.8	19.6	2.26	2.47	30	56
THRB_MOUSE	Prothrombin OS=Mus musculus GN=F2 PE=1 SV=1	306.2	682.4	5	12	10.7	19.7	6.12	4.35	39	25
SPA3K_MOUSE	Serine protease inhibitor A3K OS=Mus musculus GN=Serpina3k PE=1 SV=2	880.7	823	14	14	40.2	41.9	3.86	4.07	12	18
TRFE_MOUSE	Serotransferrin OS=Mus musculus GN=Tf PE=1 SV=1	1403.7	1368.7	26	25	50.2	45.3	3.14	4.48	7	9
ALBU_MOUSE	Serum albumin OS=Mus musculus GN=Alb PE=1 SV=3	3476.3	3116.6	52	46	76.6	76.8	2.78	3.52	1	1
SAA1_MOUSE	Serum amyloid A-1 protein OS=Mus musculus GN=Saa1 PE=1 SV=2	42.2	130.1	1	2	8.2	15.6	0.74	8.47	110	91
SAMP_MOUSE	Serum amyloid P-component OS=Mus musculus GN=Apcs PE=1 SV=2	92	117.4	2	4	8	20.1	4.75	4.07	87	94
TSP1_MOUSE	Thrombospondin-1 OS=Mus musculus GN=Thbs1 PE=1 SV=1	2016.8	1965	39	41	41.7	43.8	3.21	3.8	4	5
TTHY_MOUSE	Transthyretin OS=Mus musculus GN=Ttr PE=1 SV=1	134.3	522.9	4	9	45.6	66.7	4.27	2.74	73	31
VTDB_MOUSE	Vitamin D-binding protein OS=Mus musculus GN=Gc PE=1 SV=2	263.9	480.2	6	10	19.3	37.4	2.34	3.96	44	35
VTNC_MOUSE	Vitronectin OS=Mus musculus GN=Vtn PE=1 SV=2	116	235.9	3	5	7.7	18.8	3.51	2.79	79	64

4h Ball Milled SWCNTs

Accession	Name	Meta Score A	Meta Score B	Peptides A	Peptides B	SC [%] A	SC [%] B	RMS90 [ppm] A	RMS90 [ppm] B	Rank A	Rank B
ACTG_MOUSE	Actin, cytoplasmic 2 OS=Mus musculus GN=Actg1 PE=1 SV=1	138.6	0	2	0	7.2	0	3.9	0	85	0
A1AT3_MOUSE	Alpha-1-antitrypsin 1-3 OS=Mus musculus GN=Serpina1c PE=1 SV=2	421.1	868.3	8	12	25.2	40.5	3.81	4.72	31	16
A1AT4_MOUSE	Alpha-1-antitrypsin 1-4 OS=Mus musculus GN=Serpina1d PE=1 SV=1	397.1	0	7	0	20.3	0	2.31	0	34	0
FETUA_MOUSE	Alpha-2-HS-glycoprotein OS=Mus musculus GN=Ahsg PE=1 SV=1	687.8	1281	10	15	35.4	50.7	2.87	3.32	17	11
ANT3_MOUSE	Antithrombin-III OS=Mus musculus GN=Serpinc1 PE=1 SV=1	71.2	92	1	2	2.4	7.1	2.74	7.03	110	101
APOA1_MOUSE	Apolipoprotein A-I OS=Mus musculus GN=Apoa1 PE=1 SV=2	958.2	1357.8	21	22	67	69.3	3.72	4.35	12	10
APOA2_MOUSE	Apolipoprotein A-II OS=Mus musculus GN=Apoa2 PE=1 SV=2	67.2	84.9	1	2	9.8	14.7	0.5	1.47	113	107
APOA4_MOUSE	Apolipoprotein A-IV OS=Mus musculus GN=Apoa4 PE=1 SV=3	530	903	11	13	42.3	47.3	4.16	4.26	26	15
APOC1_MOUSE	Apolipoprotein C-I OS=Mus musculus GN=Apoc1 PE=1 SV=1	85	89.8	2	2	20.5	20.5	3.05	5.48	101	102
APOC2_MOUSE	Apolipoprotein C-II OS=Mus musculus GN=Apoc2 PE=2 SV=1	240.7	270.7	4	4	33	33	4.06	4.95	61	59
APOC3_MOUSE	Apolipoprotein C-III OS=Mus musculus GN=Apoc3 PE=1 SV=2	336	397.3	4	5	50.5	50.5	2.62	6.21	43	41
APOE_MOUSE	Apolipoprotein E OS=Mus musculus GN=Apoe PE=1 SV=2	545.1	512.3	10	10	33.4	40.8	2.71	2.59	25	33
APOH_MOUSE	Beta-2-glycoprotein 1 OS=Mus musculus GN=Apoh PE=1 SV=1	297.9	857.7	7	15	24.3	46.7	5.42	3.28	52	17

BPIA2_MOUSE	BPI fold-containing family A member 2 OS=Mus musculus GN=Bpifa2 PE=1 SV=1	53.1	0	2	0	18.7	0	4.17	0	117	0
CRP_MOUSE	C-reactive protein OS=Mus musculus GN=Crp PE=1 SV=2	426.9	270	10	7	42.2	35.1	3.29	4.72	30	60
C4BPA_MOUSE	C4b-binding protein OS=Mus musculus GN=C4bpa PE=1 SV=3	243.3	334.7	6	8	17.3	23	3.25	3.51	60	44
EST1C_MOUSE	Carboxylesterase 1C OS=Mus musculus GN=Ces1c PE=1 SV=4	98.2	0	3	0	8.3	0	3.64	0	97	0
CD5L_MOUSE	CD5 antigen-like OS=Mus musculus GN=Cd5l PE=1 SV=3	1521.9	1245.5	22	20	57.7	56.5	3.06	3.44	7	12
CLUS_MOUSE	Clusterin OS=Mus musculus GN=Clu PE=1 SV=1	385.8	788.3	8	14	25.9	32.4	3.1	2.45	36	22
FA5_MOUSE	Coagulation factor V OS=Mus musculus GN=F5 PE=1 SV=1	578.2	361.6	11	7	6.6	4.1	5.74	3.59	23	43
C1QA_MOUSE	Complement C1q subcomponent subunit A OS=Mus musculus GN=C1qa PE=1 SV=2	223.6	144.5	4	2	24.5	11.8	3.76	7.28	64	87
C1QB_MOUSE	Complement C1q subcomponent subunit B OS=Mus musculus GN=C1qb PE=1 SV=2	346.7	211.8	5	4	21.7	16.6	3.09	2.59	42	71
C1QC_MOUSE	Complement C1q subcomponent subunit C OS=Mus musculus GN=C1qc PE=1 SV=2	246.8	122.4	4	2	20.3	11.4	5.18	3.44	56	92
C1RA_MOUSE	Complement C1r-A subcomponent OS=Mus musculus GN=C1ra PE=1 SV=1	1023.7	822.7	21	16	48.8	40.7	3.49	4.53	11	19
CS1A_MOUSE	Complement C1s-A subcomponent OS=Mus musculus GN=C1sa PE=2 SV=2	803.7	614.4	15	11	29.1	25.6	4.46	5.19	15	28
CO3_MOUSE	Complement C3 OS=Mus musculus GN=C3 PE=1 SV=3	2589	2923.1	54	53	38.8	40	3.39	3.1	3	2
CO4B_MOUSE	Complement C4-B OS=Mus musculus GN=C4b PE=1 SV=3	1799.3	1749.4	38	37	27.9	32.2	4.08	3.5	5	7
CFAB_MOUSE	Complement factor B OS=Mus musculus GN=Cfb PE=1 SV=2	198.4	278.9	5	6	10.1	13.3	3.93	3.35	73	55
CFAH_MOUSE	Complement factor H OS=Mus musculus GN=Cfh PE=1 SV=2	1283.3	2042.6	28	39	32	43.7	3.43	3.71	8	4
ECM1_MOUSE	Extracellular matrix protein 1 OS=Mus musculus GN=Ecm1 PE=1 SV=2	67.5	61.2	2	2	4.3	4.8	3.51	4.99	111	117
FETUB_MOUSE	Fetuin-B OS=Mus musculus GN=Fetub PE=1 SV=1	67.3	141.2	2	3	9.5	12.4	3.2	4.18	112	88
FIBA_MOUSE	Fibrinogen alpha chain OS=Mus musculus GN=Fga PE=1 SV=1	218.1	321.7	4	5	7.5	13.2	5.79	3.2	66	46
FIBB_MOUSE	Fibrinogen beta chain OS=Mus musculus GN=Fgb PE=1 SV=1	111.1	0	3	0	7.7	0	2.07	0	91	0
FIBG_MOUSE	Fibrinogen gamma chain OS=Mus musculus GN=Fgg PE=1 SV=1	321.7	302.7	8	7	26.6	24.1	4.7	4.59	45	50
FINC_MOUSE	Fibronectin OS=Mus musculus GN=Fn1 PE=1 SV=4	782.8	317.5	20	8	12.9	4	2.95	3.32	16	48
GELS_MOUSE	Gelsolin OS=Mus musculus GN=Gsn PE=1 SV=3	865.5	1100.6	22	24	43.2	42.6	3.32	4.14	14	13
HA10_MOUSE	H-2 class I histocompatibility antigen, Q10 alpha chain OS=Mus musculus GN=H2-Q10 PE=1 SV=3	221.3	725.9	6	14	22.2	44.3	5.4	4.72	65	24
HPT_MOUSE	Haptoglobin OS=Mus musculus GN=Hp PE=1 SV=1	41.2	140.3	1	4	3.5	15.9	0.73	4.73	121	89
HBA_MOUSE	Hemoglobin subunit alpha OS=Mus musculus GN=Hba PE=1 SV=2	244.3	0	6	0	40.8	0	3.53	0	59	0
HBB1_MOUSE	Hemoglobin subunit beta-1 OS=Mus musculus GN=Hbb-b1 PE=1 SV=2	88.9	89.5	2	3	17.7	30.6	5.89	8.11	100	103
HEMO_MOUSE	Hemopexin OS=Mus musculus GN=Hpx PE=1 SV=2	609.5	1060.2	14	22	35.4	50.9	4.11	4.1	21	14
HRG_MOUSE	Histidine-rich glycoprotein OS=Mus musculus GN=Hrg PE=1 SV=2	369.1	666.7	8	13	14.5	29.3	2.83	4.31	37	26
IGHG1_MOUSE	Ig gamma-1 chain C region secreted form OS=Mus musculus GN=Ighg1 PE=1 SV=1	185.6	309.2	4	6	22.5	36.1	6.24	5.06	77	49
GCAA_MOUSE	Ig gamma-2A chain C region, A allele OS=Mus musculus GN=Ighg PE=1 SV=1	444.4	0	10	0	31.2	0	3.86	0	29	0
IGG2B_MOUSE	Ig gamma-2B chain C region OS=Mus musculus GN=Igh-3 PE=1 SV=3	354.7	294.2	8	6	26.7	22.8	3.77	2.9	40	52
IGHG3_MOUSE	Ig gamma-3 chain C region OS=Mus musculus PE=1 SV=2	1042.2	812	18	14	58	47	3.14	4.88	10	20
HVM06_MOUSE	Ig heavy chain V region 102 OS=Mus musculus PE=1 SV=1	154.8	151	3	2	29.1	18.8	6.75	4.82	78	83
HVM09_MOUSE	Ig heavy chain V region 186-1 OS=Mus musculus PE=4 SV=2	108.2	0	2	0	18.8	0	1.29	0	94	0
HVM05_MOUSE	Ig heavy chain V region 3 OS=Mus musculus GN=Ighv1-61 PE=1 SV=1	83.3	111.6	2	2	26.5	26.5	6.99	4.37	103	96
HVM60_MOUSE	Ig heavy chain V region 3-6 OS=Mus musculus GN=Ighv3-6 PE=1 SV=1	127.4	92.6	1	1	13.8	13.8	1.53	0.23	87	100
HVM36_MOUSE	Ig heavy chain V region 441 OS=Mus musculus PE=4 SV=1	303.2	0	6	0	68.1	0	2.63	0	51	0
HVM54_MOUSE	Ig heavy chain V region 5-84 OS=Mus musculus PE=1 SV=1	152.5	148.1	4	4	36.8	36.8	2.89	4.58	80	84
HVM57_MOUSE	Ig heavy chain V region 6.96 OS=Mus musculus PE=4 SV=1	79	86.1	2	2	24.5	24.5	3.87	5.77	105	105
HVM56_MOUSE	Ig heavy chain V region 914 OS=Mus musculus PE=1 SV=1	123.1	93.7	3	3	33	33	6.14	2.67	89	99
HVM51_MOUSE	Ig heavy chain V region AC38 205.12 OS=Mus musculus PE=1 SV=1	306.8	289.9	4	3	42.4	33.9	5.8	4.28	50	53
HVM21_MOUSE	Ig heavy chain V region M511 OS=Mus musculus PE=1 SV=1	481.5	437.8	7	7	68.9	71.3	3.96	2.65	28	40
HVM45_MOUSE	Ig heavy chain V region MC101 OS=Mus musculus PE=1 SV=1	93	52.2	2	2	33.6	33.6	6.09	6.33	99	120
HVM16_MOUSE	Ig heavy chain V region MOPC 21 (Fragment) OS=Mus musculus PE=1 SV=1	224.1	226.3	5	4	26.5	26.5	5.18	4.54	63	67
HVM17_MOUSE	Ig heavy chain V region MOPC 47A OS=Mus musculus PE=1 SV=1	229.7	224.2	4	4	35	35	7.22	2.21	62	68
HVM00_MOUSE	Ig heavy chain V region OS=Mus musculus PE=1 SV=1	66.3	40.5	1	1	11.4	11.4	7.59	7.51	114	124
HVM44_MOUSE	Ig heavy chain V region PJ14 OS=Mus musculus PE=1 SV=1	46	0	1	0	5.2	0	3.07	0	119	0
HVM53_MOUSE	Ig heavy chain V region RF OS=Mus musculus PE=1 SV=1	97.9	117.6	2	3	13.7	23.1	3.89	6	98	93
HVM32_MOUSE	Ig heavy chain V-III region J606 OS=Mus musculus PE=1 SV=1	245.4	261.3	6	6	38.3	49.6	2.78	2.63	57	61
IGKC_MOUSE	Ig kappa chain C region OS=Mus musculus PE=1 SV=1	1620.4	1759.2	17	16	88.7	78.3	3.5	3.75	6	6
KV2A5_MOUSE	Ig kappa chain V-II region 17S29.1 OS=Mus musculus PE=1 SV=1	78.7	40	1	1	11.5	11.5	8.38	3.18	106	125

KV2A7_MOUSE	Ig kappa chain V-II region 26-10 OS=Mus musculus PE=1 SV=1	352.8	299.5	5	4	46.9	43.4	2.53	2.67	41	51
KV2A6_MOUSE	Ig kappa chain V-II region 7S34.1 OS=Mus musculus PE=1 SV=1	216.4	156.7	3	2	21.2	21.2	3.17	6.81	67	79
KV3A4_MOUSE	Ig kappa chain V-III region 50S10.1 OS=Mus musculus PE=1 SV=1	213.8	228.9	3	3	40.5	40.5	1.83	1.58	68	65
KV3AM_MOUSE	Ig kappa chain V-III region PC 2154 OS=Mus musculus PE=1 SV=1	79.1	80.1	1	1	11.1	11.1	0.26	3.25	104	111
KV3A5_MOUSE	Ig kappa chain V-III region PC 2413 OS=Mus musculus PE=1 SV=1	149.2	0	2	0	24.3	0	0.94	0	81	0
KV3A8_MOUSE	Ig kappa chain V-III region PC 3741/TEPC 111 OS=Mus musculus PE=1 SV=1	211.2	194.8	4	3	53.2	30.6	2.32	4.52	70	76
KV3AB_MOUSE	Ig kappa chain V-III region PC 4050 OS=Mus musculus PE=1 SV=1	308.7	260.3	4	3	53.2	30.6	3.13	5.39	48	62
KV3AI_MOUSE	Ig kappa chain V-III region PC 6684 OS=Mus musculus PE=1 SV=1	520.1	474	6	5	88.3	88.3	4.77	4.82	27	37
KV3AJ_MOUSE	Ig kappa chain V-III region PC 7175 OS=Mus musculus PE=1 SV=1	562.2	522.1	6	5	88.3	88.3	3.55	5.6	24	32
KV3AE_MOUSE	Ig kappa chain V-III region PC 7183 OS=Mus musculus PE=1 SV=1	266.5	0	3	0	53.2	0	3.2	0	54	0
KV5AF_MOUSE	Ig kappa chain V-V region HP 91A3 OS=Mus musculus PE=1 SV=1	148.6	0	4	0	39.8	0	6.25	0	82	0
KV5AC_MOUSE	Ig kappa chain V-V region HP 93G7 OS=Mus musculus PE=1 SV=1	310.1	0	6	0	59.3	0	3.29	0	47	0
KV5A3_MOUSE	Ig kappa chain V-V region K2 (Fragment) OS=Mus musculus PE=1 SV=1	307	206.8	4	4	40.9	40.9	4.5	1.41	49	74
KV5A6_MOUSE	Ig kappa chain V-V region L6 (Fragment) OS=Mus musculus PE=4 SV=1	389.5	210.4	7	3	40	35.7	3.88	4.8	35	72
KV5A9_MOUSE	Ig kappa chain V-V region L7 (Fragment) OS=Mus musculus GN=Gm10881 PE=1 SV=1	121.1	65.5	2	2	23.5	18.3	4.46	1.21	90	114
KV5A4_MOUSE	Ig kappa chain V-V region MOPC 149 OS=Mus musculus PE=1 SV=1	245.2	228.8	2	2	25.9	25.9	5.32	1.28	58	66
KV5AA_MOUSE	Ig kappa chain V-V region MOPC 173 OS=Mus musculus PE=1 SV=1	212.6	0	4	0	51.9	0	4.5	0	69	0
KV5A7_MOUSE	Ig kappa chain V-V region MOPC 41 OS=Mus musculus GN=Gm5571 PE=1 SV=1	399.9	334.4	5	4	33.1	28.5	3.2	5.89	33	45
KV6A5_MOUSE	Ig kappa chain V-VI region J539 OS=Mus musculus PE=1 SV=1	153.5	84.2	2	2	24.3	24.3	3.33	4.83	79	108
KV6AB_MOUSE	Ig kappa chain V-VI region NQ2-6.1 OS=Mus musculus PE=2 SV=1	101.9	133.4	1	1	14.8	14.8	5.19	4.37	96	90
KV6AA_MOUSE	Ig kappa chain V-VI region NQ5-78.2.6 OS=Mus musculus PE=2 SV=1	147.1	0	3	0	32.7	0	2.39	0	83	0
KV5A1_MOUSE	Ig kappa chain V19-17 OS=Mus musculus GN=Igk-V19-17 PE=1 SV=1	188.6	178	5	4	20.8	16.1	4.3	5.33	75	77
LAC1_MOUSE	Ig lambda-1 chain C region OS=Mus musculus PE=1 SV=1	64.9	0	2	0	27.6	0	5.22	0	115	0
LV1A_MOUSE	Ig lambda-1 chain V region OS=Mus musculus PE=1 SV=2	76	0	1	0	7.7	0	3.06	0	107	0
LAC2_MOUSE	Ig lambda-2 chain C region OS=Mus musculus GN=Iglc2 PE=1 SV=1	73.6	0	2	0	34.6	0	2.91	0	109	0
IGHM_MOUSE	Ig mu chain C region OS=Mus musculus GN=Ighm PE=1 SV=2	2907.3	2512.5	41	38	79.5	69.6	3.22	3.03	2	3
IGJ_MOUSE	Immunoglobulin J chain OS=Mus musculus GN=Jchain PE=1 SV=4	595.1	579.3	11	11	59.1	59.1	4.17	4.1	22	30
ITIH4_MOUSE	Inter alpha-trypsin inhibitor, heavy chain 4 OS=Mus musculus GN=Itih4 PE=1 SV=2	613.4	1445.4	12	26	15.4	45.5	2.66	4.35	19	8
K1C10_MOUSE	Keratin, type I cytoskeletal 10 OS=Mus musculus GN=Krt10 PE=1 SV=3	188.8	0	5	0	9.3	0	3.59	0	74	0
K1C13_MOUSE	Keratin, type I cytoskeletal 13 OS=Mus musculus GN=Krt13 PE=1 SV=2	135	0	3	0	5.9	0	5.34	0	86	0
K22E_MOUSE	Keratin, type II cytoskeletal 2 epidermal OS=Mus musculus GN=Krt2 PE=1 SV=1	74.6	0	2	0	3.3	0	5.87	0	108	0
K2C74_MOUSE	Keratin, type II cytoskeletal 74 OS=Mus musculus GN=Krt74 PE=3 SV=1	83.7	0	2	0	4.2	0	5.56	0	102	0
KNG1_MOUSE	Kininogen-1 OS=Mus musculus GN=Kng1 PE=1 SV=1	207.1	623.2	4	11	6.2	21.3	3.85	4.83	72	27
LOXL1_MOUSE	Lysyl oxidase homolog 1 OS=Mus musculus GN=Loxl1 PE=2 SV=3	47.7	145.5	1	3	3.1	7.9	0.12	1.65	118	86
MASP1_MOUSE	Mannan-binding lectin serine protease 1 OS=Mus musculus GN=Masp1 PE=1 SV=2	328.7	214.3	7	5	12.9	8.8	1.4	3.59	44	70
MASP2_MOUSE	Mannan-binding lectin serine protease 2 OS=Mus musculus GN=Masp2 PE=1 SV=1	368	237.2	8	6	14.6	12.8	4.93	3.77	38	63
MBL1_MOUSE	Mannose-binding protein A OS=Mus musculus GN=Mbl1 PE=1 SV=1	291.2	146.3	6	3	28.5	13.4	4.45	3.05	53	85
MBL2_MOUSE	Mannose-binding protein C OS=Mus musculus GN=Mbl2 PE=1 SV=2	650	598.7	10	10	43	44.7	3.39	4.49	18	29
MUG1_MOUSE	Murinoglobulin-1 OS=Mus musculus GN=Mug1 PE=1 SV=3	188	458	5	9	4.9	10.7	4.75	5.56	76	39
IC1_MOUSE	Plasma protease C1 inhibitor OS=Mus musculus GN=Serping1 PE=1 SV=3	317.1	284.6	5	5	14.1	14.1	4.53	6.17	46	54
PLMN_MOUSE	Plasminogen OS=Mus musculus GN=Plg PE=1 SV=3	210.2	207	6	5	7.5	6.9	3.23	4.66	71	73
PLF4_MOUSE	Platelet factor 4 OS=Mus musculus GN=Pf4 PE=1 SV=1	45.5	0	1	0	11.4	0	1.97	0	120	0
PZP_MOUSE	Pregnancy zone protein OS=Mus musculus GN=Pzp PE=1 SV=3	609.8	732.7	16	20	15	22	3.59	4.61	20	23
NPY_MOUSE	Pro-neuropeptide Y OS=Mus musculus GN=Npy PE=1 SV=2	104.8	76.1	2	2	13.4	21.6	7.17	3.65	95	112
PROP_MOUSE	Properdin OS=Mus musculus GN=Cfp PE=2 SV=2	358.2	278	8	6	27.2	19.6	3.67	2.47	39	56
THRB_MOUSE	Prothrombin OS=Mus musculus GN=F2 PE=1 SV=1	413.7	682.4	8	12	13.8	19.7	3.64	4.35	32	25
SPA3K_MOUSE	Serine protease inhibitor A3K OS=Mus musculus GN=Serpina3k PE=1 SV=2	875.3	823	17	14	43.8	41.9	3.25	4.07	13	18
TRFE_MOUSE	Serotransferrin OS=Mus musculus GN=Tf PE=1 SV=1	1110.6	1368.7	24	25	43.9	45.3	3.56	4.48	9	9
ALBU_MOUSE	Serum albumin OS=Mus musculus GN=Alb PE=1 SV=3	3122.8	3116.6	47	46	79.4	76.8	3.49	3.52	1	1
SAA1_MOUSE	Serum amyloid A-1 protein OS=Mus musculus GN=Saa1 PE=1 SV=2	110.7	130.1	3	2	26.2	15.6	6.05	8.47	92	91
SAA2_MOUSE	Serum amyloid A-2 protein OS=Mus musculus GN=Saa2 PE=1 SV=1	62.2	0	2	0	15.6	0	3.19	0	116	0
SAMP_MOUSE	Serum amyloid P-component OS=Mus musculus GN=Apcs PE=1 SV=2	108.3	117.4	3	4	17	20.1	1.42	4.07	93	94

TSP1_MOUSE	Thrombospondin-1 OS=Mus musculus GN=Thbs1 PE=1 SV=1	2325	1965	43	41	43.7	43.8	3.65	3.8	4	5
TTHY_MOUSE	Transthyretin OS=Mus musculus GN=Ttr PE=1 SV=1	146.2	522.9	3	9	40.8	66.7	4.16	2.74	84	31
VTDB_MOUSE	Vitamin D-binding protein OS=Mus musculus GN=Gc PE=1 SV=2	251.4	480.2	6	10	17.9	37.4	3.08	3.96	55	35
VTNC_MOUSE	Vitronectin OS=Mus musculus GN=Vtn PE=1 SV=2	125.6	235.9	3	5	9	18.8	1.23	2.79	88	64

6h Ball Milled SWCNTs

Accession	Name	Meta Score A	Meta Score B	Peptides A	Peptides B	SC [%] A	SC [%] B	RMS90 [ppm] A	RMS90 [ppm] B	Rank A	Rank B
ACTB_MOUSE	Actin, cytoplasmic 1 OS=Mus musculus GN=Actb PE=1 SV=1	76.8	52	2	1	7.2	4.3	4.61	3.38	85	121
A1AT3_MOUSE	Alpha-1-antitrypsin 1-3 OS=Mus musculus GN=Serpina1c PE=1 SV=2	362.2	868.3	7	12	24.5	40.5	3.24	4.72	26	16
FETUA_MOUSE	Alpha-2-HS-glycoprotein OS=Mus musculus GN=Ahsg PE=1 SV=1	533.6	1281	8	15	33.3	50.7	2.73	3.32	18	11
ANT3_MOUSE	Antithrombin-III OS=Mus musculus GN=Serpinc1 PE=1 SV=1	71.2	92	1	2	2.4	7.1	8.33	7.03	88	101
APOA1_MOUSE	Apolipoprotein A-I OS=Mus musculus GN=Apoa1 PE=1 SV=2	833.1	1357.8	17	22	65.5	69.3	3.95	4.35	11	10
APOA2_MOUSE	Apolipoprotein A-II OS=Mus musculus GN=Apoa2 PE=1 SV=2	45.6	84.9	1	2	9.8	14.7	3.48	1.47	103	107
APOA4_MOUSE	Apolipoprotein A-IV OS=Mus musculus GN=Apoa4 PE=1 SV=3	439.9	903	9	13	37	47.3	1.69	4.26	21	15
APOC1_MOUSE	Apolipoprotein C-I OS=Mus musculus GN=Apoc1 PE=1 SV=1	89.7	89.8	2	2	20.5	20.5	2.67	5.48	79	102
APOC2_MOUSE	Apolipoprotein C-II OS=Mus musculus GN=Apoc2 PE=2 SV=1	177.2	270.7	3	4	30.9	33	0.92	4.95	61	59
APOC3_MOUSE	Apolipoprotein C-III OS=Mus musculus GN=Apoc3 PE=1 SV=2	312.1	397.3	4	5	50.5	50.5	4.21	6.21	35	41
APOE_MOUSE	Apolipoprotein E OS=Mus musculus GN=Apoe PE=1 SV=2	325.3	512.3	9	10	29.6	40.8	3.35	2.59	32	33
APOH_MOUSE	Beta-2-glycoprotein 1 OS=Mus musculus GN=Apoh PE=1 SV=1	89.5	857.7	2	15	8.7	46.7	8.24	3.28	80	17
CRP_MOUSE	C-reactive protein OS=Mus musculus GN=Crp PE=1 SV=2	323	270	8	7	36.4	35.1	4.98	4.72	33	60
C4BPA_MOUSE	C4b-binding protein OS=Mus musculus GN=C4bpa PE=1 SV=3	187.3	334.7	5	8	14.3	23	3.87	3.51	60	44
CD5L_MOUSE	CD5 antigen-like OS=Mus musculus GN=Cd5l PE=1 SV=3	1245.1	1245.5	20	20	56.5	56.5	3.06	3.44	6	12
CLUS_MOUSE	Clusterin OS=Mus musculus GN=Clu PE=1 SV=1	256.1	788.3	7	14	21.9	32.4	4.07	2.45	42	22
FA5_MOUSE	Coagulation factor V OS=Mus musculus GN=F5 PE=1 SV=1	555.3	361.6	11	7	6.5	4.1	5.59	3.59	17	43
C1QA_MOUSE	Complement C1q subcomponent subunit A OS=Mus musculus GN=C1qa PE=1 SV=2	305.2	144.5	5	2	29	11.8	3.97	7.28	36	87
C1QB_MOUSE	Complement C1q subcomponent subunit B OS=Mus musculus GN=C1qb PE=1 SV=2	354.2	211.8	5	4	21.7	16.6	2.98	2.59	27	71
C1QC_MOUSE	Complement C1q subcomponent subunit C OS=Mus musculus GN=C1qc PE=1 SV=2	227.1	122.4	4	2	20.3	11.4	3.08	3.44	51	92
C1RA_MOUSE	Complement C1r-A subcomponent OS=Mus musculus GN=C1ra PE=1 SV=1	739.9	822.7	14	16	34.4	40.7	3.78	4.53	13	19
CS1A_MOUSE	Complement C1s-A subcomponent OS=Mus musculus GN=C1sa PE=2 SV=2	802.6	614.4	14	11	28.2	25.6	3.81	5.19	12	28
CO3_MOUSE	Complement C3 OS=Mus musculus GN=C3 PE=1 SV=3	2095.7	2923.1	47	53	34.5	40	3.81	3.1	3	2
CO4B_MOUSE	Complement C4-B OS=Mus musculus GN=C4b PE=1 SV=3	1676.1	1749.4	30	37	26.7	32.2	4.93	3.5	5	7
CFAB_MOUSE	Complement factor B OS=Mus musculus GN=Cfb PE=1 SV=2	84.1	278.9	3	6	6.2	13.3	7.01	3.35	82	55
CFAH_MOUSE	Complement factor H OS=Mus musculus GN=Cfh PE=1 SV=2	1065.9	2042.6	23	39	23.9	43.7	3.46	3.71	8	4
FIBA_MOUSE	Fibrinogen alpha chain OS=Mus musculus GN=Fga PE=1 SV=1	164.9	321.7	4	5	7.5	13.2	3.22	3.2	64	46
FIBB_MOUSE	Fibrinogen beta chain OS=Mus musculus GN=Fgb PE=1 SV=1	67.7	0	2	0	5	0	7.01	0	89	0
FIBG_MOUSE	Fibrinogen gamma chain OS=Mus musculus GN=Fgg PE=1 SV=1	224.8	302.7	5	7	17.9	24.1	5.75	4.59	52	50
FINC_MOUSE	Fibronectin OS=Mus musculus GN=Fn1 PE=1 SV=4	563.2	317.5	13	8	7.5	4	3.24	3.32	16	48
GELS_MOUSE	Gelsolin OS=Mus musculus GN=Gsn PE=1 SV=3	636.9	1100.6	16	24	32.6	42.6	3.89	4.14	14	13
HA10_MOUSE	H-2 class I histocompatibility antigen, Q10 alpha chain OS=Mus musculus GN=H2-Q10 PE=1 SV=3	201.2	725.9	4	14	14.5	44.3	3.34	4.72	58	24
HBB1_MOUSE	Hemoglobin subunit beta-1 OS=Mus musculus GN=Hbb-b1 PE=1 SV=2	71.8	89.5	2	3	17.7	30.6	5.13	8.11	87	103
HEMO_MOUSE	Hemopexin OS=Mus musculus GN=Hpx PE=1 SV=2	388.2	1060.2	10	22	25.9	50.9	3.4	4.1	25	14
HRG_MOUSE	Histidine-rich glycoprotein OS=Mus musculus GN=Hrg PE=1 SV=2	305	666.7	8	13	16.2	29.3	4.61	4.31	37	26
IGHA_MOUSE	Ig alpha chain C region OS=Mus musculus PE=1 SV=1	80.5	0	2	0	15.4	0	6.52	0	83	0
IGHG1_MOUSE	Ig gamma-1 chain C region secreted form OS=Mus musculus GN=Ighg1 PE=1 SV=1	43.1	309.2	1	6	3.7	36.1	6.39	5.06	105	49
GCAA_MOUSE	Ig gamma-2A chain C region, A allele OS=Mus musculus GN=Ighg PE=1 SV=1	213.5	0	6	0	25.8	0	3.79	0	56	0
IGG2B_MOUSE	Ig gamma-2B chain C region OS=Mus musculus GN=Igh-3 PE=1 SV=3	317.3	294.2	8	6	26.7	22.8	4.04	2.9	34	52
IGHG3_MOUSE	Ig gamma-3 chain C region OS=Mus musculus PE=1 SV=2	1013.6	812	16	14	56.8	47	3.34	4.88	10	20
HVM06_MOUSE	Ig heavy chain V region 102 OS=Mus musculus PE=1 SV=1	170.3	151	3	2	29.1	18.8	3.83	4.82	63	83
HVM09_MOUSE	Ig heavy chain V region 186-1 OS=Mus musculus PE=4 SV=2	123.8	0	2	0	18.8	0	5.87	0	71	0
HVM05_MOUSE	Ig heavy chain V region 3 OS=Mus musculus GN=Ighv1-61 PE=1 SV=1	97	111.6	2	2	26.5	26.5	6.07	4.37	77	96
HVM60_MOUSE	Ig heavy chain V region 3-6 OS=Mus musculus GN=Ighv3-6 PE=1 SV=1	125.6	92.6	1	1	13.8	13.8	3.34	0.23	69	100

HVM36_MOUSE	Ig heavy chain V region 441 OS=Mus musculus PE=4 SV=1	335	0	8	0	72.4	0	3.7	0	31	0
HVM54_MOUSE	Ig heavy chain V region 5-84 OS=Mus musculus PE=1 SV=1	153.6	148.1	3	4	35.9	36.8	4.33	4.58	66	84
HVM57_MOUSE	Ig heavy chain V region 6.96 OS=Mus musculus PE=4 SV=1	65.9	86.1	2	2	24.5	24.5	4.19	5.77	91	105
HVM56_MOUSE	Ig heavy chain V region 914 OS=Mus musculus PE=1 SV=1	64.4	93.7	2	3	16.5	33	4.96	2.67	93	99
HVM51_MOUSE	Ig heavy chain V region AC38 205.12 OS=Mus musculus PE=1 SV=1	233.1	289.9	3	3	33.9	33.9	1.95	4.28	48	53
HVM45_MOUSE	Ig heavy chain V region MC101 OS=Mus musculus PE=1 SV=1	63.9	52.2	2	2	28.4	33.6	6.42	6.33	95	120
HVM16_MOUSE	Ig heavy chain V region MOPC 21 (Fragment) OS=Mus musculus PE=1 SV=1	174.8	226.3	4	4	26.5	26.5	5.38	4.54	62	67
HVM17_MOUSE	Ig heavy chain V region MOPC 47A OS=Mus musculus PE=1 SV=1	209.9	224.2	4	4	35	35	5.97	2.21	57	68
HVM00_MOUSE	Ig heavy chain V region OS=Mus musculus PE=1 SV=1	66	40.5	1	1	11.4	11.4	8.98	7.51	90	124
HVM18_MOUSE	Ig heavy chain V regions TEPC 15/S107/HPCM1/HPCM2/HPCM3 OS=Mus musculus PE=1 SV=1	340.1	0	6	0	66.7	0	1.33	0	29	0
HVM35_MOUSE	Ig heavy chain V-III region HPC76 (Fragment) OS=Mus musculus PE=4 SV=1	124.6	0	3	0	22.5	0	6.61	0	70	0
IGKC_MOUSE	Ig kappa chain C region OS=Mus musculus PE=1 SV=1	1169.7	1759.2	14	16	78.3	78.3	3.9	3.75	7	6
KVM5_MOUSE	Ig kappa chain V region Mem5 (Fragment) OS=Mus musculus PE=1 SV=1	43.2	0	1	0	5.8	0	1.98	0	104	0
KV2A7_MOUSE	Ig kappa chain V-II region 26-10 OS=Mus musculus PE=1 SV=1	282.5	299.5	5	4	46.9	43.4	1.45	2.67	41	51
KV2A6_MOUSE	Ig kappa chain V-II region 7S34.1 OS=Mus musculus PE=1 SV=1	156.1	156.7	2	2	21.2	21.2	4.6	6.81	65	79
KV3A4_MOUSE	Ig kappa chain V-III region 50S10.1 OS=Mus musculus PE=1 SV=1	250.5	228.9	3	3	40.5	40.5	4.35	1.58	43	65
KV3AM_MOUSE	Ig kappa chain V-III region PC 2154 OS=Mus musculus PE=1 SV=1	87.7	80.1	1	1	11.1	11.1	3.92	3.25	81	111
KV3A8_MOUSE	Ig kappa chain V-III region PC 3741/TEPC 111 OS=Mus musculus PE=1 SV=1	217.2	194.8	3	3	30.6	30.6	5.95	4.52	55	76
KV3AB_MOUSE	Ig kappa chain V-III region PC 4050 OS=Mus musculus PE=1 SV=1	289.5	260.3	3	3	30.6	30.6	5.91	5.39	38	62
KV3AI_MOUSE	Ig kappa chain V-III region PC 6684 OS=Mus musculus PE=1 SV=1	431.8	474	5	5	88.3	88.3	4.32	4.82	22	37
KV3AJ_MOUSE	Ig kappa chain V-III region PC 7175 OS=Mus musculus PE=1 SV=1	407.3	522.1	5	5	88.3	88.3	5.02	5.6	23	32
KV3AG_MOUSE	Ig kappa chain V-III region PC 7210 OS=Mus musculus PE=1 SV=1	196.5	203.8	2	2	30.9	30.9	6.24	1.51	59	75
KV4A1_MOUSE	Ig kappa chain V-IV region S107B OS=Mus musculus PE=4 SV=1	47.6	61.6	1	1	8.5	8.5	0.53	2.24	101	115
KV5AB_MOUSE	Ig kappa chain V-V region HP R16.7 OS=Mus musculus PE=1 SV=1	287.3	276.9	5	5	59.3	59.3	4.78	4.97	39	57
KV5A3_MOUSE	Ig kappa chain V-V region K2 (Fragment) OS=Mus musculus PE=1 SV=1	243.8	206.8	4	4	40.9	40.9	3.5	1.41	46	74
KV5A6_MOUSE	Ig kappa chain V-V region L6 (Fragment) OS=Mus musculus PE=4 SV=1	246.8	210.4	4	3	40	35.7	2.92	4.8	45	72
KV5A9_MOUSE	Ig kappa chain V-V region L7 (Fragment) OS=Mus musculus GN=Gm10881 PE=1 SV=1	72.6	65.5	1	2	13	18.3	5.58	1.21	86	114
KV5A4_MOUSE	Ig kappa chain V-V region MOPC 149 OS=Mus musculus PE=1 SV=1	230.5	228.8	2	2	25.9	25.9	7.09	1.28	49	66
KV5A7_MOUSE	Ig kappa chain V-V region MOPC 41 OS=Mus musculus GN=Gm5571 PE=1 SV=1	229.9	334.4	3	4	28.5	28.5	2.97	5.89	50	45
KV6A5_MOUSE	Ig kappa chain V-VI region J539 OS=Mus musculus PE=1 SV=1	123.7	84.2	2	2	24.3	24.3	3.21	4.83	72	108
KV6A6_MOUSE	Ig kappa chain V-VI region NQ2-17.4.1 OS=Mus musculus PE=2 SV=1	100.1	0	2	0	14	0	0.93	0	76	0
KV6AB_MOUSE	Ig kappa chain V-VI region NQ2-6.1 OS=Mus musculus PE=2 SV=1	104.7	133.4	1	1	14.8	14.8	5.81	4.37	74	90
KV5A1_MOUSE	Ig kappa chain V19-17 OS=Mus musculus GN=Igk-V19-17 PE=1 SV=1	217.4	178	4	4	16.1	16.1	2.82	5.33	54	77
IGHM_MOUSE	Ig mu chain C region OS=Mus musculus GN=Ighm PE=1 SV=2	2606.4	2512.5	39	38	70	69.6	3.22	3.03	2	3
IGJ_MOUSE	Immunoglobulin J chain OS=Mus musculus GN=Jchain PE=1 SV=4	464.1	579.3	9	11	58.5	59.1	2.17	4.1	20	30
ITIH4_MOUSE	Inter alpha-trypsin inhibitor, heavy chain 4 OS=Mus musculus GN=Itih4 PE=1 SV=2	346.9	1445.4	8	26	10.5	45.5	3.92	4.35	28	8
K22E_MOUSE	Keratin, type II cytoskeletal 2 epidermal OS=Mus musculus GN=Krt2 PE=1 SV=1	47.5	0	1	0	2	0	6.8	0	102	0
K2C74_MOUSE	Keratin, type II cytoskeletal 74 OS=Mus musculus GN=Krt74 PE=3 SV=1	52.7	0	1	0	2.4	0	1.89	0	99	0
KNG1_MOUSE	Kininogen-1 OS=Mus musculus GN=Kng1 PE=1 SV=1	103.2	623.2	3	11	5	21.3	2.67	4.83	75	27
LOXL1_MOUSE	Lysyl oxidase homolog 1 OS=Mus musculus GN=Loxl1 PE=2 SV=3	50.8	145.5	1	3	3.1	7.9	0.51	1.65	100	86
MASP1_MOUSE	Mannan-binding lectin serine protease 1 OS=Mus musculus GN=Masp1 PE=1 SV=2	107.9	214.3	3	5	5.4	8.8	3.67	3.59	73	70
MASP2_MOUSE	Mannan-binding lectin serine protease 2 OS=Mus musculus GN=Masp2 PE=1 SV=1	336.6	237.2	7	6	14.5	12.8	4.02	3.77	30	63
MBL1_MOUSE	Mannose-binding protein A OS=Mus musculus GN=Mbl1 PE=1 SV=1	221.8	146.3	4	3	25.9	13.4	2.6	3.05	53	85
MBL2_MOUSE	Mannose-binding protein C OS=Mus musculus GN=Mbl2 PE=1 SV=2	586	598.7	10	10	48	44.7	3.32	4.49	15	29
MUG1_MOUSE	Murinoglobulin-1 OS=Mus musculus GN=Mug1 PE=1 SV=3	136.6	458	3	9	2.4	10.7	4.92	5.56	68	39
IC1_MOUSE	Plasma protease C1 inhibitor OS=Mus musculus GN=Serping1 PE=1 SV=3	283.6	284.6	4	5	12.5	14.1	5.91	6.17	40	54
PLMN_MOUSE	Plasminogen OS=Mus musculus GN=Plg PE=1 SV=3	79.9	207	2	5	2.1	6.9	2.37	4.66	84	73
PLF4_MOUSE	Platelet factor 4 OS=Mus musculus GN=Pf4 PE=1 SV=1	64	0	2	0	20	0	3.64	0	94	0
PZP_MOUSE	Pregnancy zone protein OS=Mus musculus GN=Pzp PE=1 SV=3	404	732.7	11	20	10	22	4.35	4.61	24	23
PROP_MOUSE	Properdin OS=Mus musculus GN=Cfp PE=2 SV=2	249.3	278	5	6	16.6	19.6	4.19	2.47	44	56
THRB_MOUSE	Prothrombin OS=Mus musculus GN=F2 PE=1 SV=1	242.4	682.4	4	12	7.1	19.7	4.58	4.35	47	25
SPA3K_MOUSE	Serine protease inhibitor A3K OS=Mus musculus GN=Serpina3k PE=1 SV=2	481.9	823	13	14	41.6	41.9	3.97	4.07	19	18

TRFE_MOUSE	Serotransferrin OS=Mus musculus GN=Tf PE=1 SV=1	1039.9	1368.7	21	25	40.5	45.3	1.57	4.48	9	9
ALBU_MOUSE	Serum albumin OS=Mus musculus GN=Alb PE=1 SV=3	2611.9	3116.6	41	46	69.2	76.8	3.33	3.52	1	1
SAA1_MOUSE	Serum amyloid A-1 protein OS=Mus musculus GN=Saa1 PE=1 SV=2	56.5	130.1	2	2	15.6	15.6	4.23	8.47	97	91
SAMP_MOUSE	Serum amyloid P-component OS=Mus musculus GN=Apcs PE=1 SV=2	65.1	117.4	1	4	5.4	20.1	1.71	4.07	92	94
SPTB1_MOUSE	Spectrin beta chain, erythrocytic OS=Mus musculus GN=Sptb PE=1 SV=4	55.8	0	1	0	0.5	0	4.96	0	98	0
TSP1_MOUSE	Thrombospondin-1 OS=Mus musculus GN=Thbs1 PE=1 SV=1	2027.3	1965	37	41	41.6	43.8	3.37	3.8	4	5
TTHY_MOUSE	Transthyretin OS=Mus musculus GN=Ttr PE=1 SV=1	95.3	522.9	2	9	32	66.7	3.93	2.74	78	31
VTDB_MOUSE	Vitamin D-binding protein OS=Mus musculus GN=Gc PE=1 SV=2	150.8	480.2	3	10	10.7	37.4	3.61	3.96	67	35
VTNC_MOUSE	Vitronectin OS=Mus musculus GN=Vtn PE=1 SV=2	58.3	235.9	1	5	3.8	18.8	9.71	2.79	96	64

8h Ball Milled SWCNTs

Accession	Name	Meta Score A	Meta Score B	Peptides A	Peptides B	SC [%] A	SC [%] B	RMS90 [ppm] A	RMS90 [ppm] B	Rank A	Rank B
ACTB_MOUSE	Actin, cytoplasmic 1 OS=Mus musculus GN=Actb PE=1 SV=1	63.3	52	2	1	7.2	4.3	6.31	3.38	105	121
A1AG1_MOUSE	Alpha-1-acid glycoprotein 1 OS=Mus musculus GN=Orm1 PE=1 SV=1	54.9	101.9	1	1	7.2	7.2	7.98	0.28	108	97
A1AT2_MOUSE	Alpha-1-antitrypsin 1-2 OS=Mus musculus GN=Serpina1b PE=1 SV=2	400.8	790.1	7	11	25.7	38	5.15	4.88	32	21
A1AT3_MOUSE	Alpha-1-antitrypsin 1-3 OS=Mus musculus GN=Serpina1c PE=1 SV=2	541.8	868.3	10	12	34.7	40.5	6.22	4.72	22	16
FETUA_MOUSE	Alpha-2-HS-glycoprotein OS=Mus musculus GN=Ahsg PE=1 SV=1	476.5	1281	8	15	33.3	50.7	2.55	3.32	28	11
APOA1_MOUSE	Apolipoprotein A-I OS=Mus musculus GN=Apoa1 PE=1 SV=2	845.9	1357.8	15	22	60.2	69.3	5.7	4.35	14	10
APOA2_MOUSE	Apolipoprotein A-II OS=Mus musculus GN=Apoa2 PE=1 SV=2	50.6	84.9	1	2	9.8	14.7	0.51	1.47	113	107
APOA4_MOUSE	Apolipoprotein A-IV OS=Mus musculus GN=Apoa4 PE=1 SV=3	321	903	9	13	30.4	47.3	5.15	4.26	39	15
APOC1_MOUSE	Apolipoprotein C-I OS=Mus musculus GN=Apoc1 PE=1 SV=1	41	89.8	1	2	10.2	20.5	5.99	5.48	116	102
APOC2_MOUSE	Apolipoprotein C-II OS=Mus musculus GN=Apoc2 PE=2 SV=1	145	270.7	3	4	30.9	33	1.93	4.95	73	59
APOC3_MOUSE	Apolipoprotein C-III OS=Mus musculus GN=Apoc3 PE=1 SV=2	306.7	397.3	4	5	50.5	50.5	4.2	6.21	40	41
APOE_MOUSE	Apolipoprotein E OS=Mus musculus GN=Apoe PE=1 SV=2	814.5	512.3	16	10	53.4	40.8	3.81	2.59	15	33
APOH_MOUSE	Beta-2-glycoprotein 1 OS=Mus musculus GN=Apoh PE=1 SV=1	111.9	857.7	3	15	11.9	46.7	5.88	3.28	84	17
CRP_MOUSE	C-reactive protein OS=Mus musculus GN=Crp PE=1 SV=2	73.2	270	3	7	15.6	35.1	3.21	4.72	101	60
CLC11_MOUSE	C-type lectin domain family 11 member A OS=Mus musculus GN=Clec11a PE=2 SV=1	40.5	0	1	0	2.7	0	7.93	0	119	0
C4BPA_MOUSE	C4b-binding protein OS=Mus musculus GN=C4bpa PE=1 SV=3	761.6	334.7	13	8	30.9	23	3.2	3.51	18	44
EST1C_MOUSE	Carboxylesterase 1C OS=Mus musculus GN=Ces1c PE=1 SV=4	44.4	0	1	0	5.1	0	4.14	0	115	0
CBPB2_MOUSE	Carboxypeptidase B2 OS=Mus musculus GN=Cpb2 PE=1 SV=1	81.5	0	2	0	3.8	0	5.93	0	95	0
CD5L_MOUSE	CD5 antigen-like OS=Mus musculus GN=Cd5l PE=1 SV=3	1106.3	1245.5	18	20	57.4	56.5	5.1	3.44	13	12
CLUS_MOUSE	Clusterin OS=Mus musculus GN=Clu PE=1 SV=1	400.8	788.3	7	14	21	32.4	5	2.45	33	22
FA5_MOUSE	Coagulation factor V OS=Mus musculus GN=F5 PE=1 SV=1	552.7	361.6	12	7	7.2	4.1	3.85	3.59	21	43
FA12_MOUSE	Coagulation factor XII OS=Mus musculus GN=F12 PE=1 SV=2	88.1	153	2	3	4.7	8	8.13	3.56	88	81
C1QA_MOUSE	Complement C1q subcomponent subunit A OS=Mus musculus GN=C1qa PE=1 SV=2	237.8	144.5	4	2	23.3	11.8	4.03	7.28	51	87
C1QB_MOUSE	Complement C1q subcomponent subunit B OS=Mus musculus GN=C1qb PE=1 SV=2	335.7	211.8	6	4	27.3	16.6	2.98	2.59	38	71
C1QC_MOUSE	Complement C1q subcomponent subunit C OS=Mus musculus GN=C1qc PE=1 SV=2	198.4	122.4	4	2	21.5	11.4	3.72	3.44	57	92
C1RA_MOUSE	Complement C1r-A subcomponent OS=Mus musculus GN=C1ra PE=1 SV=1	772.4	822.7	14	16	33.4	40.7	3.77	4.53	16	19
CS1A_MOUSE	Complement C1s-A subcomponent OS=Mus musculus GN=C1sa PE=2 SV=2	764.8	614.4	11	11	21.5	25.6	2.82	5.19	17	28
CO3_MOUSE	Complement C3 OS=Mus musculus GN=C3 PE=1 SV=3	5560.6	2923.1	92	53	68.6	40	3.81	3.1	1	2
CO4B_MOUSE	Complement C4-B OS=Mus musculus GN=C4b PE=1 SV=3	3716.6	1749.4	61	37	48.6	32.2	4.1	3.5	3	7
CO5_MOUSE	Complement C5 OS=Mus musculus GN=C5 PE=1 SV=2	2954.2	0	55	0	45.4	0	4.32	0	4	0
CO8A_MOUSE	Complement component C8 alpha chain OS=Mus musculus GN=C8a PE=1 SV=1	152.9	0	4	0	12.9	0	5.24	0	69	0
CO8B_MOUSE	Complement component C8 beta chain OS=Mus musculus GN=C8b PE=1 SV=1	261.1	0	5	0	12.4	0	4.96	0	48	0
CO8G_MOUSE	Complement component C8 gamma chain OS=Mus musculus GN=C8g PE=1 SV=1	82.2	0	1	0	5.4	0	1.19	0	94	0
CO9_MOUSE	Complement component C9 OS=Mus musculus GN=C9 PE=1 SV=2	133.4	0	2	0	4.2	0	4.6	0	77	0
CFAB_MOUSE	Complement factor B OS=Mus musculus GN=Cfb PE=1 SV=2	430.2	278.9	8	6	13.9	13.3	3.21	3.35	31	55
CFAD_MOUSE	Complement factor D OS=Mus musculus GN=Cfd PE=1 SV=1	40.7	61	1	2	8.5	10	6.67	2.73	117	118
CFAH_MOUSE	Complement factor H OS=Mus musculus GN=Cfh PE=1 SV=2	3983.7	2042.6	63	39	58.8	43.7	3.73	3.71	2	4
CFAI_MOUSE	Complement factor I OS=Mus musculus GN=Cfi PE=1 SV=3	51.2	113.3	1	3	2.2	5	7.51	3.87	112	95
ECM1_MOUSE	Extracellular matrix protein 1 OS=Mus musculus GN=Ecm1 PE=1 SV=2	40.5	61.2	1	2	1.8	4.8	4.32	4.99	118	117

FIBA_MOUSE	Fibrinogen alpha chain OS=Mus musculus GN=Fga PE=1 SV=1	176.8	321.7	4	5	7.5	13.2	5.76	3.2	59	46
FIBB_MOUSE	Fibrinogen beta chain OS=Mus musculus GN=Fgb PE=1 SV=1	135.7	0	3	0	9.4	0	5.51	0	74	0
FIBG_MOUSE	Fibrinogen gamma chain OS=Mus musculus GN=Fgg PE=1 SV=1	275.7	302.7	7	7	24.5	24.1	4.58	4.59	46	50
FINC_MOUSE	Fibronectin OS=Mus musculus GN=Fn1 PE=1 SV=4	2617.9	317.5	47	8	31.8	4	4.04	3.32	5	48
GELS_MOUSE	Gelsolin OS=Mus musculus GN=Gsn PE=1 SV=3	1362.6	1100.6	25	24	43.7	42.6	4.4	4.14	9	13
HA10_MOUSE	H-2 class I histocompatibility antigen, Q10 alpha chain OS=Mus musculus GN=H2-Q10 PE=1 SV=3	170	725.9	3	14	11.4	44.3	3.22	4.72	65	24
HBA_MOUSE	Hemoglobin subunit alpha OS=Mus musculus GN=Hba PE=1 SV=2	160.2	0	4	0	34.5	0	5.31	0	67	0
HBB1_MOUSE	Hemoglobin subunit beta-1 OS=Mus musculus GN=Hbb-b1 PE=1 SV=2	121.8	89.5	2	3	17.7	30.6	8.47	8.11	80	103
HEMO_MOUSE	Hemopexin OS=Mus musculus GN=Hpx PE=1 SV=2	305.6	1060.2	8	22	17.6	50.9	3.78	4.1	41	14
HRG_MOUSE	Histidine-rich glycoprotein OS=Mus musculus GN=Hrg PE=1 SV=2	438.6	666.7	10	13	20.4	29.3	5.5	4.31	30	26
IGHA_MOUSE	Ig alpha chain C region OS=Mus musculus PE=1 SV=1	172	0	4	0	17.7	0	3.79	0	61	0
IGHG1_MOUSE	Ig gamma-1 chain C region secreted form OS=Mus musculus GN=Ighg1 PE=1 SV=1	282	309.2	5	6	25.9	36.1	4.35	5.06	45	49
GCAA_MOUSE	Ig gamma-2A chain C region, A allele OS=Mus musculus GN=Ighg PE=1 SV=1	508.8	0	9	0	55.5	0	2.69	0	26	0
IGG2B_MOUSE	Ig gamma-2B chain C region OS=Mus musculus GN=Igh-3 PE=1 SV=3	519.3	294.2	8	6	26	22.8	2.79	2.9	24	52
IGHG3_MOUSE	Ig gamma-3 chain C region OS=Mus musculus PE=1 SV=2	1224.3	812	18	14	54.8	47	3.67	4.88	12	20
HVM63_MOUSE	Ig heavy chain Mem5 (Fragment) OS=Mus musculus PE=1 SV=1	204.2	0	4	0	44.7	0	4.29	0	54	0
HVM60_MOUSE	Ig heavy chain V region 3-6 OS=Mus musculus GN=Ighv3-6 PE=1 SV=1	96.4	92.6	1	1	13.8	13.8	0.91	0.23	86	100
HVM36_MOUSE	Ig heavy chain V region 441 OS=Mus musculus PE=4 SV=1	250.9	0	6	0	57.8	0	3.5	0	49	0
HVM54_MOUSE	Ig heavy chain V region 5-84 OS=Mus musculus PE=1 SV=1	124.3	148.1	2	4	17.9	36.8	5.64	4.58	79	84
HVM57_MOUSE	Ig heavy chain V region 6.96 OS=Mus musculus PE=4 SV=1	75.1	86.1	2	2	24.5	24.5	3.53	5.77	99	105
HVM56_MOUSE	Ig heavy chain V region 914 OS=Mus musculus PE=1 SV=1	77.3	93.7	2	3	16.5	33	4.71	2.67	97	99
HVM51_MOUSE	Ig heavy chain V region AC38 205.12 OS=Mus musculus PE=1 SV=1	296.4	289.9	3	3	33.9	33.9	5.52	4.28	43	53
HVM21_MOUSE	Ig heavy chain V region M511 OS=Mus musculus PE=1 SV=1	301.9	437.8	6	7	55.7	71.3	1.54	2.65	42	40
HVM45_MOUSE	Ig heavy chain V region MC101 OS=Mus musculus PE=1 SV=1	55.4	52.2	2	2	33.6	33.6	6.56	6.33	107	120
HVM16_MOUSE	Ig heavy chain V region MOPC 21 (Fragment) OS=Mus musculus PE=1 SV=1	76.8	226.3	2	4	11.8	26.5	6.44	4.54	98	67
HVM17_MOUSE	Ig heavy chain V region MOPC 47A OS=Mus musculus PE=1 SV=1	173.3	224.2	4	4	35	35	3.37	2.21	60	68
HVM00_MOUSE	Ig heavy chain V region OS=Mus musculus PE=1 SV=1	66.5	40.5	1	1	11.4	11.4	8.15	7.51	102	124
HVM35_MOUSE	Ig heavy chain V-III region HPC76 (Fragment) OS=Mus musculus PE=4 SV=1	84.8	0	2	0	12.6	0	4.33	0	93	0
IGKC_MOUSE	Ig kappa chain C region OS=Mus musculus PE=1 SV=1	1269.2	1759.2	13	16	78.3	78.3	5.03	3.75	11	6
KV2A7_MOUSE	Ig kappa chain V-II region 26-10 OS=Mus musculus PE=1 SV=1	243.7	299.5	4	4	43.4	43.4	2.76	2.67	50	51
KV3A4_MOUSE	Ig kappa chain V-III region 50S10.1 OS=Mus musculus PE=1 SV=1	265.2	228.9	3	3	40.5	40.5	2.18	1.58	47	65
KV3AM_MOUSE	Ig kappa chain V-III region PC 2154 OS=Mus musculus PE=1 SV=1	85.5	80.1	1	1	11.1	11.1	3.68	3.25	91	111
KV3A8_MOUSE	Ig kappa chain V-III region PC 3741/TEPC 111 OS=Mus musculus PE=1 SV=1	220.3	194.8	3	3	30.6	30.6	5	4.52	52	76
KV3AB_MOUSE	Ig kappa chain V-III region PC 4050 OS=Mus musculus PE=1 SV=1	289.9	260.3	3	3	30.6	30.6	0.96	5.39	44	62
KV3AJ_MOUSE	Ig kappa chain V-III region PC 7175 OS=Mus musculus PE=1 SV=1	354.7	522.1	4	5	68.5	88.3	2.46	5.6	37	32
KV3AG_MOUSE	Ig kappa chain V-III region PC 7210 OS=Mus musculus PE=1 SV=1	202.2	203.8	2	2	30.9	30.9	2.62	1.51	55	75
KV4A1_MOUSE	Ig kappa chain V-IV region S107B OS=Mus musculus PE=4 SV=1	62.6	61.6	1	1	8.5	8.5	0.32	2.24	106	115
KV5AB_MOUSE	Ig kappa chain V-V region HP R16.7 OS=Mus musculus PE=1 SV=1	189.3	276.9	4	5	49.1	59.3	4.74	4.97	58	57
KV5A3_MOUSE	Ig kappa chain V-V region K2 (Fragment) OS=Mus musculus PE=1 SV=1	127.3	206.8	2	4	19.1	40.9	1.21	1.41	78	74
KV5A6_MOUSE	Ig kappa chain V-V region L6 (Fragment) OS=Mus musculus PE=4 SV=1	146.6	210.4	3	3	35.7	35.7	5.97	4.8	72	72
KV5A4_MOUSE	Ig kappa chain V-V region MOPC 149 OS=Mus musculus PE=1 SV=1	200.1	228.8	2	2	25.9	25.9	2.43	1.28	56	66
KV5A7_MOUSE	Ig kappa chain V-V region MOPC 41 OS=Mus musculus GN=Gm5571 PE=1 SV=1	148.7	334.4	3	4	28.5	28.5	5.11	5.89	71	45
KV6A5_MOUSE	Ig kappa chain V-VI region J539 OS=Mus musculus PE=1 SV=1	134.4	84.2	2	2	24.3	24.3	2.75	4.83	76	108
KV6AB_MOUSE	Ig kappa chain V-VI region NQ2-6.1 OS=Mus musculus PE=2 SV=1	73.6	133.4	1	1	14.8	14.8	4.09	4.37	100	90
KV5A1_MOUSE	Ig kappa chain V19-17 OS=Mus musculus GN=Igk-V19-17 PE=1 SV=1	153.2	178	3	4	16.8	16.1	1.79	5.33	68	77
LAC1_MOUSE	Ig lambda-1 chain C region OS=Mus musculus PE=1 SV=1	152.4	0	3	0	46.7	0	4.85	0	70	0
LV1B_MOUSE	Ig lambda-1 chain V regions MOPC 104E/RPC20/J558/S104 OS=Mus musculus PE=1 SV=1	65	83.8	1	2	25.6	32.6	1.43	7.2	104	110
LAC2_MOUSE	Ig lambda-2 chain C region OS=Mus musculus GN=Iglc2 PE=1 SV=1	119.4	0	2	0	35.6	0	0.54	0	81	0
IGHM_MOUSE	Ig mu chain C region OS=Mus musculus GN=Ighm PE=1 SV=2	2212.8	2512.5	32	38	68.5	69.6	2.57	3.03	8	3
IGJ_MOUSE	Immunoglobulin J chain OS=Mus musculus GN=Jchain PE=1 SV=4	362.5	579.3	7	11	35.8	59.1	3.26	4.1	36	30
ITIH4_MOUSE	Inter alpha-trypsin inhibitor, heavy chain 4 OS=Mus musculus GN=Itih4 PE=1 SV=2	1355.7	1445.4	25	26	39.8	45.5	4.42	4.35	10	8
ITIH3_MOUSE	Inter-alpha-trypsin inhibitor heavy chain H3 OS=Mus musculus GN=Itih3 PE=1 SV=3	114.7	0	2	0	2.9	0	7.26	0	83	0

K1C10_MOUSE	Keratin, type I cytoskeletal 10 OS=Mus musculus GN=Krt10 PE=1 SV=3	171.1	0	4	0	7.5	0	4.38	0	62	0
K1C15_MOUSE	Keratin, type I cytoskeletal 15 OS=Mus musculus GN=Krt15 PE=1 SV=2	86.5	0	2	0	3.5	0	2.91	0	90	0
K22E_MOUSE	Keratin, type II cytoskeletal 2 epidermal OS=Mus musculus GN=Krt2 PE=1 SV=1	53.8	0	1	0	2	0	5.94	0	110	0
K2C6A_MOUSE	Keratin, type II cytoskeletal 6A OS=Mus musculus GN=Krt6a PE=1 SV=3	85.4	0	1	0	2.2	0	6.95	0	92	0
K2C73_MOUSE	Keratin, type II cytoskeletal 73 OS=Mus musculus GN=Krt73 PE=1 SV=1	87.8	0	2	0	4.3	0	4.29	0	89	0
K2C79_MOUSE	Keratin, type II cytoskeletal 79 OS=Mus musculus GN=Krt79 PE=1 SV=2	66	0	1	0	2.3	0	7.69	0	103	0
KNG1_MOUSE	Kininogen-1 OS=Mus musculus GN=Kng1 PE=1 SV=1	170.8	623.2	3	11	6.7	21.3	6.4	4.83	63	27
MASP1_MOUSE	Mannan-binding lectin serine protease 1 OS=Mus musculus GN=Masp1 PE=1 SV=2	397.1	214.3	8	5	12.9	8.8	2.5	3.59	34	70
MASP2_MOUSE	Mannan-binding lectin serine protease 2 OS=Mus musculus GN=Masp2 PE=1 SV=1	519.2	237.2	10	6	18.4	12.8	3.63	3.77	25	63
MBL1_MOUSE	Mannose-binding protein A OS=Mus musculus GN=Mbl1 PE=1 SV=1	502.1	146.3	9	3	44.8	13.4	3.32	3.05	27	85
MBL2_MOUSE	Mannose-binding protein C OS=Mus musculus GN=Mbl2 PE=1 SV=2	584.7	598.7	8	10	37.3	44.7	4.46	4.49	20	29
TIMP3_MOUSE	Metalloproteinase inhibitor 3 OS=Mus musculus GN=Timp3 PE=1 SV=1	51.8	0	2	0	10	0	3.35	0	111	0
MUG1_MOUSE	Murinoglobulin-1 OS=Mus musculus GN=Mug1 PE=1 SV=3	135.3	458	3	9	2.8	10.7	3.76	5.56	75	39
PEDF_MOUSE	Pigment epithelium-derived factor OS=Mus musculus GN=Serpinf1 PE=1 SV=2	94.5	0	3	0	12.9	0	6.52	0	87	0
PLMN_MOUSE	Plasminogen OS=Mus musculus GN=Plg PE=1 SV=3	623.2	207	15	5	26.1	6.9	3.96	4.66	19	73
PLF4_MOUSE	Platelet factor 4 OS=Mus musculus GN=Pf4 PE=1 SV=1	45.3	0	1	0	11.4	0	3.3	0	114	0
GP1BA_MOUSE	Platelet glycoprotein Ib alpha chain OS=Mus musculus GN=Gp1ba PE=1 SV=2	170.3	0	4	0	7.4	0	4.46	0	64	0
PZP_MOUSE	Pregnancy zone protein OS=Mus musculus GN=Pzp PE=1 SV=3	215.1	732.7	6	20	3.5	22	4.98	4.61	53	23
PROP_MOUSE	Properdin OS=Mus musculus GN=Cfp PE=2 SV=2	385.6	278	7	6	24.8	19.6	2.28	2.47	35	56
AMBP_MOUSE	Protein AMBP OS=Mus musculus GN=Ambp PE=1 SV=2	79.1	0	2	0	7.2	0	6.74	0	96	0
THRB_MOUSE	Prothrombin OS=Mus musculus GN=F2 PE=1 SV=1	117.9	682.4	4	12	6.6	19.7	2.66	4.35	82	25
SEPP1_MOUSE	Selenoprotein P OS=Mus musculus GN=Sepp1 PE=1 SV=3	54.5	86.9	2	3	8.4	14.7	6.59	3.45	109	104
SPA3K_MOUSE	Serine protease inhibitor A3K OS=Mus musculus GN=Serpina3k PE=1 SV=2	464.6	823	10	14	39.2	41.9	4.1	4.07	29	18
TRFE_MOUSE	Serotransferrin OS=Mus musculus GN=Tf PE=1 SV=1	537.2	1368.7	15	25	28.7	45.3	2.57	4.48	23	9
ALBU_MOUSE	Serum albumin OS=Mus musculus GN=Alb PE=1 SV=3	2330.9	3116.6	39	46	71.4	76.8	4.74	3.52	7	1
TSP1_MOUSE	Thrombospondin-1 OS=Mus musculus GN=Thbs1 PE=1 SV=1	2500.2	1965	47	41	43.9	43.8	2.97	3.8	6	5
VTDB_MOUSE	Vitamin D-binding protein OS=Mus musculus GN=Gc PE=1 SV=2	104.4	480.2	1	10	4.6	37.4	3.75	3.96	85	35
VTNC_MOUSE	Vitronectin OS=Mus musculus GN=Vtn PE=1 SV=2	168.1	235.9	4	5	12.8	18.8	2.51	2.79	66	64

Supplemental Data Table 3. Normal Serum Venn Diagram Identifications

Comparison of Proteins Associated with As Prepared and 2h Ball Milled SWCNTs
Accession Number

In Common	As Prepared Only	2h Ball Milled Only
A1AG1_MOUSE	ANT3_MOUSE	BPIA2_MOUSE
A1AT2_MOUSE	B2MG_MOUSE	FIBB_MOUSE
A1AT3_MOUSE	CERU_MOUSE	HBA_MOUSE
ACTB_MOUSE	CFAD_MOUSE	HVM09_MOUSE
ALBU_MOUSE	ECM1_MOUSE	HVM27_MOUSE
APOA1_MOUSE	F13B_MOUSE	HVM36_MOUSE
APOA2_MOUSE	FA12_MOUSE	HVM44_MOUSE
APOA4_MOUSE	G3P_MOUSE	IGHA_MOUSE
APOC1_MOUSE	HBB1_MOUSE	K2C6A_MOUSE
APOC2_MOUSE	HEPC_MOUSE	KVM5_MOUSE
APOC3_MOUSE	HVM13_MOUSE	LAC1_MOUSE
APOE_MOUSE	HVM32_MOUSE	PLF4_MOUSE
APOH_MOUSE	HVM37_MOUSE	
C1QA_MOUSE	HVM53_MOUSE	
C1QB_MOUSE	IBP2_MOUSE	
C1QC_MOUSE	IBP4_MOUSE	
C1RA_MOUSE	KV2A5_MOUSE	
C4BPA_MOUSE	KV3AB_MOUSE	
CD5L_MOUSE	KV5AG_MOUSE	
CFAB_MOUSE	LV1B_MOUSE	
CFAH_MOUSE	MUP1_MOUSE	
CFAI_MOUSE	MUP2_MOUSE	
CLUS_MOUSE	QSOX1_MOUSE	
CO3_MOUSE	RET4_MOUSE	
CO4B_MOUSE	SBSN_MOUSE	
CRP_MOUSE	SEPP1_MOUSE	
CS1A_MOUSE	TETN_MOUSE	
FA5_MOUSE		
FETUA_MOUSE		
FETUB_MOUSE		
FIBA_MOUSE		
FIBG_MOUSE		
FINC_MOUSE		
GCAM_MOUSE		

GELS_MOUSE
HA10_MOUSE
HEMO_MOUSE
HPT_MOUSE
HRG_MOUSE
HVM00_MOUSE
HVM05_MOUSE
HVM06_MOUSE
HVM16_MOUSE
HVM17_MOUSE
HVM21_MOUSE
HVM45_MOUSE
HVM51_MOUSE
HVM54_MOUSE
HVM56_MOUSE
HVM57_MOUSE
HVM60_MOUSE
IC1_MOUSE
IGG2B_MOUSE
IGHG1_MOUSE
IGHG3_MOUSE
IGHM_MOUSE
IGJ_MOUSE
IGKC_MOUSE
ITIH4_MOUSE
KNG1_MOUSE
KV2A6_MOUSE
KV2A7_MOUSE
KV3A4_MOUSE
KV3A8_MOUSE
KV3AG_MOUSE
KV3AI_MOUSE
KV3AJ_MOUSE
KV3AM_MOUSE
KV4A1_MOUSE
KV5A1_MOUSE
KV5A3_MOUSE
KV5A4_MOUSE
KV5A6_MOUSE
KV5A7_MOUSE
KV5A9_MOUSE

KV5AB_MOUSE
KV6A5_MOUSE
KV6A7_MOUSE
KV6AB_MOUSE
LOXL1_MOUSE
MASP1_MOUSE
MASP2_MOUSE
MBL1_MOUSE
MBL2_MOUSE
MUG1_MOUSE
NPY_MOUSE
PLMN_MOUSE
PROP_MOUSE
PZP_MOUSE
SAA1_MOUSE
SAMP_MOUSE
SPA3K_MOUSE
THRB_MOUSE
TRFE_MOUSE
TSP1_MOUSE
TTHY_MOUSE
VTDB_MOUSE
VTNC_MOUSE

Comparison of Proteins Associated with As Prepared and 4h Ball Milled SWCNTs
Accession Number

**Shared**	**As Prepared Only**	**4h Ball Milled Only**
A1AT3_MOUSE	A1AG1_MOUSE	A1AT4_MOUSE
ALBU_MOUSE	A1AT2_MOUSE	ACTG_MOUSE
ANT3_MOUSE	ACTB_MOUSE	BPIA2_MOUSE
APOA1_MOUSE	B2MG_MOUSE	EST1C_MOUSE
APOA2_MOUSE	CERU_MOUSE	FIBB_MOUSE
APOA4_MOUSE	CFAD_MOUSE	GCAA_MOUSE
APOC1_MOUSE	CFAI_MOUSE	HBA_MOUSE
APOC2_MOUSE	F13B_MOUSE	HVM09_MOUSE
APOC3_MOUSE	FA12_MOUSE	HVM36_MOUSE
APOE_MOUSE	G3P_MOUSE	HVM44_MOUSE
APOH_MOUSE	GCAM_MOUSE	K1C10_MOUSE
C1QA_MOUSE	HEPC_MOUSE	K1C13_MOUSE

C1QB_MOUSE
C1QC_MOUSE
C1RA_MOUSE
C4BPA_MOUSE
CD5L_MOUSE
CFAB_MOUSE
CFAH_MOUSE
CLUS_MOUSE
CO3_MOUSE
CO4B_MOUSE
CRP_MOUSE
CS1A_MOUSE
ECM1_MOUSE
FA5_MOUSE
FETUA_MOUSE
FETUB_MOUSE
FIBA_MOUSE
FIBG_MOUSE
FINC_MOUSE
GELS_MOUSE
HA10_MOUSE
HBB1_MOUSE
HEMO_MOUSE
HPT_MOUSE
HRG_MOUSE
HVM00_MOUSE
HVM05_MOUSE
HVM06_MOUSE
HVM16_MOUSE
HVM17_MOUSE
HVM21_MOUSE
HVM32_MOUSE
HVM45_MOUSE
HVM51_MOUSE
HVM53_MOUSE
HVM54_MOUSE
HVM56_MOUSE
HVM57_MOUSE
HVM60_MOUSE
IC1_MOUSE
IGG2B_MOUSE
HVM13_MOUSE
HVM37_MOUSE
IBP2_MOUSE
IBP4_MOUSE
KV3AG_MOUSE
KV4A1_MOUSE
KV5AB_MOUSE
KV5AG_MOUSE
KV6A7_MOUSE
LV1B_MOUSE
MUP1_MOUSE
MUP2_MOUSE
QSOX1_MOUSE
RET4_MOUSE
SBSN_MOUSE
SEPP1_MOUSE
TETN_MOUSE
K22E_MOUSE
K2C74_MOUSE
KV3A5_MOUSE
KV3AE_MOUSE
KV5AA_MOUSE
KV5AC_MOUSE
KV5AF_MOUSE
KV6AA_MOUSE
LAC1_MOUSE
LAC2_MOUSE
LV1A_MOUSE
PLF4_MOUSE
SAA2_MOUSE

IGHG1_MOUSE
IGHG3_MOUSE
IGHM_MOUSE
IGJ_MOUSE
IGKC_MOUSE
ITIH4_MOUSE
KNG1_MOUSE
KV2A5_MOUSE
KV2A6_MOUSE
KV2A7_MOUSE
KV3A4_MOUSE
KV3A8_MOUSE
KV3AB_MOUSE
KV3AI_MOUSE
KV3AJ_MOUSE
KV3AM_MOUSE
KV5A1_MOUSE
KV5A3_MOUSE
KV5A4_MOUSE
KV5A6_MOUSE
KV5A7_MOUSE
KV5A9_MOUSE
KV6A5_MOUSE
KV6AB_MOUSE
LOXL1_MOUSE
MASP1_MOUSE
MASP2_MOUSE
MBL1_MOUSE
MBL2_MOUSE
MUG1_MOUSE
NPY_MOUSE
PLMN_MOUSE
PROP_MOUSE
PZP_MOUSE
SAA1_MOUSE
SAMP_MOUSE
SPA3K_MOUSE
THRB_MOUSE
TRFE_MOUSE
TSP1_MOUSE
TTHY_MOUSE

VTDB_MOUSE
VTNC_MOUSE

Comparison of Proteins Associated with As Prepared and 6h Ball Milled SWCNTs
Accession Number

**Shared**	**As Prepared Only**	**6h Ball Milled Only**
A1AT3_MOUSE	A1AG1_MOUSE	FIBB_MOUSE
ACTB_MOUSE	A1AT2_MOUSE	GCAA_MOUSE
ALBU_MOUSE	B2MG_MOUSE	HVM09_MOUSE
ANT3_MOUSE	CERU_MOUSE	HVM18_MOUSE
APOA1_MOUSE	CFAD_MOUSE	HVM35_MOUSE
APOA2_MOUSE	CFAI_MOUSE	HVM36_MOUSE
APOA4_MOUSE	ECM1_MOUSE	IGHA_MOUSE
APOC1_MOUSE	F13B_MOUSE	K22E_MOUSE
APOC2_MOUSE	FA12_MOUSE	K2C74_MOUSE
APOC3_MOUSE	FETUB_MOUSE	KV6A6_MOUSE
APOE_MOUSE	G3P_MOUSE	KVM5_MOUSE
APOH_MOUSE	GCAM_MOUSE	PLF4_MOUSE
C1QA_MOUSE	HEPC_MOUSE	SPTB1_MOUSE
C1QB_MOUSE	HPT_MOUSE	
C1QC_MOUSE	HVM13_MOUSE	
C1RA_MOUSE	HVM21_MOUSE	
C4BPA_MOUSE	HVM32_MOUSE	
CD5L_MOUSE	HVM37_MOUSE	
CFAB_MOUSE	HVM53_MOUSE	
CFAH_MOUSE	IBP2_MOUSE	
CLUS_MOUSE	IBP4_MOUSE	
CO3_MOUSE	KV2A5_MOUSE	
CO4B_MOUSE	KV5AG_MOUSE	
CRP_MOUSE	KV6A7_MOUSE	
CS1A_MOUSE	LV1B_MOUSE	
FA5_MOUSE	MUP1_MOUSE	
FETUA_MOUSE	MUP2_MOUSE	
FIBA_MOUSE	NPY_MOUSE	
FIBG_MOUSE	QSOX1_MOUSE	
FINC_MOUSE	RET4_MOUSE	
GELS_MOUSE	SBSN_MOUSE	
HA10_MOUSE	SEPP1_MOUSE	
HBB1_MOUSE	TETN_MOUSE	

HEMO_MOUSE
HRG_MOUSE
HVM00_MOUSE
HVM05_MOUSE
HVM06_MOUSE
HVM16_MOUSE
HVM17_MOUSE
HVM45_MOUSE
HVM51_MOUSE
HVM54_MOUSE
HVM56_MOUSE
HVM57_MOUSE
HVM60_MOUSE
IC1_MOUSE
IGG2B_MOUSE
IGHG1_MOUSE
IGHG3_MOUSE
IGHM_MOUSE
IGJ_MOUSE
IGKC_MOUSE
ITIH4_MOUSE
KNG1_MOUSE
KV2A6_MOUSE
KV2A7_MOUSE
KV3A4_MOUSE
KV3A8_MOUSE
KV3AB_MOUSE
KV3AG_MOUSE
KV3AI_MOUSE
KV3AJ_MOUSE
KV3AM_MOUSE
KV4A1_MOUSE
KV5A1_MOUSE
KV5A3_MOUSE
KV5A4_MOUSE
KV5A6_MOUSE
KV5A7_MOUSE
KV5A9_MOUSE
KV5AB_MOUSE
KV6A5_MOUSE
KV6AB_MOUSE

LOXL1_MOUSE
MASP1_MOUSE
MASP2_MOUSE
MBL1_MOUSE
MBL2_MOUSE
MUG1_MOUSE
PLMN_MOUSE
PROP_MOUSE
PZP_MOUSE
SAA1_MOUSE
SAMP_MOUSE
SPA3K_MOUSE
THRB_MOUSE
TRFE_MOUSE
TSP1_MOUSE
TTHY_MOUSE
VTDB_MOUSE
VTNC_MOUSE

Comparison of Proteins Associated with As Prepared and 8h Ball Milled SWCNTs
Accession Number

**Shared**	**As Prepared Only**	**8h Ball Milled Only**
A1AG1_MOUSE	ANT3_MOUSE	AMBP_MOUSE
A1AT2_MOUSE	B2MG_MOUSE	CBPB2_MOUSE
A1AT3_MOUSE	CERU_MOUSE	CLC11_MOUSE
ACTB_MOUSE	F13B_MOUSE	CO5_MOUSE
ALBU_MOUSE	FETUB_MOUSE	CO8A_MOUSE
APOA1_MOUSE	G3P_MOUSE	CO8B_MOUSE
APOA2_MOUSE	GCAM_MOUSE	CO8G_MOUSE
APOA4_MOUSE	HEPC_MOUSE	CO9_MOUSE
APOC1_MOUSE	HPT_MOUSE	EST1C_MOUSE
APOC2_MOUSE	HVM05_MOUSE	FIBB_MOUSE
APOC3_MOUSE	HVM06_MOUSE	GCAA_MOUSE
APOE_MOUSE	HVM13_MOUSE	GP1BA_MOUSE
APOH_MOUSE	HVM32_MOUSE	HBA_MOUSE
C1QA_MOUSE	HVM37_MOUSE	HVM35_MOUSE
C1QB_MOUSE	HVM53_MOUSE	HVM36_MOUSE
C1QC_MOUSE	IBP2_MOUSE	HVM63_MOUSE
C1RA_MOUSE	IBP4_MOUSE	IGHA_MOUSE

C4BPA_MOUSE
CD5L_MOUSE
CFAB_MOUSE
CFAD_MOUSE
CFAH_MOUSE
CFAI_MOUSE
CLUS_MOUSE
CO3_MOUSE
CO4B_MOUSE
CRP_MOUSE
CS1A_MOUSE
ECM1_MOUSE
FA12_MOUSE
FA5_MOUSE
FETUA_MOUSE
FIBA_MOUSE
FIBG_MOUSE
FINC_MOUSE
GELS_MOUSE
HA10_MOUSE
HBB1_MOUSE
HEMO_MOUSE
HRG_MOUSE
HVM00_MOUSE
HVM16_MOUSE
HVM17_MOUSE
HVM21_MOUSE
HVM45_MOUSE
HVM51_MOUSE
HVM54_MOUSE
HVM56_MOUSE
HVM57_MOUSE
HVM60_MOUSE
IGG2B_MOUSE
IGHG1_MOUSE
IGHG3_MOUSE
IGHM_MOUSE
IGJ_MOUSE
IGKC_MOUSE
ITIH4_MOUSE
KNG1_MOUSE
IC1_MOUSE
KV2A5_MOUSE
KV2A6_MOUSE
KV3AI_MOUSE
KV5A9_MOUSE
KV5AG_MOUSE
KV6A7_MOUSE
LOXL1_MOUSE
MUP1_MOUSE
MUP2_MOUSE
NPY_MOUSE
QSOX1_MOUSE
RET4_MOUSE
SAA1_MOUSE
SAMP_MOUSE
SBSN_MOUSE
TETN_MOUSE
TTHY_MOUSE
ITIH3_MOUSE
K1C10_MOUSE
K1C15_MOUSE
K22E_MOUSE
K2C6A_MOUSE
K2C73_MOUSE
K2C79_MOUSE
LAC1_MOUSE
LAC2_MOUSE
PEDF_MOUSE
PLF4_MOUSE
TIMP3_MOUSE

KV2A7_MOUSE
KV3A4_MOUSE
KV3A8_MOUSE
KV3AB_MOUSE
KV3AG_MOUSE
KV3AJ_MOUSE
KV3AM_MOUSE
KV4A1_MOUSE
KV5A1_MOUSE
KV5A3_MOUSE
KV5A4_MOUSE
KV5A6_MOUSE
KV5A7_MOUSE
KV5AB_MOUSE
KV6A5_MOUSE
KV6AB_MOUSE
LV1B_MOUSE
MASP1_MOUSE
MASP2_MOUSE
MBL1_MOUSE
MBL2_MOUSE
MUG1_MOUSE
PLMN_MOUSE
PROP_MOUSE
PZP_MOUSE
SEPP1_MOUSE
SPA3K_MOUSE
THRB_MOUSE
TRFE_MOUSE
TSP1_MOUSE
VTDB_MOUSE
VTNC_MOUSE

Supplemental Table 2. All Identified Proteins on SWCNTs following Incubation in Hyperlipidemic Serum

As Prepared SWCNTs

Accession	Name	Meta Score A	Meta Score B	Peptides A	Peptides B	SC [%] A	SC [%] B	RMS90 [ppm] A	RMS90 [ppm] B	Rank A	Rank B
A1AG1_MOUSE	Alpha-1-acid glycoprotein 1 OS=Mus musculus GN=Orm1 PE=1 SV=1	89.1	101.9	1	1	7.2	7.2	0.63	0.28	88	97
A1AT2_MOUSE	Alpha-1-antitrypsin 1-2 OS=Mus musculus GN=Serpina1b PE=1 SV=2	0	790.1	0	11	0	38	0	4.88	0	21
A1AT3_MOUSE	Alpha-1-antitrypsin 1-3 OS=Mus musculus GN=Serpina1c PE=1 SV=2	176.8	868.3	6	12	20.4	40.5	3.30	4.72	58	16
ACTB_MOUSE	Actin, cytoplasmic 1 OS=Mus musculus GN=Actb PE=1 SV=1	0	52	0	1	0	4.3	0	3.38	0	121
ALBU_MOUSE	Serum albumin OS=Mus musculus GN=Alb PE=1 SV=3	2371.7	3116.6	37	46	74.2	76.8	4.30	3.52	2	1
ANT3_MOUSE	Antithrombin-III OS=Mus musculus GN=Serpinc1 PE=1 SV=1	0	92	0	2	0	7.1	0	7.03	0	101
APOA1_MOUSE	Apolipoprotein A-I OS=Mus musculus GN=Apoa1 PE=1 SV=2	1201.0	1357.8	21	22	69.3	69.3	4.19	4.35	6	10
APOA2_MOUSE	Apolipoprotein A-II OS=Mus musculus GN=Apoa2 PE=1 SV=2	124.5	84.9	2	2	32.4	14.7	2.64	1.47	75	107
APOA4_MOUSE	Apolipoprotein A-IV OS=Mus musculus GN=Apoa4 PE=1 SV=3	935.3	903.0	15	13	50.6	47.3	3.91	4.26	10	15
APOC1_MOUSE	Apolipoprotein C-I OS=Mus musculus GN=Apoc1 PE=1 SV=1	153.9	89.8	3	2	22.7	20.5	6.30	5.48	66	102
APOC2_MOUSE	Apolipoprotein C-II OS=Mus musculus GN=Apoc2 PE=2 SV=1	283.1	270.7	4	4	33.0	33.0	1.59	4.95	38	59
APOC3_MOUSE	Apolipoprotein C-III OS=Mus musculus GN=Apoc3 PE=1 SV=2	398.1	397.3	5	5	50.5	50.5	4.58	6.21	27	41
APOE_MOUSE	Apolipoprotein E OS=Mus musculus GN=Apoe PE=1 SV=2	1087.7	512.3	21	10	50.2	40.8	3.59	2.59	8	33
APOH_MOUSE	Beta-2-glycoprotein 1 OS=Mus musculus GN=Apoh PE=1 SV=1	330.3	857.7	9	15	29.0	46.7	4.17	3.28	31	17
B2MG_MOUSE	Beta-2-microglobulin OS=Mus musculus GN=B2m PE=1 SV=2	58.5	155.0	1	4	7.6	42.0	4.06	3.00	101	80
C1QA_MOUSE	Complement C1q subcomponent subunit A OS=Mus musculus GN=C1qa PE=1 SV=2	129.0	144.5	2	2	14.3	11.8	3.49	7.28	73	87
C1QB_MOUSE	Complement C1q subcomponent subunit B OS=Mus musculus GN=C1qb PE=1 SV=2	433.2	211.8	7	4	36.0	16.6	3.98	2.59	24	71
C1QC_MOUSE	Complement C1q subcomponent subunit C OS=Mus musculus GN=C1qc PE=1 SV=2	94.3	122.4	1	2	7.3	11.4	5.42	3.44	86	92
C1RA_MOUSE	Complement C1r-A subcomponent OS=Mus musculus GN=C1ra PE=1 SV=1	621.0	822.7	13	16	34.8	40.7	4.05	4.53	14	19
C4BPA_MOUSE	C4b-binding protein OS=Mus musculus GN=C4bpa PE=1 SV=3	405.3	334.7	7	8	19.8	23.0	3.16	3.51	26	44
CD5L_MOUSE	CD5 antigen-like OS=Mus musculus GN=Cd5l PE=1 SV=3	1354.7	1245.5	23	20	60.5	56.5	3.82	3.44	5	12
CERU_MOUSE	Ceruloplasmin OS=Mus musculus GN=Cp PE=1 SV=2	57.2	56.4	2	1	1.7	0.8	3.77	4.34	103	119
CFAB_MOUSE	Complement factor B OS=Mus musculus GN=Cfb PE=1 SV=2	119.2	278.9	3	6	6.7	13.3	5.86	3.35	77	55
CFAD_MOUSE	Complement factor D OS=Mus musculus GN=Cfd PE=1 SV=1	0	61	0	2	0	10	0	2.73	0	118
CFAH_MOUSE	Complement factor H OS=Mus musculus GN=Cfh PE=1 SV=2	1599.5	2042.6	32	39	39.7	43.7	5.38	3.71	4	4
CFAI_MOUSE	Complement factor I OS=Mus musculus GN=Cfi PE=1 SV=3	0	113.3	0	3	0	5	0	3.87	0	95
CLUS_MOUSE	Clusterin OS=Mus musculus GN=Clu PE=1 SV=1	653.4	788.3	13	14	31.7	32.4	4.81	2.45	13	22
CO3_MOUSE	Complement C3 OS=Mus musculus GN=C3 PE=1 SV=3	3021.5	2923.1	60	53	43.4	40.0	4.69	3.10	1	2
CO4B_MOUSE	Complement C4-B OS=Mus musculus GN=C4b PE=1 SV=3	464.2	1749.4	11	37	10.2	32.2	4.13	3.50	22	7
CRP_MOUSE	C-reactive protein OS=Mus musculus GN=Crp PE=1 SV=2	239.7	270.0	6	7	38.2	35.1	2.10	4.72	49	60
CS1A_MOUSE	Complement C1s-A subcomponent OS=Mus musculus GN=C1sa PE=2 SV=2	298.8	614.4	5	11	12.1	25.6	5.30	5.19	36	28
ECM1_MOUSE	Extracellular matrix protein 1 OS=Mus musculus GN=Ecm1 PE=1 SV=2	0	61.2	0	2	0	4.8	0	4.99	0	117
F13B_MOUSE	Coagulation factor XIII B chain OS=Mus musculus GN=F13b PE=1 SV=2	135.5	275.2	3	8	5.4	20.2	5.90	4.87	71	58
FA12_MOUSE	Coagulation factor XII OS=Mus musculus GN=F12 PE=1 SV=2	51.2	153.0	2	3	5.0	8.0	7.07	3.56	106	81
FA5_MOUSE	Coagulation factor V OS=Mus musculus GN=F5 PE=1 SV=1	178.0	361.6	4	7	2.7	4.1	6.11	3.59	57	43
FETUA_MOUSE	Alpha-2-HS-glycoprotein OS=Mus musculus GN=Ahsg PE=1 SV=1	988.8	1281.0	12	15	35.9	50.7	2.97	3.32	9	11
FETUB_MOUSE	Fetuin-B OS=Mus musculus GN=Fetub PE=1 SV=1	78.8	141.2	2	3	9.5	12.4	7.67	4.18	95	88
FIBA_MOUSE	Fibrinogen alpha chain OS=Mus musculus GN=Fga PE=1 SV=1	240.4	321.7	4	5	8.4	13.2	6.87	3.20	48	46
FIBG_MOUSE	Fibrinogen gamma chain OS=Mus musculus GN=Fgg PE=1 SV=1	135.4	302.7	4	7	15.4	24.1	3.13	4.59	72	50
FINC_MOUSE	Fibronectin OS=Mus musculus GN=Fn1 PE=1 SV=4	346.1	317.5	8	8	5.4	4.0	4.41	3.32	30	48
G3P_MOUSE	Glyceraldehyde-3-phosphate dehydrogenase OS=Mus musculus GN=Gapdh PE=1 SV=2	0	50.3	0	2	0	7.8	0	6.79	0	123
GCAM_MOUSE	Ig gamma-2A chain C region, membrane-bound form OS=Mus musculus GN=Igh-1a PE=1 SV=3	0	480.1	0	9	0	29.9	0	4.33	0	36
GELS_MOUSE	Gelsolin OS=Mus musculus GN=Gsn PE=1 SV=3	564.3	1100.6	12	24	23.1	42.6	4.43	4.14	18	13
HA10_MOUSE	H-2 class I histocompatibility antigen, Q10 alpha chain OS=Mus musculus GN=H2-Q10 PE=1 SV=3	328.0	725.9	6	14	22.2	44.3	3.08	4.72	32	24
HBB1_MOUSE	Hemoglobin subunit beta-1 OS=Mus musculus GN=Hbb-b1 PE=1 SV=2	0	89.5	0	3	0	30.6	0	8.11	0	103
HEMO_MOUSE	Hemopexin OS=Mus musculus GN=Hpx PE=1 SV=2	438.2	1060.2	10	22	25.4	50.9	5.58	4.10	23	14
HEPC_MOUSE	Hepcidin OS=Mus musculus GN=Hamp PE=2 SV=1	0	50.7	0	1	0	14.5	0	0.45	0	122
HPT_MOUSE	Haptoglobin OS=Mus musculus GN=Hp PE=1 SV=1	83.5	140.3	2	4	9.2	15.9	1.55	4.73	91	89

HRG_MOUSE	Histidine-rich glycoprotein OS=Mus musculus GN=Hrg PE=1 SV=2	589.8	666.7	14	13	26.7	29.3	5.44	4.31	15	26
HVM00_MOUSE	Ig heavy chain V region OS=Mus musculus PE=1 SV=1	47.8	40.5	1	1	11.4	11.4	1.96	7.51	107	124
HVM05_MOUSE	Ig heavy chain V region 3 OS=Mus musculus GN=Ighv1-61 PE=1 SV=1	123.9	111.6	2	2	26.5	26.5	6.26	4.37	76	96
HVM06_MOUSE	Ig heavy chain V region 102 OS=Mus musculus PE=1 SV=1	140.6	151.0	2	2	18.8	18.8	7.51	4.82	69	83
HVM13_MOUSE	Ig heavy chain V region J558 OS=Mus musculus PE=1 SV=1	0	216.2	0	3	0	42.7	0	6.81	0	69
HVM16_MOUSE	Ig heavy chain V region MOPC 21 (Fragment) OS=Mus musculus PE=1 SV=1	81.9	226.3	2	4	11.8	26.5	4.85	4.54	93	67
HVM17_MOUSE	Ig heavy chain V region MOPC 47A OS=Mus musculus PE=1 SV=1	55.9	224.2	2	4	12.0	35.0	3.42	2.21	104	68
HVM21_MOUSE	Ig heavy chain V region M511 OS=Mus musculus PE=1 SV=1	0	437.8	0	7	0	71.3	0	2.65	0	40
HVM32_MOUSE	Ig heavy chain V-III region J606 OS=Mus musculus PE=1 SV=1	0	261.3	0	6	0	49.6	0	2.63	0	61
HVM37_MOUSE	Ig heavy chain V region X44 OS=Mus musculus PE=1 SV=1	0	392.6	0	8	0	68.1	0	5.38	0	42
HVM45_MOUSE	Ig heavy chain V region MC101 OS=Mus musculus PE=1 SV=1	0	52.2	0	2	0	33.6	0	6.33	0	120
HVM51_MOUSE	Ig heavy chain V region AC38 205.12 OS=Mus musculus PE=1 SV=1	474.8	289.9	5	3	63.6	33.9	4.82	4.28	21	53
HVM53_MOUSE	Ig heavy chain V region RF OS=Mus musculus PE=1 SV=1	0	117.6	0	3	0	23.1	0	6	0	93
HVM54_MOUSE	Ig heavy chain V region 5-84 OS=Mus musculus PE=1 SV=1	0	148.1	0	4	0	36.8	0	4.58	0	84
HVM56_MOUSE	Ig heavy chain V region 914 OS=Mus musculus PE=1 SV=1	0	93.7	0	3	0	33	0	2.67	0	99
HVM57_MOUSE	Ig heavy chain V region 6.96 OS=Mus musculus PE=4 SV=1	168.0	86.1	3	2	24.5	24.5	4.23	5.77	62	105
HVM60_MOUSE	Ig heavy chain V region 3-6 OS=Mus musculus GN=Ighv3-6 PE=1 SV=1	106.9	92.6	1	1	13.8	13.8	0.17	0.23	82	100
IBP2_MOUSE	Insulin-like growth factor-binding protein 2 OS=Mus musculus GN=Igfbp2 PE=2 SV=2	82.5	84.2	2	3	9.5	16.1	3.78	3.88	92	109
IBP4_MOUSE	Insulin-like growth factor-binding protein 4 OS=Mus musculus GN=Igfbp4 PE=1 SV=2	0	85	0	3	0	16.5	0	5.59	0	106
IC1_MOUSE	Plasma protease C1 inhibitor OS=Mus musculus GN=Serping1 PE=1 SV=3	147.8	284.6	3	5	10.3	14.1	4.05	6.17	67	54
IGG2B_MOUSE	Ig gamma-2B chain C region OS=Mus musculus GN=Igh-3 PE=1 SV=3	173.5	294.2	5	6	15.8	22.8	4.80	2.90	60	52
IGHG1_MOUSE	Ig gamma-1 chain C region secreted form OS=Mus musculus GN=Ighg1 PE=1 SV=1	57.6	309.2	1	6	10.5	36.1	1.38	5.06	102	49
IGHG3_MOUSE	Ig gamma-3 chain C region OS=Mus musculus PE=1 SV=2	322.0	812.0	5	14	16.3	47.0	4.42	4.88	33	20
IGHM_MOUSE	Ig mu chain C region OS=Mus musculus GN=Ighm PE=1 SV=2	2154.1	2512.5	31	38	66.7	69.6	3.66	3.03	3	3
IGJ_MOUSE	Immunoglobulin J chain OS=Mus musculus GN=Jchain PE=1 SV=4	543.8	579.3	11	11	59.1	59.1	4.04	4.10	19	30
IGKC_MOUSE	Ig kappa chain C region OS=Mus musculus PE=1 SV=1	1183.7	1759.2	13	16	78.3	78.3	4.44	3.75	7	6
ITIH4_MOUSE	Inter alpha-trypsin inhibitor, heavy chain 4 OS=Mus musculus GN=Itih4 PE=1 SV=2	706.9	1445.4	14	26	21.1	45.5	5.77	4.35	11	8
KNG1_MOUSE	Kininogen-1 OS=Mus musculus GN=Kng1 PE=1 SV=1	566.8	623.2	10	11	18.9	21.3	5.04	4.83	17	27
KV2A5_MOUSE	Ig kappa chain V-II region 17S29.1 OS=Mus musculus PE=1 SV=1	0	40	0	1	0	11.5	0	3.18	0	125
KV2A6_MOUSE	Ig kappa chain V-II region 7S34.1 OS=Mus musculus PE=1 SV=1	114.6	156.7	2	2	21.2	21.2	5.43	6.81	78	79
KV2A7_MOUSE	Ig kappa chain V-II region 26-10 OS=Mus musculus PE=1 SV=1	203.9	299.5	3	4	25.7	43.4	4.23	2.67	53	51
KV3A4_MOUSE	Ig kappa chain V-III region 50S10.1 OS=Mus musculus PE=1 SV=1	256.6	228.9	3	3	40.5	40.5	5.06	1.58	43	65
KV3A8_MOUSE	Ig kappa chain V-III region PC 3741/TEPC 111 OS=Mus musculus PE=1 SV=1	258.0	194.8	4	3	53.2	30.6	4.43	4.52	42	76
KV3AB_MOUSE	Ig kappa chain V-III region PC 4050 OS=Mus musculus PE=1 SV=1	274.8	260.3	4	3	53.2	30.6	5.65	5.39	39	62
KV3AG_MOUSE	Ig kappa chain V-III region PC 7210 OS=Mus musculus PE=1 SV=1	0	203.8	0	2	0	30.9	0	1.51	0	75
KV3AI_MOUSE	Ig kappa chain V-III region PC 6684 OS=Mus musculus PE=1 SV=1	253.3	474.0	4	5	68.5	88.3	5.24	4.82	45	37
KV3AJ_MOUSE	Ig kappa chain V-III region PC 7175 OS=Mus musculus PE=1 SV=1	0	522.1	0	5	0	88.3	0	5.6	0	32
KV3AM_MOUSE	Ig kappa chain V-III region PC 2154 OS=Mus musculus PE=1 SV=1	90.9	80.1	1	1	11.1	11.1	5.29	3.25	87	111
KV4A1_MOUSE	Ig kappa chain V-IV region S107B OS=Mus musculus PE=4 SV=1	62.4	61.6	1	1	8.5	8.5	7.47	2.24	100	115
KV5A1_MOUSE	Ig kappa chain V19-17 OS=Mus musculus GN=Igk-V19-17 PE=1 SV=1	173.3	178.0	4	4	16.8	16.1	4.03	5.33	61	77
KV5A3_MOUSE	Ig kappa chain V-V region K2 (Fragment) OS=Mus musculus PE=1 SV=1	220.6	206.8	4	4	40.9	40.9	6.62	1.41	52	74
KV5A4_MOUSE	Ig kappa chain V-V region MOPC 149 OS=Mus musculus PE=1 SV=1	262.6	228.8	2	2	25.9	25.9	7.21	1.28	41	66
KV5A6_MOUSE	Ig kappa chain V-V region L6 (Fragment) OS=Mus musculus PE=4 SV=1	188.5	210.4	3	3	35.7	35.7	3.26	4.80	55	72
KV5A7_MOUSE	Ig kappa chain V-V region MOPC 41 OS=Mus musculus GN=Gm5571 PE=1 SV=1	201.2	334.4	3	4	28.5	28.5	5.17	5.89	54	45
KV5A9_MOUSE	Ig kappa chain V-V region L7 (Fragment) OS=Mus musculus GN=Gm10881 PE=1 SV=1	72.8	65.5	1	2	13.0	18.3	2.11	1.21	97	114
KV5AB_MOUSE	Ig kappa chain V-V region HP R16.7 OS=Mus musculus PE=1 SV=1	0	276.9	0	5	0	59.3	0	4.97	0	57
KV5AG_MOUSE	Ig kappa chain V-V regions OS=Mus musculus PE=1 SV=1	0	99.5	0	3	0	20.4	0	5.09	0	98
KV6A5_MOUSE	Ig kappa chain V-VI region J539 OS=Mus musculus PE=1 SV=1	0	84.2	0	2	0	24.3	0	4.83	0	108
KV6A7_MOUSE	Ig kappa chain V-VI region NQ2-48.2.2 OS=Mus musculus PE=2 SV=1	113.6	75.4	3	2	32.7	14.0	5.80	3.64	80	113
KV6AB_MOUSE	Ig kappa chain V-VI region NQ2-6.1 OS=Mus musculus PE=2 SV=1	137.8	133.4	1	1	14.8	14.8	9.31	4.37	70	90
LOXL1_MOUSE	Lysyl oxidase homolog 1 OS=Mus musculus GN=Loxl1 PE=2 SV=3	270.4	145.5	6	3	18.6	7.9	3.49	1.65	40	86
LV1B_MOUSE	Ig lambda-1 chain V regions MOPC 104E/RPC20/J558/S104 OS=Mus musculus PE=1 SV=1	0	83.8	0	2	0	32.6	0	7.2	0	110

MASP1_MOUSE	Mannan-binding lectin serine protease 1 OS=Mus musculus GN=Masp1 PE=1 SV=2	231.9	214.3	5	5	8.4	8.8	3.25	3.59	51	70
MASP2_MOUSE	Mannan-binding lectin serine protease 2 OS=Mus musculus GN=Masp2 PE=1 SV=1	381.2	237.2	7	6	14.5	12.8	5.33	3.77	28	63
MBL1_MOUSE	Mannose-binding protein A OS=Mus musculus GN=Mbl1 PE=1 SV=1	291.0	146.3	5	3	28.9	13.4	3.29	3.05	37	85
MBL2_MOUSE	Mannose-binding protein C OS=Mus musculus GN=Mbl2 PE=1 SV=2	584.7	598.7	10	10	48.0	44.7	4.38	4.49	16	29
MUG1_MOUSE	Murinoglobulin-1 OS=Mus musculus GN=Mug1 PE=1 SV=3	88.0	458.0	2	9	1.7	10.7	8.59	5.56	89	39
MUP1_MOUSE	Major urinary protein 1 OS=Mus musculus GN=Mup1 PE=1 SV=1	0	497.2	0	8	0	64.4	0	4.86	0	34
MUP2_MOUSE	Major urinary protein 2 OS=Mus musculus GN=Mup2 PE=1 SV=1	44.1	470.9	1	8	6.1	62.8	1.69	4.78	110	38
NPY_MOUSE	Pro-neuropeptide Y OS=Mus musculus GN=Npy PE=1 SV=2	0	76.1	0	2	0	21.6	0	3.65	0	112
PLMN_MOUSE	Plasminogen OS=Mus musculus GN=Plg PE=1 SV=3	125.0	207.0	3	5	6.9	6.9	6.18	4.66	74	73
PROP_MOUSE	Properdin OS=Mus musculus GN=Cfp PE=2 SV=2	250.8	278.0	6	6	15.7	19.6	4.12	2.47	47	56
PZP_MOUSE	Pregnancy zone protein OS=Mus musculus GN=Pzp PE=1 SV=3	408.7	732.7	9	20	10.1	22.0	4.53	4.61	25	23
QSOX1_MOUSE	Sulfhydryl oxidase 1 OS=Mus musculus GN=Qsox1 PE=1 SV=1	0	163.1	0	4	0	5.6	0	3.16	0	78
RET4_MOUSE	Retinol-binding protein 4 OS=Mus musculus GN=Rbp4 PE=1 SV=2	164.5	152.8	3	3	18.9	24.9	6.90	2.25	63	82
SAA1_MOUSE	Serum amyloid A-1 protein OS=Mus musculus GN=Saa1 PE=1 SV=2	98.9	130.1	3	2	19.7	15.6	6.46	8.47	83	91
SAMP_MOUSE	Serum amyloid P-component OS=Mus musculus GN=Apcs PE=1 SV=2	0	117.4	0	4	0	20.1	0	4.07	0	94
SBSN_MOUSE	Suprabasin OS=Mus musculus GN=Sbsn PE=2 SV=1	0	61.5	0	2	0	4	0	3.25	0	116
SEPP1_MOUSE	Selenoprotein P OS=Mus musculus GN=Sepp1 PE=1 SV=3	47.5	86.9	1	3	5.8	14.7	2.56	3.45	108	104
SPA3K_MOUSE	Serine protease inhibitor A3K OS=Mus musculus GN=Serpina3k PE=1 SV=2	234.6	823.0	7	14	28.7	41.9	4.32	4.07	50	18
TETN_MOUSE	Tetranectin OS=Mus musculus GN=Clec3b PE=1 SV=2	71.3	319.4	1	6	16.3	52.0	2.52	2.85	98	47
THRB_MOUSE	Prothrombin OS=Mus musculus GN=F2 PE=1 SV=1	303.8	682.4	6	12	13.8	19.7	3.38	4.35	35	25
TRFE_MOUSE	Serotransferrin OS=Mus musculus GN=Tf PE=1 SV=1	496.9	1368.7	9	25	15.6	45.3	5.40	4.48	20	9
TSP1_MOUSE	Thrombospondin-1 OS=Mus musculus GN=Thbs1 PE=1 SV=1	666.7	1965.0	17	41	16.3	43.8	4.67	3.80	12	5
TTHY_MOUSE	Transthyretin OS=Mus musculus GN=Ttr PE=1 SV=1	373.7	522.9	7	9	62.6	66.7	3.31	2.74	29	31
VTDB_MOUSE	Vitamin D-binding protein OS=Mus musculus GN=Gc PE=1 SV=2	175.7	480.2	5	10	17.0	37.4	4.37	3.96	59	35
VTNC_MOUSE	Vitronectin OS=Mus musculus GN=Vtn PE=1 SV=2	0	235.9	0	5	0	18.8	0	2.79	0	64

2h Ball Milled SWCNTs

Accession	Name	Meta Score A	Meta Score B	Peptides A	Peptides B	SC [%] A	SC [%] B	RMS90 [ppm] A	RMS90 [ppm] B	Rank A	Rank B
A1AG1_MOUSE	Alpha-1-acid glycoprotein 1 OS=Mus musculus GN=Orm1 PE=1 SV=1	55.9	89.1	1	1	7.2	7.2	0.44	0.63	100	88
A1AT2_MOUSE	Alpha-1-antitrypsin 1-2 OS=Mus musculus GN=Serpina1b PE=1 SV=2	90.8	0	1	0	1.9	0	5.36	0	81	0
A1AT3_MOUSE	Alpha-1-antitrypsin 1-3 OS=Mus musculus GN=Serpina1c PE=1 SV=2	103.8	176.8	2	6	4.4	20.4	5.41	3.3	78	58
A1AT4_MOUSE	Alpha-1-antitrypsin 1-4 OS=Mus musculus GN=Serpina1d PE=1 SV=1	115.7	155.3	2	5	4.4	18.2	7.89	4.78	73	65
A2AP_MOUSE	Alpha-2-antiplasmin OS=Mus musculus GN=Serpinf2 PE=1 SV=1	42.9	0	1	0	1.6	0	5.14	0	105	0
FETUA_MOUSE	Alpha-2-HS-glycoprotein OS=Mus musculus GN=Ahsg PE=1 SV=1	632.4	988.8	9	12	33.9	35.9	3.52	2.97	15	9
APOA1_MOUSE	Apolipoprotein A-I OS=Mus musculus GN=Apoa1 PE=1 SV=2	858.7	1201	17	21	48.1	69.3	4.01	4.19	9	6
APOA2_MOUSE	Apolipoprotein A-II OS=Mus musculus GN=Apoa2 PE=1 SV=2	153.9	124.5	4	2	37.3	32.4	3.04	2.64	60	75
APOA4_MOUSE	Apolipoprotein A-IV OS=Mus musculus GN=Apoa4 PE=1 SV=3	656.2	935.3	16	15	57	50.6	3.73	3.91	11	10
APOB_MOUSE	Apolipoprotein B-100 OS=Mus musculus GN=Apob PE=1 SV=1	109.6	0	3	0	0.8	0	6.52	0	75	0
APOC1_MOUSE	Apolipoprotein C-I OS=Mus musculus GN=Apoc1 PE=1 SV=1	183.3	153.9	4	3	25	22.7	6.02	6.3	53	66
APOC2_MOUSE	Apolipoprotein C-II OS=Mus musculus GN=Apoc2 PE=2 SV=1	182.2	283.1	3	4	30.9	33	3.3	1.59	54	38
APOC3_MOUSE	Apolipoprotein C-III OS=Mus musculus GN=Apoc3 PE=1 SV=2	373.9	398.1	4	5	50.5	50.5	2.31	4.58	28	27
APOE_MOUSE	Apolipoprotein E OS=Mus musculus GN=Apoe PE=1 SV=2	1124.8	1087.7	22	21	62.1	50.2	3.44	3.59	7	8
APOH_MOUSE	Beta-2-glycoprotein 1 OS=Mus musculus GN=Apoh PE=1 SV=1	172.8	330.3	4	9	16.8	29	6.07	4.17	58	31
B2MG_MOUSE	Beta-2-microglobulin OS=Mus musculus GN=B2m PE=1 SV=2	71.2	58.5	1	1	7.6	7.6	6.04	4.06	90	101
ACTBL_MOUSE	Beta-actin-like protein 2 OS=Mus musculus GN=Actbl2 PE=1 SV=1	85.2	0	2	0	9	0	5.14	0	83	0
CRP_MOUSE	C-reactive protein OS=Mus musculus GN=Crp PE=1 SV=2	491.9	239.7	9	6	42.2	38.2	2.79	2.1	20	49
C4BPA_MOUSE	C4b-binding protein OS=Mus musculus GN=C4bpa PE=1 SV=3	121.8	405.3	2	7	6	19.8	1.92	3.16	70	26
CD5L_MOUSE	CD5 antigen-like OS=Mus musculus GN=Cd5l PE=1 SV=3	1198.2	1354.7	16	23	47.7	60.5	4.26	3.82	5	5
CLUS_MOUSE	Clusterin OS=Mus musculus GN=Clu PE=1 SV=1	406.7	653.4	8	13	25.4	31.7	5.05	4.81	27	13
C1QA_MOUSE	Complement C1q subcomponent subunit A OS=Mus musculus GN=C1qa PE=1 SV=2	189.2	129	4	2	20	14.3	4.77	3.49	52	73
C1QB_MOUSE	Complement C1q subcomponent subunit B OS=Mus musculus GN=C1qb PE=1 SV=2	356.6	433.2	6	7	27.3	36	3.03	3.98	29	24

C1QC_MOUSE	Complement C1q subcomponent subunit C OS=Mus musculus GN=C1qc PE=1 SV=2	148.8	94.3	2	1	12.6	7.3	2.02	5.42	63	86
C1RA_MOUSE	Complement C1r-A subcomponent OS=Mus musculus GN=C1ra PE=1 SV=1	425.6	621	11	13	29.7	34.8	3.76	4.05	25	14
CS1A_MOUSE	Complement C1s-A subcomponent OS=Mus musculus GN=C1sa PE=2 SV=2	434.8	298.8	8	5	21.7	12.1	3.59	5.3	24	36
CO3_MOUSE	Complement C3 OS=Mus musculus GN=C3 PE=1 SV=3	2722.7	3021.5	55	60	44.8	43.4	3.47	4.69	1	1
CO4B_MOUSE	Complement C4-B OS=Mus musculus GN=C4b PE=1 SV=3	419.3	464.2	9	11	9.4	10.2	3.01	4.13	26	22
CFAH_MOUSE	Complement factor H OS=Mus musculus GN=Cfh PE=1 SV=2	459	1599.5	11	32	14.6	39.7	2.77	5.38	23	4
FIBA_MOUSE	Fibrinogen alpha chain OS=Mus musculus GN=Fga PE=1 SV=1	468	240.4	10	4	20.3	8.4	3.67	6.87	22	48
FIBB_MOUSE	Fibrinogen beta chain OS=Mus musculus GN=Fgb PE=1 SV=1	646.9	0	16	0	47.8	0	2.73	0	12	0
FIBG_MOUSE	Fibrinogen gamma chain OS=Mus musculus GN=Fgg PE=1 SV=1	637.5	135.4	12	4	45.2	15.4	2.53	3.13	14	72
FINC_MOUSE	Fibronectin OS=Mus musculus GN=Fn1 PE=1 SV=4	1164.1	346.1	27	8	17.6	5.4	4.47	4.41	6	30
GELS_MOUSE	Gelsolin OS=Mus musculus GN=Gsn PE=1 SV=3	505.7	564.3	14	12	31	23.1	4.07	4.43	19	18
G3P_MOUSE	Glyceraldehyde-3-phosphate dehydrogenase OS=Mus musculus GN=Gapdh PE=1 SV=2	56.6	0	2	0	7.8	0	1.93	0	99	0
HA10_MOUSE	H-2 class I histocompatibility antigen, Q10 alpha chain OS=Mus musculus GN=H2-Q10 PE=1 SV=3	232.9	328	6	6	23.1	22.2	5.08	3.08	42	32
HBA_MOUSE	Hemoglobin subunit alpha OS=Mus musculus GN=Hba PE=1 SV=2	202.7	114.3	4	2	33.1	16.9	5.46	2.57	50	79
HBB1_MOUSE	Hemoglobin subunit beta-1 OS=Mus musculus GN=Hbb-b1 PE=1 SV=2	68.8	0	2	0	19.7	0	5.91	0	92	0
HEMO_MOUSE	Hemopexin OS=Mus musculus GN=Hpx PE=1 SV=2	613	438.2	15	10	37.2	25.4	4.35	5.58	16	23
HRG_MOUSE	Histidine-rich glycoprotein OS=Mus musculus GN=Hrg PE=1 SV=2	215.5	589.8	7	14	11	26.7	2.69	5.44	47	15
GCAB_MOUSE	Ig gamma-2A chain C region secreted form OS=Mus musculus PE=1 SV=1	75.8	181.4	2	3	7.5	12.2	4.31	3.38	85	56
IGG2B_MOUSE	Ig gamma-2B chain C region OS=Mus musculus GN=Igh-3 PE=1 SV=3	138.2	173.5	3	5	10.4	15.8	5.32	4.8	66	60
IGHG3_MOUSE	Ig gamma-3 chain C region OS=Mus musculus PE=1 SV=2	642.6	322	10	5	38.4	16.3	4.95	4.42	13	33
HVM10_MOUSE	Ig heavy chain V region 1-62-3 OS=Mus musculus GN=Ighv1-62-3 PE=4 SV=2	143.4	0	3	0	45.3	0	4.11	0	64	0
HVM06_MOUSE	Ig heavy chain V region 102 OS=Mus musculus PE=1 SV=1	118.6	140.6	2	2	23.1	18.8	3.2	7.51	71	69
HVM14_MOUSE	Ig heavy chain V region 108A OS=Mus musculus GN=Igh-VJ558 PE=4 SV=1	60.9	73.4	1	1	12.8	12.8	4.74	3.24	97	96
HVM05_MOUSE	Ig heavy chain V region 3 OS=Mus musculus GN=Ighv1-61 PE=1 SV=1	61.9	123.9	1	2	20.5	26.5	0.92	6.26	96	76
HVM60_MOUSE	Ig heavy chain V region 3-6 OS=Mus musculus GN=Ighv3-6 PE=1 SV=1	153.2	106.9	1	1	13.8	13.8	1.56	0.17	62	82
HVM03_MOUSE	Ig heavy chain V region 36-65 OS=Mus musculus PE=1 SV=1	174	160.7	3	3	20	20	4.56	2.87	56	64
HVM36_MOUSE	Ig heavy chain V region 441 OS=Mus musculus PE=4 SV=1	73.8	95.6	2	3	12.1	19.8	1.81	5.28	87	85
HVM57_MOUSE	Ig heavy chain V region 6.96 OS=Mus musculus PE=4 SV=1	101.7	168	1	3	19.4	24.5	4.38	4.23	79	62
HVM51_MOUSE	Ig heavy chain V region AC38 205.12 OS=Mus musculus PE=1 SV=1	482	474.8	5	5	72	63.6	4.26	4.82	21	21
HVM16_MOUSE	Ig heavy chain V region MOPC 21 (Fragment) OS=Mus musculus PE=1 SV=1	53.9	81.9	1	2	8.1	11.8	8.55	4.85	101	93
HVM52_MOUSE	Ig heavy chain V region VH558 A1/A4 OS=Mus musculus GN=Gm5629 PE=2 SV=1	74.2	44.4	1	1	20.5	20.5	1.43	4.16	86	109
HVM28_MOUSE	Ig heavy chain V-III region U61 OS=Mus musculus PE=1 SV=1	73.8	0	1	0	8	0	5.74	0	88	0
IGKC_MOUSE	Ig kappa chain C region OS=Mus musculus PE=1 SV=1	1550.8	1183.7	13	13	70.8	78.3	1.93	4.44	4	7
KV2A7_MOUSE	Ig kappa chain V-II region 26-10 OS=Mus musculus PE=1 SV=1	176.7	203.9	2	3	32.7	25.7	2.05	4.23	55	53
KV2A6_MOUSE	Ig kappa chain V-II region 7S34.1 OS=Mus musculus PE=1 SV=1	129.3	114.6	2	2	21.2	21.2	1.78	5.43	68	78
KV3A4_MOUSE	Ig kappa chain V-III region 50S10.1 OS=Mus musculus PE=1 SV=1	234.8	256.6	3	3	40.5	40.5	6.31	5.06	41	43
KV3AC_MOUSE	Ig kappa chain V-III region CBPC 101 OS=Mus musculus PE=1 SV=1	237	253.2	3	3	68.5	68.5	4.26	4.13	40	46
KV3AM_MOUSE	Ig kappa chain V-III region PC 2154 OS=Mus musculus PE=1 SV=1	110.6	90.9	1	1	11.1	11.1	5.04	5.29	74	87
KV3A1_MOUSE	Ig kappa chain V-III region PC 2880/PC 1229 OS=Mus musculus PE=1 SV=1	269.5	306.2	3	4	53.2	63.1	6.09	6.17	33	34
KV3A8_MOUSE	Ig kappa chain V-III region PC 3741/TEPC 111 OS=Mus musculus PE=1 SV=1	217.6	258	3	4	30.6	53.2	3.29	4.43	45	42
KV3AI_MOUSE	Ig kappa chain V-III region PC 6684 OS=Mus musculus PE=1 SV=1	269.4	253.3	4	4	68.5	68.5	2.84	5.24	34	45
KV3AE_MOUSE	Ig kappa chain V-III region PC 7183 OS=Mus musculus PE=1 SV=1	246.1	0	3	0	53.2	0	3.24	0	38	0
KV4A1_MOUSE	Ig kappa chain V-IV region S107B OS=Mus musculus PE=4 SV=1	51.5	62.4	1	1	8.5	8.5	3.34	7.47	102	100
KV5AB_MOUSE	Ig kappa chain V-V region HP R16.7 OS=Mus musculus PE=1 SV=1	217.1	0	4	0	53.7	0	3.42	0	46	0
KV5A3_MOUSE	Ig kappa chain V-V region K2 (Fragment) OS=Mus musculus PE=1 SV=1	261.7	220.6	4	4	40.9	40.9	3.78	6.62	35	52
KV5A6_MOUSE	Ig kappa chain V-V region L6 (Fragment) OS=Mus musculus PE=4 SV=1	203.9	188.5	4	3	35.7	35.7	3.87	3.26	49	55
KV5A9_MOUSE	Ig kappa chain V-V region L7 (Fragment) OS=Mus musculus GN=Gm10881 PE=1 SV=1	68.8	72.8	2	1	23.5	13	5.54	2.11	93	97
KV5A4_MOUSE	Ig kappa chain V-V region MOPC 149 OS=Mus musculus PE=1 SV=1	245.6	262.6	2	2	25.9	25.9	2.1	7.21	39	41
KV5A7_MOUSE	Ig kappa chain V-V region MOPC 41 OS=Mus musculus GN=Gm5571 PE=1 SV=1	153.3	201.2	3	3	28.5	28.5	5.1	5.17	61	54
KV6A7_MOUSE	Ig kappa chain V-VI region NQ2-48.2.2 OS=Mus musculus PE=2 SV=1	66.7	113.6	2	3	25.2	32.7	4.13	5.8	94	80
KV6AB_MOUSE	Ig kappa chain V-VI region NQ2-6.1 OS=Mus musculus PE=2 SV=1	116	137.8	1	1	14.8	14.8	3.1	9.31	72	70
KV5A1_MOUSE	Ig kappa chain V19-17 OS=Mus musculus GN=Igk-V19-17 PE=1 SV=1	105.2	173.3	2	4	12.1	16.8	6.1	4.03	77	61

LAC1_MOUSE	Ig lambda-1 chain C region OS=Mus musculus PE=1 SV=1	202	97.4	3	2	46.7	35.2	1.89	4.89	51	84
LV1C_MOUSE	Ig lambda-1 chain V region S178 OS=Mus musculus PE=1 SV=1	47.5	0	1	0	8.2	0	4.23	0	104	0
IGHM_MOUSE	Ig mu chain C region OS=Mus musculus GN=Ighm PE=1 SV=2	2313.3	2154.1	36	31	70.9	66.7	3.56	3.66	3	3
IGJ_MOUSE	Immunoglobulin J chain OS=Mus musculus GN=Jchain PE=1 SV=4	529.7	543.8	10	11	59.1	59.1	4.07	4.04	18	19
ITIH4_MOUSE	Inter alpha-trypsin inhibitor, heavy chain 4 OS=Mus musculus GN=Itih4 PE=1 SV=2	330.3	706.9	7	14	9.7	21.1	4.74	5.77	31	11
K1C10_MOUSE	Keratin, type I cytoskeletal 10 OS=Mus musculus GN=Krt10 PE=1 SV=3	212.7	0	4	0	7.5	0	3.77	0	48	0
K2C1_MOUSE	Keratin, type II cytoskeletal 1 OS=Mus musculus GN=Krt1 PE=1 SV=4	72	0	2	0	3.6	0	4.83	0	89	0
K22E_MOUSE	Keratin, type II cytoskeletal 2 epidermal OS=Mus musculus GN=Krt2 PE=1 SV=1	62.9	53.6	1	1	2	2	3.84	8.35	95	105
K2C6A_MOUSE	Keratin, type II cytoskeletal 6A OS=Mus musculus GN=Krt6a PE=1 SV=3	71.1	0	1	0	2.2	0	5.61	0	91	0
KNG1_MOUSE	Kininogen-1 OS=Mus musculus GN=Kng1 PE=1 SV=1	89.7	566.8	3	10	5	18.9	3.07	5.04	82	17
LBP_MOUSE	Lipopolysaccharide-binding protein OS=Mus musculus GN=Lbp PE=1 SV=2	105.7	0	4	0	11.6	0	4.71	0	76	0
LOXL1_MOUSE	Lysyl oxidase homolog 1 OS=Mus musculus GN=Loxl1 PE=2 SV=3	94.3	270.4	2	6	4.8	18.6	5.57	3.49	80	40
MASP1_MOUSE	Mannan-binding lectin serine protease 1 OS=Mus musculus GN=Masp1 PE=1 SV=2	162.3	231.9	6	5	10.1	8.4	3.25	3.25	59	51
MASP2_MOUSE	Mannan-binding lectin serine protease 2 OS=Mus musculus GN=Masp2 PE=1 SV=1	221.8	381.2	6	7	12.6	14.5	3.99	5.33	44	28
MBL1_MOUSE	Mannose-binding protein A OS=Mus musculus GN=Mbl1 PE=1 SV=1	140.4	291	2	5	9.6	28.9	2.86	3.29	65	37
MBL2_MOUSE	Mannose-binding protein C OS=Mus musculus GN=Mbl2 PE=1 SV=2	589.3	584.7	9	10	41.8	48	3.91	4.38	17	16
PRDX2_MOUSE	Peroxiredoxin-2 OS=Mus musculus GN=Prdx2 PE=1 SV=3	75.9	0	1	0	9.1	0	2.38	0	84	0
PLTP_MOUSE	Phospholipid transfer protein OS=Mus musculus GN=Pltp PE=1 SV=1	49.5	0	1	0	2.4	0	6.23	0	103	0
IC1_MOUSE	Plasma protease C1 inhibitor OS=Mus musculus GN=Serping1 PE=1 SV=3	130.4	147.8	3	3	10.3	10.3	4.46	4.05	67	67
PLMN_MOUSE	Plasminogen OS=Mus musculus GN=Plg PE=1 SV=3	124.9	125	3	3	5.2	6.9	5.04	6.18	69	74
PZP_MOUSE	Pregnancy zone protein OS=Mus musculus GN=Pzp PE=1 SV=3	348.4	408.7	9	9	9.9	10.1	2.81	4.53	30	25
PROP_MOUSE	Properdin OS=Mus musculus GN=Cfp PE=2 SV=2	259.8	250.8	5	6	17.7	15.7	3.46	4.12	36	47
THRB_MOUSE	Prothrombin OS=Mus musculus GN=F2 PE=1 SV=1	224.6	303.8	6	6	11.7	13.8	3.13	3.38	43	35
SPA3K_MOUSE	Serine protease inhibitor A3K OS=Mus musculus GN=Serpina3k PE=1 SV=2	174	234.6	5	7	19.6	28.7	5.52	4.32	57	50
TRFE_MOUSE	Serotransferrin OS=Mus musculus GN=Tf PE=1 SV=1	934.7	496.9	21	9	38.5	15.6	3.3	5.4	8	20
ALBU_MOUSE	Serum albumin OS=Mus musculus GN=Alb PE=1 SV=3	2610.1	2371.7	39	37	66.8	74.2	3.57	4.3	2	2
SPTA1_MOUSE	Spectrin alpha chain, erythrocytic 1 OS=Mus musculus GN=Spta1 PE=1 SV=3	57.7	0	1	0	0.8	0	4.92	0	98	0
TSP1_MOUSE	Thrombospondin-1 OS=Mus musculus GN=Thbs1 PE=1 SV=1	658.9	666.7	15	17	19.5	16.3	4.41	4.67	10	12
TTHY_MOUSE	Transthyretin OS=Mus musculus GN=Ttr PE=1 SV=1	249.8	373.7	6	7	62.6	62.6	2.86	3.31	37	29
VTDB_MOUSE	Vitamin D-binding protein OS=Mus musculus GN=Gc PE=1 SV=2	288.7	175.7	8	5	26.1	17	3.53	4.37	32	59

4h Ball Milled SWCNTs

Accession	Name	Meta Score A	Meta Score B	Peptides A	Peptides B	SC [%] A	SC [%] B	RMS90 [ppm] A	RMS90 [ppm] B	Rank A	Rank B
A1AT2_MOUSE	Alpha-1-antitrypsin 1-2 OS=Mus musculus GN=Serpina1b PE=1 SV=2	109.9	0	2	0	7.3	0	1.01	0	61	0
A1AT3_MOUSE	Alpha-1-antitrypsin 1-3 OS=Mus musculus GN=Serpina1c PE=1 SV=2	102.9	176.8	3	6	9.2	20.4	0.97	3.3	65	58
A1AT4_MOUSE	Alpha-1-antitrypsin 1-4 OS=Mus musculus GN=Serpina1d PE=1 SV=1	101.8	155.3	2	5	4.4	18.2	0.82	4.78	66	65
FETUA_MOUSE	Alpha-2-HS-glycoprotein OS=Mus musculus GN=Ahsg PE=1 SV=1	494.5	988.8	7	12	31.6	35.9	2.24	2.97	12	9
APOA1_MOUSE	Apolipoprotein A-I OS=Mus musculus GN=Apoa1 PE=1 SV=2	628.7	1201	12	21	43.2	69.3	2.82	4.19	8	6
APOA2_MOUSE	Apolipoprotein A-II OS=Mus musculus GN=Apoa2 PE=1 SV=2	82.8	124.5	2	2	14.7	32.4	2.02	2.64	77	75
APOA4_MOUSE	Apolipoprotein A-IV OS=Mus musculus GN=Apoa4 PE=1 SV=3	357	935.3	10	15	30.9	50.6	5.18	3.91	23	10
APOB_MOUSE	Apolipoprotein B-100 OS=Mus musculus GN=Apob PE=1 SV=1	72.9	0	3	0	1.2	0	5.82	0	81	0
APOC1_MOUSE	Apolipoprotein C-I OS=Mus musculus GN=Apoc1 PE=1 SV=1	154.5	153.9	3	3	22.7	22.7	6.07	6.3	49	66
APOC2_MOUSE	Apolipoprotein C-II OS=Mus musculus GN=Apoc2 PE=2 SV=1	144.2	283.1	3	4	30.9	33	3.92	1.59	52	38
APOC3_MOUSE	Apolipoprotein C-III OS=Mus musculus GN=Apoc3 PE=1 SV=2	338.2	398.1	4	5	50.5	50.5	4.41	4.58	25	27
APOC4_MOUSE	Apolipoprotein C-IV OS=Mus musculus GN=Apoc4 PE=1 SV=1	50.5	0	1	0	8.9	0	0.38	0	90	0
APOE_MOUSE	Apolipoprotein E OS=Mus musculus GN=Apoe PE=1 SV=2	936.7	1087.7	16	21	41.2	50.2	3.99	3.59	6	8
B2MG_MOUSE	Beta-2-microglobulin OS=Mus musculus GN=B2m PE=1 SV=2	74.8	58.5	2	1	26.9	7.6	2.03	4.06	79	101
CRP_MOUSE	C-reactive protein OS=Mus musculus GN=Crp PE=1 SV=2	313.1	239.7	8	6	41.3	38.2	2.98	2.1	26	49
C4BPA_MOUSE	C4b-binding protein OS=Mus musculus GN=C4bpa PE=1 SV=3	166.8	405.3	4	7	12.4	19.8	6.66	3.16	47	26
CD5L_MOUSE	CD5 antigen-like OS=Mus musculus GN=Cd5l PE=1 SV=3	1071.1	1354.7	12	23	37.2	60.5	4.97	3.82	5	5
CLUS_MOUSE	Clusterin OS=Mus musculus GN=Clu PE=1 SV=1	198	653.4	4	13	16.1	31.7	4.08	4.81	38	13

C1QA_MOUSE	Complement C1q subcomponent subunit A OS=Mus musculus GN=C1qa PE=1 SV=2	128.4	129	4	2	24.5	14.3	6.92	3.49	57	73
C1QB_MOUSE	Complement C1q subcomponent subunit B OS=Mus musculus GN=C1qb PE=1 SV=2	396.8	433.2	6	7	27.3	36	4.92	3.98	18	24
C1QC_MOUSE	Complement C1q subcomponent subunit C OS=Mus musculus GN=C1qc PE=1 SV=2	149.1	94.3	3	1	16.7	7.3	4.13	5.42	51	86
C1RA_MOUSE	Complement C1r-A subcomponent OS=Mus musculus GN=C1ra PE=1 SV=1	431.4	621	9	13	24.5	34.8	5.62	4.05	17	14
CS1A_MOUSE	Complement C1s-A subcomponent OS=Mus musculus GN=C1sa PE=2 SV=2	264.4	298.8	5	5	13.5	12.1	3.69	5.3	28	36
CO3_MOUSE	Complement C3 OS=Mus musculus GN=C3 PE=1 SV=3	1664.4	3021.5	39	60	29.8	43.4	4.32	4.69	3	1
CO4B_MOUSE	Complement C4-B OS=Mus musculus GN=C4b PE=1 SV=3	300.6	464.2	7	11	7.1	10.2	4.4	4.13	27	22
CFAH_MOUSE	Complement factor H OS=Mus musculus GN=Cfh PE=1 SV=2	482.8	1599.5	13	32	16.9	39.7	3.66	5.38	14	4
FIBA_MOUSE	Fibrinogen alpha chain OS=Mus musculus GN=Fga PE=1 SV=1	340.6	240.4	7	4	15.1	8.4	4.63	6.87	24	48
FIBB_MOUSE	Fibrinogen beta chain OS=Mus musculus GN=Fgb PE=1 SV=1	257.6	0	6	0	23.3	0	3.58	0	29	0
FIBG_MOUSE	Fibrinogen gamma chain OS=Mus musculus GN=Fgg PE=1 SV=1	517	135.4	10	4	36.5	15.4	5.83	3.13	10	72
FINC_MOUSE	Fibronectin OS=Mus musculus GN=Fn1 PE=1 SV=4	589.1	346.1	15	8	9.4	5.4	4.71	4.41	9	30
GELS_MOUSE	Gelsolin OS=Mus musculus GN=Gsn PE=1 SV=3	504.7	564.3	10	12	24.9	23.1	5.84	4.43	11	18
HA10_MOUSE	H-2 class I histocompatibility antigen, Q10 alpha chain OS=Mus musculus GN=H2-Q10 PE=1 SV=3	199.9	328	3	6	11.4	22.2	2.68	3.08	37	32
HBA_MOUSE	Hemoglobin subunit alpha OS=Mus musculus GN=Hba PE=1 SV=2	97.5	114.3	2	2	16.9	16.9	3.13	2.57	69	79
HBB1_MOUSE	Hemoglobin subunit beta-1 OS=Mus musculus GN=Hbb-b1 PE=1 SV=2	56.6	0	1	0	6.8	0	9.71	0	88	0
HEMO_MOUSE	Hemopexin OS=Mus musculus GN=Hpx PE=1 SV=2	359.7	438.2	9	10	23.9	25.4	3.47	5.58	22	23
HRG_MOUSE	Histidine-rich glycoprotein OS=Mus musculus GN=Hrg PE=1 SV=2	107.2	589.8	3	14	6.3	26.7	3.41	5.44	62	15
GCAB_MOUSE	Ig gamma-2A chain C region secreted form OS=Mus musculus PE=1 SV=1	53.9	181.4	2	3	7.5	12.2	7.29	3.38	89	56
IGG2B_MOUSE	Ig gamma-2B chain C region OS=Mus musculus GN=Igh-3 PE=1 SV=3	151.1	173.5	3	5	8.7	15.8	9.17	4.8	50	60
IGHG3_MOUSE	Ig gamma-3 chain C region OS=Mus musculus PE=1 SV=2	366.1	322	6	5	18.3	16.3	3.53	4.42	21	33
HVM06_MOUSE	Ig heavy chain V region 102 OS=Mus musculus PE=1 SV=1	86.5	140.6	2	2	23.1	18.8	6.22	7.51	74	69
HVM14_MOUSE	Ig heavy chain V region 108A OS=Mus musculus GN=Igh-VJ558 PE=4 SV=1	43.7	73.4	1	1	12.8	12.8	1.93	3.24	95	96
HVM09_MOUSE	Ig heavy chain V region 186-1 OS=Mus musculus PE=4 SV=2	92	107.5	2	2	18.8	18.8	6.64	7.45	71	81
HVM05_MOUSE	Ig heavy chain V region 3 OS=Mus musculus GN=Ighv1-61 PE=1 SV=1	58.7	123.9	1	2	20.5	26.5	5.1	6.26	87	76
HVM60_MOUSE	Ig heavy chain V region 3-6 OS=Mus musculus GN=Ighv3-6 PE=1 SV=1	112.1	106.9	1	1	13.8	13.8	2.41	0.17	60	82
HVM03_MOUSE	Ig heavy chain V region 36-65 OS=Mus musculus PE=1 SV=1	128.8	160.7	3	3	20	20	2.17	2.87	56	64
HVM36_MOUSE	Ig heavy chain V region 441 OS=Mus musculus PE=4 SV=1	66.5	95.6	2	3	11.2	19.8	6.93	5.28	84	85
HVM57_MOUSE	Ig heavy chain V region 6.96 OS=Mus musculus PE=4 SV=1	98.7	168	2	3	24.5	24.5	2.76	4.23	68	62
HVM51_MOUSE	Ig heavy chain V region AC38 205.12 OS=Mus musculus PE=1 SV=1	387.1	474.8	4	5	57.6	63.6	5.63	4.82	19	21
HVM16_MOUSE	Ig heavy chain V region MOPC 21 (Fragment) OS=Mus musculus PE=1 SV=1	86.2	81.9	2	2	11.8	11.8	3.37	4.85	75	93
HVM17_MOUSE	Ig heavy chain V region MOPC 47A OS=Mus musculus PE=1 SV=1	71.9	55.9	2	2	12	12	2.2	3.42	82	104
HVM52_MOUSE	Ig heavy chain V region VH558 A1/A4 OS=Mus musculus GN=Gm5629 PE=2 SV=1	41.6	44.4	1	1	20.5	20.5	6.99	4.16	98	109
HVM35_MOUSE	Ig heavy chain V-III region HPC76 (Fragment) OS=Mus musculus PE=4 SV=1	70.9	69.3	2	2	12.6	12.6	2.09	2.99	83	99
IGKC_MOUSE	Ig kappa chain C region OS=Mus musculus PE=1 SV=1	1247.6	1183.7	12	13	70.8	78.3	4.12	4.44	4	7
KV2A7_MOUSE	Ig kappa chain V-II region 26-10 OS=Mus musculus PE=1 SV=1	216.2	203.9	4	3	43.4	25.7	3.37	4.23	33	53
KV2A6_MOUSE	Ig kappa chain V-II region 7S34.1 OS=Mus musculus PE=1 SV=1	129.2	114.6	2	2	21.2	21.2	6.82	5.43	55	78
KV3AC_MOUSE	Ig kappa chain V-III region CBPC 101 OS=Mus musculus PE=1 SV=1	193.4	253.2	3	3	68.5	68.5	3.92	4.13	40	46
KV3A3_MOUSE	Ig kappa chain V-III region MOPC 70 OS=Mus musculus PE=1 SV=1	187	0	2	0	30.6	0	3.16	0	43	0
KV3AM_MOUSE	Ig kappa chain V-III region PC 2154 OS=Mus musculus PE=1 SV=1	92.5	90.9	1	1	11.1	11.1	3.68	5.29	70	87
KV3A8_MOUSE	Ig kappa chain V-III region PC 3741/TEPC 111 OS=Mus musculus PE=1 SV=1	208.8	258	4	4	53.2	53.2	4.07	4.43	36	42
KV3AI_MOUSE	Ig kappa chain V-III region PC 6684 OS=Mus musculus PE=1 SV=1	196.6	253.3	3	4	40.5	68.5	4	5.24	39	45
KV4A1_MOUSE	Ig kappa chain V-IV region S107B OS=Mus musculus PE=4 SV=1	74.7	62.4	1	1	8.5	8.5	8.41	7.47	80	100
KV5AB_MOUSE	Ig kappa chain V-V region HP R16.7 OS=Mus musculus PE=1 SV=1	215.4	0	4	0	53.7	0	4.17	0	34	0
KV5A3_MOUSE	Ig kappa chain V-V region K2 (Fragment) OS=Mus musculus PE=1 SV=1	248.9	220.6	4	4	40.9	40.9	4.53	6.62	30	52
KV5A6_MOUSE	Ig kappa chain V-V region L6 (Fragment) OS=Mus musculus PE=4 SV=1	191.1	188.5	3	3	35.7	35.7	0.87	3.26	41	55
KV5A9_MOUSE	Ig kappa chain V-V region L7 (Fragment) OS=Mus musculus GN=Gm10881 PE=1 SV=1	40.4	72.8	1	1	13	13	0.53	2.11	100	97
KV5A4_MOUSE	Ig kappa chain V-V region MOPC 149 OS=Mus musculus PE=1 SV=1	234.2	262.6	2	2	25.9	25.9	6.53	7.21	32	41
KV5A7_MOUSE	Ig kappa chain V-V region MOPC 41 OS=Mus musculus GN=Gm5571 PE=1 SV=1	99.5	201.2	2	3	27.7	28.5	3.34	5.17	67	54
KV6AB_MOUSE	Ig kappa chain V-VI region NQ2-6.1 OS=Mus musculus PE=2 SV=1	116.7	137.8	1	1	14.8	14.8	2.41	9.31	58	70
KV5A1_MOUSE	Ig kappa chain V19-17 OS=Mus musculus GN=Igk-V19-17 PE=1 SV=1	104.9	173.3	2	4	12.1	16.8	2.81	4.03	64	61
LV1B_MOUSE	Ig lambda-1 chain V regions MOPC 104E/RPC20/J558/S104 OS=Mus musculus PE=1 SV=1	86.9	0	2	0	32.6	0	3.07	0	73	0

IGHM_MOUSE	Ig mu chain C region OS=Mus musculus GN=Ighm PE=1 SV=2	2029.9	2154.1	30	31	63.9	66.7	4.5	3.66	1	3
IGJ_MOUSE	Immunoglobulin J chain OS=Mus musculus GN=Jchain PE=1 SV=4	486.1	543.8	9	11	59.1	59.1	1.73	4.04	13	19
ITIH4_MOUSE	Inter alpha-trypsin inhibitor, heavy chain 4 OS=Mus musculus GN=Itih4 PE=1 SV=2	246.5	706.9	6	14	10	21.1	3.68	5.77	31	11
KRA65_MOUSE	Keratin-associated protein 6-5 OS=Mus musculus GN=Krtap6-5 PE=2 SV=1	45.6	0	1	0	19.2	0	0.62	0	94	0
KT33A_MOUSE	Keratin, type I cuticular Ha3-I OS=Mus musculus GN=Krt33a PE=1 SV=1	168.5	0	5	0	20.8	0	4.73	0	45	0
K1C10_MOUSE	Keratin, type I cytoskeletal 10 OS=Mus musculus GN=Krt10 PE=1 SV=3	135.6	0	3	0	6	0	5.56	0	54	0
K1C15_MOUSE	Keratin, type I cytoskeletal 15 OS=Mus musculus GN=Krt15 PE=1 SV=2	105.8	0	2	0	3.5	0	5.86	0	63	0
KRT86_MOUSE	Keratin, type II cuticular Hb6 OS=Mus musculus GN=Krt86 PE=2 SV=2	375.2	0	8	0	19.5	0	1.8	0	20	0
K22E_MOUSE	Keratin, type II cytoskeletal 2 epidermal OS=Mus musculus GN=Krt2 PE=1 SV=1	43	53.6	1	1	2	2	1.9	8.35	96	105
K2C6A_MOUSE	Keratin, type II cytoskeletal 6A OS=Mus musculus GN=Krt6a PE=1 SV=3	50.3	0	1	0	2.2	0	7.32	0	91	0
K2C74_MOUSE	Keratin, type II cytoskeletal 74 OS=Mus musculus GN=Krt74 PE=3 SV=1	63.4	0	1	0	2.4	0	2.67	0	85	0
LBP_MOUSE	Lipopolysaccharide-binding protein OS=Mus musculus GN=Lbp PE=1 SV=2	40.4	0	1	0	2.5	0	6.11	0	99	0
LOXL1_MOUSE	Lysyl oxidase homolog 1 OS=Mus musculus GN=Loxl1 PE=2 SV=3	63.4	270.4	1	6	3.1	18.6	0.38	3.49	86	40
MASP1_MOUSE	Mannan-binding lectin serine protease 1 OS=Mus musculus GN=Masp1 PE=1 SV=2	78.1	231.9	3	5	5	8.4	5.56	3.25	78	51
MASP2_MOUSE	Mannan-binding lectin serine protease 2 OS=Mus musculus GN=Masp2 PE=1 SV=1	214	381.2	5	7	11.2	14.5	5.81	5.33	35	28
MBL1_MOUSE	Mannose-binding protein A OS=Mus musculus GN=Mbl1 PE=1 SV=1	143.8	291	2	5	9.6	28.9	4.63	3.29	53	37
MBL2_MOUSE	Mannose-binding protein C OS=Mus musculus GN=Mbl2 PE=1 SV=2	474.4	584.7	8	10	38.9	48	3.71	4.38	15	16
IC1_MOUSE	Plasma protease C1 inhibitor OS=Mus musculus GN=Serping1 PE=1 SV=3	85.8	147.8	1	3	3.4	10.3	0.22	4.05	76	67
PLF4_MOUSE	Platelet factor 4 OS=Mus musculus GN=Pf4 PE=1 SV=1	48.1	0	1	0	11.4	0	7.54	0	93	0
PZP_MOUSE	Pregnancy zone protein OS=Mus musculus GN=Pzp PE=1 SV=3	187.9	408.7	5	9	3.7	10.1	5.97	4.53	42	25
PROP_MOUSE	Properdin OS=Mus musculus GN=Cfp PE=2 SV=2	167	250.8	3	6	9.9	15.7	4.29	4.12	46	47
THRB_MOUSE	Prothrombin OS=Mus musculus GN=F2 PE=1 SV=1	165	303.8	3	6	6.6	13.8	4.76	3.38	48	35
SPA3K_MOUSE	Serine protease inhibitor A3K OS=Mus musculus GN=Serpina3k PE=1 SV=2	90.6	234.6	3	7	12.4	28.7	7.2	4.32	72	50
TRFE_MOUSE	Serotransferrin OS=Mus musculus GN=Tf PE=1 SV=1	647.3	496.9	12	9	21.2	15.6	4.35	5.4	7	20
ALBU_MOUSE	Serum albumin OS=Mus musculus GN=Alb PE=1 SV=3	1912.4	2371.7	32	37	63.2	74.2	3.66	4.3	2	2
TETN_MOUSE	Tetranectin OS=Mus musculus GN=Clec3b PE=1 SV=2	48.9	71.3	2	1	22.3	16.3	3.82	2.52	92	98
TSP1_MOUSE	Thrombospondin-1 OS=Mus musculus GN=Thbs1 PE=1 SV=1	460.5	666.7	11	17	10.8	16.3	4.31	4.67	16	12
ZEP2_MOUSE	Transcription factor HIVEP2 OS=Mus musculus GN=Hivep2 PE=1 SV=1	42.7	0	1	0	0.5	0	9.26	0	97	0
TTHY_MOUSE	Transthyretin OS=Mus musculus GN=Ttr PE=1 SV=1	171.8	373.7	5	7	55.8	62.6	4.66	3.31	44	29
VTDB_MOUSE	Vitamin D-binding protein OS=Mus musculus GN=Gc PE=1 SV=2	113.8	175.7	4	5	12.4	17	1.62	4.37	59	59

6h Ball Milled SWCNTs

Accession	Name	Meta Score A	Meta Score B	Peptides A	Peptides B	SC [%] A	SC [%] B	RMS90 [ppm] A	RMS90 [ppm] B	Rank A	Rank B
ACTB_MOUSE	Actin, cytoplasmic 1 OS=Mus musculus GN=Actb PE=1 SV=1	125.7	0	3	0	10.7	0	4.63	0	64	0
A1AT2_MOUSE	Alpha-1-antitrypsin 1-2 OS=Mus musculus GN=Serpina1b PE=1 SV=2	153.7	0	4	0	11.1	0	5.64	0	57	0
A1AT3_MOUSE	Alpha-1-antitrypsin 1-3 OS=Mus musculus GN=Serpina1c PE=1 SV=2	189	176.8	4	6	12.9	20.4	4.2	3.3	46	58
A1AT4_MOUSE	Alpha-1-antitrypsin 1-4 OS=Mus musculus GN=Serpina1d PE=1 SV=1	170.2	155.3	4	5	13.3	18.2	5.68	4.78	52	65
FETUA_MOUSE	Alpha-2-HS-glycoprotein OS=Mus musculus GN=Ahsg PE=1 SV=1	500.9	988.8	5	12	29.3	35.9	2.69	2.97	16	9
APOA1_MOUSE	Apolipoprotein A-I OS=Mus musculus GN=Apoa1 PE=1 SV=2	546.2	1201	11	21	53.8	69.3	4.38	4.19	13	6
APOA2_MOUSE	Apolipoprotein A-II OS=Mus musculus GN=Apoa2 PE=1 SV=2	63.4	124.5	1	2	9.8	32.4	1.84	2.64	92	75
APOA4_MOUSE	Apolipoprotein A-IV OS=Mus musculus GN=Apoa4 PE=1 SV=3	400.2	935.3	10	15	33.2	50.6	3.26	3.91	26	10
APOB_MOUSE	Apolipoprotein B-100 OS=Mus musculus GN=Apob PE=1 SV=1	534.4	0	14	0	4.1	0	4.46	0	14	0
APOC1_MOUSE	Apolipoprotein C-I OS=Mus musculus GN=Apoc1 PE=1 SV=1	156.6	153.9	4	3	25	22.7	3.59	6.3	56	66
APOC2_MOUSE	Apolipoprotein C-II OS=Mus musculus GN=Apoc2 PE=2 SV=1	184.7	283.1	3	4	30.9	33	4.03	1.59	47	38
APOC3_MOUSE	Apolipoprotein C-III OS=Mus musculus GN=Apoc3 PE=1 SV=2	318.4	398.1	4	5	50.5	50.5	1.92	4.58	29	27
APOE_MOUSE	Apolipoprotein E OS=Mus musculus GN=Apoe PE=1 SV=2	823.7	1087.7	17	21	53.1	50.2	3.5	3.59	7	8
APOH_MOUSE	Beta-2-glycoprotein 1 OS=Mus musculus GN=Apoh PE=1 SV=1	48.5	330.3	1	9	5.8	29	5.06	4.17	103	31
B2MG_MOUSE	Beta-2-microglobulin OS=Mus musculus GN=B2m PE=1 SV=2	62.7	58.5	1	1	7.6	7.6	0.26	4.06	93	101
CRP_MOUSE	C-reactive protein OS=Mus musculus GN=Crp PE=1 SV=2	460.4	239.7	9	6	42.2	38.2	4.85	2.1	21	49
C4BPA_MOUSE	C4b-binding protein OS=Mus musculus GN=C4bpa PE=1 SV=3	220.6	405.3	5	7	15.4	19.8	5.11	3.16	38	26
CD5L_MOUSE	CD5 antigen-like OS=Mus musculus GN=Cd5l PE=1 SV=3	931.8	1354.7	12	23	37.2	60.5	3.86	3.82	6	5

CLUS_MOUSE	Clusterin OS=Mus musculus GN=Clu PE=1 SV=1	237.1	653.4	6	13	21.9	31.7	2.2	4.81	35	13
FA5_MOUSE	Coagulation factor V OS=Mus musculus GN=F5 PE=1 SV=1	95.5	178	2	4	1.2	2.7	1.29	6.11	77	57
C1QA_MOUSE	Complement C1q subcomponent subunit A OS=Mus musculus GN=C1qa PE=1 SV=2	193.9	129	5	2	24.5	14.3	3.41	3.49	42	73
C1QB_MOUSE	Complement C1q subcomponent subunit B OS=Mus musculus GN=C1qb PE=1 SV=2	427.7	433.2	6	7	27.3	36	2.45	3.98	24	24
C1QC_MOUSE	Complement C1q subcomponent subunit C OS=Mus musculus GN=C1qc PE=1 SV=2	175.5	94.3	3	1	16.7	7.3	4.99	5.42	50	86
C1RA_MOUSE	Complement C1r-A subcomponent OS=Mus musculus GN=C1ra PE=1 SV=1	586.5	621	12	13	31.1	34.8	2.09	4.05	12	14
CS1A_MOUSE	Complement C1s-A subcomponent OS=Mus musculus GN=C1sa PE=2 SV=2	486.9	298.8	9	5	20.2	12.1	5.02	5.3	18	36
CO3_MOUSE	Complement C3 OS=Mus musculus GN=C3 PE=1 SV=3	2208	3021.5	46	60	36.1	43.4	3.73	4.69	1	1
CO4B_MOUSE	Complement C4-B OS=Mus musculus GN=C4b PE=1 SV=3	429.7	464.2	10	11	11	10.2	3.17	4.13	23	22
CO5_MOUSE	Complement C5 OS=Mus musculus GN=C5 PE=1 SV=2	41.4	0	1	0	1	0	5.38	0	105	0
CFAH_MOUSE	Complement factor H OS=Mus musculus GN=Cfh PE=1 SV=2	469.3	1599.5	12	32	14.8	39.7	3.93	5.38	19	4
FIBA_MOUSE	Fibrinogen alpha chain OS=Mus musculus GN=Fga PE=1 SV=1	358.4	240.4	8	4	14.6	8.4	3.49	6.87	27	48
FIBB_MOUSE	Fibrinogen beta chain OS=Mus musculus GN=Fgb PE=1 SV=1	586.8	0	14	0	49.1	0	4.48	0	11	0
FIBG_MOUSE	Fibrinogen gamma chain OS=Mus musculus GN=Fgg PE=1 SV=1	681.7	135.4	12	4	44	15.4	4.19	3.13	8	72
FINC_MOUSE	Fibronectin OS=Mus musculus GN=Fn1 PE=1 SV=4	1261.5	346.1	29	8	20.5	5.4	3.76	4.41	5	30
GELS_MOUSE	Gelsolin OS=Mus musculus GN=Gsn PE=1 SV=3	461.9	564.3	10	12	25.3	23.1	4.2	4.43	20	18
GPX3_MOUSE	Glutathione peroxidase 3 OS=Mus musculus GN=Gpx3 PE=1 SV=2	83.1	0	3	0	13.3	0	5.89	0	83	0
HA10_MOUSE	H-2 class I histocompatibility antigen, Q10 alpha chain OS=Mus musculus GN=H2-Q10 PE=1 SV=3	135.1	328	3	6	11.4	22.2	3.78	3.08	61	32
HBA_MOUSE	Hemoglobin subunit alpha OS=Mus musculus GN=Hba PE=1 SV=2	179.9	114.3	4	2	33.1	16.9	5.28	2.57	49	79
HBB1_MOUSE	Hemoglobin subunit beta-1 OS=Mus musculus GN=Hbb-b1 PE=1 SV=2	59.6	0	1	0	6.8	0	4.33	0	95	0
HEMO_MOUSE	Hemopexin OS=Mus musculus GN=Hpx PE=1 SV=2	325.9	438.2	8	10	21.5	25.4	4.48	5.58	28	23
HRG_MOUSE	Histidine-rich glycoprotein OS=Mus musculus GN=Hrg PE=1 SV=2	94.5	589.8	3	14	6.3	26.7	5.17	5.44	79	15
GCAB_MOUSE	Ig gamma-2A chain C region secreted form OS=Mus musculus PE=1 SV=1	94.6	181.4	3	3	12.5	12.2	5.48	3.38	78	56
IGG2B_MOUSE	Ig gamma-2B chain C region OS=Mus musculus GN=Igh-3 PE=1 SV=3	142.7	173.5	4	5	13.6	15.8	7.01	4.8	60	60
IGHG3_MOUSE	Ig gamma-3 chain C region OS=Mus musculus PE=1 SV=2	487.8	322	8	5	28.6	16.3	4.37	4.42	17	33
HVM06_MOUSE	Ig heavy chain V region 102 OS=Mus musculus PE=1 SV=1	102.4	140.6	2	2	23.1	18.8	5.44	7.51	73	69
HVM14_MOUSE	Ig heavy chain V region 108A OS=Mus musculus GN=Igh-VJ558 PE=4 SV=1	49.5	73.4	1	1	12.8	12.8	0.6	3.24	102	96
HVM09_MOUSE	Ig heavy chain V region 186-1 OS=Mus musculus PE=4 SV=2	96.3	107.5	2	2	18.8	18.8	1.74	7.45	76	81
HVM05_MOUSE	Ig heavy chain V region 3 OS=Mus musculus GN=Ighv1-61 PE=1 SV=1	60.7	123.9	1	2	20.5	26.5	7.15	6.26	94	76
HVM60_MOUSE	Ig heavy chain V region 3-6 OS=Mus musculus GN=Ighv3-6 PE=1 SV=1	116.1	106.9	1	1	13.8	13.8	2.2	0.17	69	82
HVM03_MOUSE	Ig heavy chain V region 36-65 OS=Mus musculus PE=1 SV=1	117	160.7	2	3	20	20	3.49	2.87	68	64
HVM36_MOUSE	Ig heavy chain V region 441 OS=Mus musculus PE=4 SV=1	69.9	95.6	2	3	17.2	19.8	7.5	5.28	89	85
HVM57_MOUSE	Ig heavy chain V region 6.96 OS=Mus musculus PE=4 SV=1	79.4	168	1	3	19.4	24.5	3.53	4.23	85	62
HVM51_MOUSE	Ig heavy chain V region AC38 205.12 OS=Mus musculus PE=1 SV=1	419.9	474.8	4	5	57.6	63.6	2.89	4.82	25	21
HVM16_MOUSE	Ig heavy chain V region MOPC 21 (Fragment) OS=Mus musculus PE=1 SV=1	63.4	81.9	1	2	8.1	11.8	2.36	4.85	91	93
HVM52_MOUSE	Ig heavy chain V region VH558 A1/A4 OS=Mus musculus GN=Gm5629 PE=2 SV=1	71.7	44.4	1	1	20.5	20.5	0.52	4.16	87	109
HVM35_MOUSE	Ig heavy chain V-III region HPC76 (Fragment) OS=Mus musculus PE=4 SV=1	53	69.3	1	2	8.1	12.6	8.78	2.99	99	99
IGKC_MOUSE	Ig kappa chain C region OS=Mus musculus PE=1 SV=1	1265.6	1183.7	13	13	75.5	78.3	2.78	4.44	4	7
KV2A7_MOUSE	Ig kappa chain V-II region 26-10 OS=Mus musculus PE=1 SV=1	189.8	203.9	4	3	46.9	25.7	2.96	4.23	45	53
KV2A6_MOUSE	Ig kappa chain V-II region 7S34.1 OS=Mus musculus PE=1 SV=1	110	114.6	2	2	21.2	21.2	4.79	5.43	72	78
KV3A4_MOUSE	Ig kappa chain V-III region 50S10.1 OS=Mus musculus PE=1 SV=1	257	256.6	3	3	40.5	40.5	3.84	5.06	32	43
KV3AC_MOUSE	Ig kappa chain V-III region CBPC 101 OS=Mus musculus PE=1 SV=1	218.6	253.2	2	3	30.6	68.5	5.47	4.13	39	46
KV3AM_MOUSE	Ig kappa chain V-III region PC 2154 OS=Mus musculus PE=1 SV=1	113.6	90.9	1	1	11.1	11.1	4.04	5.29	70	87
KV3A7_MOUSE	Ig kappa chain V-III region TEPC 124 OS=Mus musculus PE=1 SV=1	233.7	0	4	0	30.4	0	5.4	0	37	0
KV4A1_MOUSE	Ig kappa chain V-IV region S107B OS=Mus musculus PE=4 SV=1	43.3	62.4	1	1	8.5	8.5	4.61	7.47	104	100
KV5AC_MOUSE	Ig kappa chain V-V region HP 93G7 OS=Mus musculus PE=1 SV=1	240.9	253.9	4	5	53.7	59.3	4.76	5.27	34	44
KV5A3_MOUSE	Ig kappa chain V-V region K2 (Fragment) OS=Mus musculus PE=1 SV=1	246.8	220.6	4	4	40.9	40.9	4.08	6.62	33	52
KV5A6_MOUSE	Ig kappa chain V-V region L6 (Fragment) OS=Mus musculus PE=4 SV=1	133.6	188.5	3	3	35.7	35.7	4.42	3.26	62	55
KV5A9_MOUSE	Ig kappa chain V-V region L7 (Fragment) OS=Mus musculus GN=Gm10881 PE=1 SV=1	56.1	72.8	1	1	13	13	1.7	2.11	97	97
KV5A4_MOUSE	Ig kappa chain V-V region MOPC 149 OS=Mus musculus PE=1 SV=1	214.3	262.6	2	2	25.9	25.9	4.35	7.21	40	41
KV5A7_MOUSE	Ig kappa chain V-V region MOPC 41 OS=Mus musculus GN=Gm5571 PE=1 SV=1	193.1	201.2	3	3	28.5	28.5	4.03	5.17	43	54
KV6A7_MOUSE	Ig kappa chain V-VI region NQ2-48.2.2 OS=Mus musculus PE=2 SV=1	50.5	113.6	2	3	25.2	32.7	1.82	5.8	101	80

KV6AB_MOUSE	Ig kappa chain V-VI region NQ2-6.1 OS=Mus musculus PE=2 SV=1	122.8	137.8	1	1	14.8	14.8	7.58	9.31	66	70
KV5A1_MOUSE	Ig kappa chain V19-17 OS=Mus musculus GN=Igk-V19-17 PE=1 SV=1	99.7	173.3	2	4	12.1	16.8	4.66	4.03	74	61
LAC1_MOUSE	Ig lambda-1 chain C region OS=Mus musculus PE=1 SV=1	90.2	97.4	2	2	35.2	35.2	2.71	4.89	80	84
LV1B_MOUSE	Ig lambda-1 chain V regions MOPC 104E/RPC20/J558/S104 OS=Mus musculus PE=1 SV=1	79.8	0	1	0	7	0	4.66	0	84	0
LAC2_MOUSE	Ig lambda-2 chain C region OS=Mus musculus GN=Iglc2 PE=1 SV=1	56.7	81.1	2	2	33.7	33.7	1.64	6.46	96	94
IGHM_MOUSE	Ig mu chain C region OS=Mus musculus GN=Ighm PE=1 SV=2	2065.9	2154.1	27	31	61.9	66.7	3.45	3.66	2	3
IGJ_MOUSE	Immunoglobulin J chain OS=Mus musculus GN=Jchain PE=1 SV=4	436.8	543.8	8	11	59.1	59.1	3.32	4.04	22	19
ITIH4_MOUSE	Inter alpha-trypsin inhibitor, heavy chain 4 OS=Mus musculus GN=Itih4 PE=1 SV=2	303.6	706.9	8	14	12.2	21.1	4.99	5.77	31	11
KRT35_MOUSE	Keratin, type I cuticular Ha5 OS=Mus musculus GN=Krt35 PE=1 SV=1	70.2	0	2	0	4.2	0	3.5	0	88	0
K1C10_MOUSE	Keratin, type I cytoskeletal 10 OS=Mus musculus GN=Krt10 PE=1 SV=3	306.9	0	6	0	10.9	0	3.86	0	30	0
K1C13_MOUSE	Keratin, type I cytoskeletal 13 OS=Mus musculus GN=Krt13 PE=1 SV=2	167.3	0	4	0	8.9	0	4.43	0	53	0
K1C14_MOUSE	Keratin, type I cytoskeletal 14 OS=Mus musculus GN=Krt14 PE=1 SV=2	160.8	0	4	0	8.3	0	2.84	0	55	0
K2C1_MOUSE	Keratin, type II cytoskeletal 1 OS=Mus musculus GN=Krt1 PE=1 SV=4	175.5	0	4	0	4.9	0	6.3	0	51	0
K2C1B_MOUSE	Keratin, type II cytoskeletal 1b OS=Mus musculus GN=Krt77 PE=1 SV=1	150.1	0	3	0	5.4	0	5.8	0	58	0
K2C6A_MOUSE	Keratin, type II cytoskeletal 6A OS=Mus musculus GN=Krt6a PE=1 SV=3	149.7	0	3	0	5.4	0	5.12	0	59	0
K2C79_MOUSE	Keratin, type II cytoskeletal 79 OS=Mus musculus GN=Krt79 PE=1 SV=2	96.5	0	2	0	3.6	0	5.27	0	75	0
LBP_MOUSE	Lipopolysaccharide-binding protein OS=Mus musculus GN=Lbp PE=1 SV=2	75.4	0	3	0	9.4	0	2.13	0	86	0
LOXL1_MOUSE	Lysyl oxidase homolog 1 OS=Mus musculus GN=Loxl1 PE=2 SV=3	51.6	270.4	1	6	3.1	18.6	3.47	3.49	100	40
MASP1_MOUSE	Mannan-binding lectin serine protease 1 OS=Mus musculus GN=Masp1 PE=1 SV=2	162	231.9	6	5	9.5	8.4	2.56	3.25	54	51
MASP2_MOUSE	Mannan-binding lectin serine protease 2 OS=Mus musculus GN=Masp2 PE=1 SV=1	236.7	381.2	5	7	8.3	14.5	4.53	5.33	36	28
MBL1_MOUSE	Mannose-binding protein A OS=Mus musculus GN=Mbl1 PE=1 SV=1	120.3	291	2	5	9.6	28.9	3.05	3.29	67	37
MBL2_MOUSE	Mannose-binding protein C OS=Mus musculus GN=Mbl2 PE=1 SV=2	588.8	584.7	9	10	40.2	48	5.99	4.38	10	16
MUG1_MOUSE	Murinoglobulin-1 OS=Mus musculus GN=Mug1 PE=1 SV=3	55.3	88	2	2	1.9	1.7	6.39	8.59	98	89
PLTP_MOUSE	Phospholipid transfer protein OS=Mus musculus GN=Pltp PE=1 SV=1	66.6	0	2	0	6.1	0	3.79	0	90	0
IC1_MOUSE	Plasma protease C1 inhibitor OS=Mus musculus GN=Serping1 PE=1 SV=3	112.8	147.8	2	3	5.6	10.3	6.42	4.05	71	67
PZP_MOUSE	Pregnancy zone protein OS=Mus musculus GN=Pzp PE=1 SV=3	183.7	408.7	6	9	6.4	10.1	3.26	4.53	48	25
PROP_MOUSE	Properdin OS=Mus musculus GN=Cfp PE=2 SV=2	191.4	250.8	3	6	9.9	15.7	3.22	4.12	44	47
THRB_MOUSE	Prothrombin OS=Mus musculus GN=F2 PE=1 SV=1	126.8	303.8	4	6	9.2	13.8	4.85	3.38	63	35
SPA3K_MOUSE	Serine protease inhibitor A3K OS=Mus musculus GN=Serpina3k PE=1 SV=2	122.9	234.6	4	7	17	28.7	4.94	4.32	65	50
TRFE_MOUSE	Serotransferrin OS=Mus musculus GN=Tf PE=1 SV=1	592.8	496.9	13	9	24.7	15.6	5.01	5.4	9	20
ALBU_MOUSE	Serum albumin OS=Mus musculus GN=Alb PE=1 SV=3	1894.2	2371.7	29	37	63.2	74.2	3.04	4.3	3	2
QSOX1_MOUSE	Sulfhydryl oxidase 1 OS=Mus musculus GN=Qsox1 PE=1 SV=1	87.3	0	3	0	4.5	0	3.86	0	81	0
TSP1_MOUSE	Thrombospondin-1 OS=Mus musculus GN=Thbs1 PE=1 SV=1	523.2	666.7	14	17	13.6	16.3	3.44	4.67	15	12
TTHY_MOUSE	Transthyretin OS=Mus musculus GN=Ttr PE=1 SV=1	199.5	373.7	3	7	46.9	62.6	4.41	3.31	41	29
VTDB_MOUSE	Vitamin D-binding protein OS=Mus musculus GN=Gc PE=1 SV=2	86.7	175.7	3	5	10.7	17	4.89	4.37	82	59

8h Ball Milled SWCNTs

Accession	Name	Meta Score A	Meta Score B	Peptides A	Peptides B	SC [%] A	SC [%] B	RMS90 [ppm] A	RMS90 [ppm] B	Rank A	Rank B
ACTB_MOUSE	Actin, cytoplasmic 1 OS=Mus musculus GN=Actb PE=1 SV=1	60.6	0	1	0	4.3	0	4.39	0	75	0
A1AT2_MOUSE	Alpha-1-antitrypsin 1-2 OS=Mus musculus GN=Serpina1b PE=1 SV=2	53.4	0	2	0	7.3	0	4.34	0	80	0
A1AT3_MOUSE	Alpha-1-antitrypsin 1-3 OS=Mus musculus GN=Serpina1c PE=1 SV=2	96	176.8	3	6	11.9	20.4	4.44	3.3	59	58
A1AT4_MOUSE	Alpha-1-antitrypsin 1-4 OS=Mus musculus GN=Serpina1d PE=1 SV=1	60.2	155.3	2	5	6.5	18.2	3.88	4.78	76	65
A1AT5_MOUSE	Alpha-1-antitrypsin 1-5 OS=Mus musculus GN=Serpina1e PE=1 SV=1	57.8	0	2	0	7.3	0	4.4	0	77	0
FETUA_MOUSE	Alpha-2-HS-glycoprotein OS=Mus musculus GN=Ahsg PE=1 SV=1	355.1	988.8	7	12	29.3	35.9	4.24	2.97	17	9
APOA1_MOUSE	Apolipoprotein A-I OS=Mus musculus GN=Apoa1 PE=1 SV=2	656.8	1201	12	21	38.3	69.3	4.69	4.19	7	6
APOA2_MOUSE	Apolipoprotein A-II OS=Mus musculus GN=Apoa2 PE=1 SV=2	92.4	124.5	2	2	14.7	32.4	3.81	2.64	62	75
APOA4_MOUSE	Apolipoprotein A-IV OS=Mus musculus GN=Apoa4 PE=1 SV=3	436.8	935.3	9	15	29.9	50.6	4.42	3.91	12	10
APOB_MOUSE	Apolipoprotein B-100 OS=Mus musculus GN=Apob PE=1 SV=1	164.4	0	4	0	1	0	6.25	0	38	0
APOC1_MOUSE	Apolipoprotein C-I OS=Mus musculus GN=Apoc1 PE=1 SV=1	138.5	153.9	3	3	22.7	22.7	4.57	6.3	44	66
APOC2_MOUSE	Apolipoprotein C-II OS=Mus musculus GN=Apoc2 PE=2 SV=1	123.2	283.1	3	4	30.9	33	3.26	1.59	49	38
APOC3_MOUSE	Apolipoprotein C-III OS=Mus musculus GN=Apoc3 PE=1 SV=2	304.4	398.1	4	5	50.5	50.5	2.81	4.58	22	27

APOE_MOUSE	Apolipoprotein E OS=Mus musculus GN=Apoe PE=1 SV=2	707.1	1087.7	13	21	35.4	50.2	4.21	3.59	6	8
B2MG_MOUSE	Beta-2-microglobulin OS=Mus musculus GN=B2m PE=1 SV=2	71	58.5	1	1	7.6	7.6	6.81	4.06	68	101
CRP_MOUSE	C-reactive protein OS=Mus musculus GN=Crp PE=1 SV=2	418	239.7	9	6	42.2	38.2	4.3	2.1	14	49
CD5L_MOUSE	CD5 antigen-like OS=Mus musculus GN=Cd5l PE=1 SV=3	857.6	1354.7	13	23	39.2	60.5	3.15	3.82	5	5
CLUS_MOUSE	Clusterin OS=Mus musculus GN=Clu PE=1 SV=1	252.6	653.4	6	13	18.3	31.7	4.83	4.81	23	13
C1QA_MOUSE	Complement C1q subcomponent subunit A OS=Mus musculus GN=C1qa PE=1 SV=2	117	129	3	2	20	14.3	2.07	3.49	51	73
C1QB_MOUSE	Complement C1q subcomponent subunit B OS=Mus musculus GN=C1qb PE=1 SV=2	339.6	433.2	6	7	27.3	36	3.73	3.98	18	24
C1QC_MOUSE	Complement C1q subcomponent subunit C OS=Mus musculus GN=C1qc PE=1 SV=2	84.2	94.3	1	1	7.3	7.3	3.65	5.42	63	86
C1RA_MOUSE	Complement C1r-A subcomponent OS=Mus musculus GN=C1ra PE=1 SV=1	325.8	621	8	13	22.2	34.8	2.55	4.05	20	14
CS1A_MOUSE	Complement C1s-A subcomponent OS=Mus musculus GN=C1sa PE=2 SV=2	148.1	298.8	4	5	10.3	12.1	4.37	5.3	42	36
CO3_MOUSE	Complement C3 OS=Mus musculus GN=C3 PE=1 SV=3	1539.2	3021.5	32	60	25.6	43.4	4.64	4.69	3	1
CO4B_MOUSE	Complement C4-B OS=Mus musculus GN=C4b PE=1 SV=3	230.4	464.2	6	11	6.2	10.2	4.63	4.13	28	22
CFAH_MOUSE	Complement factor H OS=Mus musculus GN=Cfh PE=1 SV=2	130.5	1599.5	3	32	3.6	39.7	1.99	5.38	46	4
FIBA_MOUSE	Fibrinogen alpha chain OS=Mus musculus GN=Fga PE=1 SV=1	240.4	240.4	6	4	14.4	8.4	5.41	6.87	25	48
FIBB_MOUSE	Fibrinogen beta chain OS=Mus musculus GN=Fgb PE=1 SV=1	172.9	0	4	0	15.8	0	4.42	0	37	0
FIBG_MOUSE	Fibrinogen gamma chain OS=Mus musculus GN=Fgg PE=1 SV=1	466.6	135.4	9	4	28.4	15.4	4.17	3.13	11	72
FINC_MOUSE	Fibronectin OS=Mus musculus GN=Fn1 PE=1 SV=4	583.8	346.1	15	8	10.9	5.4	5.43	4.41	8	30
GELS_MOUSE	Gelsolin OS=Mus musculus GN=Gsn PE=1 SV=3	504.5	564.3	11	12	25.1	23.1	4.42	4.43	10	18
HA10_MOUSE	H-2 class I histocompatibility antigen, Q10 alpha chain OS=Mus musculus GN=H2-Q10 PE=1 SV=3	217.4	328	4	6	16	22.2	7.35	3.08	31	32
HBA_MOUSE	Hemoglobin subunit alpha OS=Mus musculus GN=Hba PE=1 SV=2	80.7	114.3	2	2	16.9	16.9	3.82	2.57	64	79
HEMO_MOUSE	Hemopexin OS=Mus musculus GN=Hpx PE=1 SV=2	417.4	438.2	9	10	23.9	25.4	3.66	5.58	15	23
HRG_MOUSE	Histidine-rich glycoprotein OS=Mus musculus GN=Hrg PE=1 SV=2	105.2	589.8	3	14	5	26.7	3.72	5.44	53	15
IGHG3_MOUSE	Ig gamma-3 chain C region OS=Mus musculus PE=1 SV=2	329.8	322	5	5	16.3	16.3	4.69	4.42	19	33
HVM06_MOUSE	Ig heavy chain V region 102 OS=Mus musculus PE=1 SV=1	56.7	140.6	2	2	23.1	18.8	4.37	7.51	78	69
HVM09_MOUSE	Ig heavy chain V region 186-1 OS=Mus musculus PE=4 SV=2	66.9	107.5	2	2	18.8	18.8	5.02	7.45	70	81
HVM60_MOUSE	Ig heavy chain V region 3-6 OS=Mus musculus GN=Ighv3-6 PE=1 SV=1	114.3	106.9	2	1	19	13.8	2.57	0.17	52	82
HVM57_MOUSE	Ig heavy chain V region 6.96 OS=Mus musculus PE=4 SV=1	65.8	168	1	3	19.4	24.5	0.57	4.23	71	62
HVM51_MOUSE	Ig heavy chain V region AC38 205.12 OS=Mus musculus PE=1 SV=1	234.2	474.8	4	5	44.9	63.6	4.11	4.82	27	21
HVM16_MOUSE	Ig heavy chain V region MOPC 21 (Fragment) OS=Mus musculus PE=1 SV=1	47.1	81.9	1	2	8.1	11.8	6.3	4.85	82	93
HVM52_MOUSE	Ig heavy chain V region VH558 A1/A4 OS=Mus musculus GN=Gm5629 PE=2 SV=1	43	44.4	1	1	20.5	20.5	5.8	4.16	86	109
HVM35_MOUSE	Ig heavy chain V-III region HPC76 (Fragment) OS=Mus musculus PE=4 SV=1	45	69.3	1	2	8.1	12.6	5.19	2.99	85	99
IGKC_MOUSE	Ig kappa chain C region OS=Mus musculus PE=1 SV=1	1007.4	1183.7	12	13	70.8	78.3	3.04	4.44	4	7
KV2A7_MOUSE	Ig kappa chain V-II region 26-10 OS=Mus musculus PE=1 SV=1	129.3	203.9	2	3	15.9	25.7	4.86	4.23	48	53
KV2A6_MOUSE	Ig kappa chain V-II region 7S34.1 OS=Mus musculus PE=1 SV=1	55.1	114.6	1	2	11.5	21.2	4.8	5.43	79	78
KV3A4_MOUSE	Ig kappa chain V-III region 50S10.1 OS=Mus musculus PE=1 SV=1	201.5	256.6	3	3	40.5	40.5	6.71	5.06	33	43
KV3AC_MOUSE	Ig kappa chain V-III region CBPC 101 OS=Mus musculus PE=1 SV=1	228.5	253.2	3	3	68.5	68.5	6.93	4.13	29	46
KV3AM_MOUSE	Ig kappa chain V-III region PC 2154 OS=Mus musculus PE=1 SV=1	104	90.9	1	1	11.1	11.1	7.33	5.29	54	87
KV3A8_MOUSE	Ig kappa chain V-III region PC 3741/TEPC 111 OS=Mus musculus PE=1 SV=1	182	258	3	4	30.6	53.2	7.79	4.43	36	42
KV3AB_MOUSE	Ig kappa chain V-III region PC 4050 OS=Mus musculus PE=1 SV=1	132.3	274.8	2	4	16.2	53.2	7.42	5.65	45	39
KV4A1_MOUSE	Ig kappa chain V-IV region S107B OS=Mus musculus PE=4 SV=1	61.5	62.4	1	1	8.5	8.5	6.99	7.47	73	100
KV5AB_MOUSE	Ig kappa chain V-V region HP R16.7 OS=Mus musculus PE=1 SV=1	218.1	0	4	0	53.7	0	3.93	0	30	0
KV5A3_MOUSE	Ig kappa chain V-V region K2 (Fragment) OS=Mus musculus PE=1 SV=1	239.9	220.6	4	4	40.9	40.9	5.18	6.62	26	52
KV5A6_MOUSE	Ig kappa chain V-V region L6 (Fragment) OS=Mus musculus PE=4 SV=1	79.3	188.5	2	3	22.6	35.7	4.38	3.26	65	55
KV5A9_MOUSE	Ig kappa chain V-V region L7 (Fragment) OS=Mus musculus GN=Gm10881 PE=1 SV=1	70.2	72.8	2	1	23.5	13	4.19	2.11	69	97
KV5A4_MOUSE	Ig kappa chain V-V region MOPC 149 OS=Mus musculus PE=1 SV=1	244	262.6	2	2	25.9	25.9	2.72	7.21	24	41
KV5A7_MOUSE	Ig kappa chain V-V region MOPC 41 OS=Mus musculus GN=Gm5571 PE=1 SV=1	149.6	201.2	3	3	28.5	28.5	4.94	5.17	40	54
KV6AB_MOUSE	Ig kappa chain V-VI region NQ2-6.1 OS=Mus musculus PE=2 SV=1	130.3	137.8	1	1	14.8	14.8	4.41	9.31	47	70
KV5A1_MOUSE	Ig kappa chain V19-17 OS=Mus musculus GN=Igk-V19-17 PE=1 SV=1	95.5	173.3	2	4	12.1	16.8	3.95	4.03	60	61
LV1C_MOUSE	Ig lambda-1 chain V region S178 OS=Mus musculus PE=1 SV=1	46.6	0	1	0	8.2	0	4.23	0	83	0
IGHM_MOUSE	Ig mu chain C region OS=Mus musculus GN=Ighm PE=1 SV=2	1839.3	2154.1	26	31	61.9	66.7	2.75	3.66	1	3
IGJ_MOUSE	Immunoglobulin J chain OS=Mus musculus GN=Jchain PE=1 SV=4	426.5	543.8	8	11	36.5	59.1	4.97	4.04	13	19
ITIH4_MOUSE	Inter alpha-trypsin inhibitor, heavy chain 4 OS=Mus musculus GN=Itih4 PE=1 SV=2	214.9	706.9	5	14	7.2	21.1	5.57	5.77	32	11

K1C10_MOUSE	Keratin, type I cytoskeletal 10 OS=Mus musculus GN=Krt10 PE=1 SV=3	75.9	0	2	0	3.9	0	3.48	0	66	0
K2C74_MOUSE	Keratin, type II cytoskeletal 74 OS=Mus musculus GN=Krt74 PE=3 SV=1	48.4	0	1	0	2.4	0	4.97	0	81	0
KNG1_MOUSE	Kininogen-1 OS=Mus musculus GN=Kng1 PE=1 SV=1	46	566.8	1	10	1.8	18.9	3.94	5.04	84	17
LBP_MOUSE	Lipopolysaccharide-binding protein OS=Mus musculus GN=Lbp PE=1 SV=2	62.2	0	1	0	2.5	0	6.19	0	72	0
LOXL1_MOUSE	Lysyl oxidase homolog 1 OS=Mus musculus GN=Loxl1 PE=2 SV=3	186.6	270.4	6	6	15.3	18.6	4.74	3.49	35	40
MASP1_MOUSE	Mannan-binding lectin serine protease 1 OS=Mus musculus GN=Masp1 PE=1 SV=2	99.9	231.9	3	5	5.3	8.4	6.17	3.25	58	51
MASP2_MOUSE	Mannan-binding lectin serine protease 2 OS=Mus musculus GN=Masp2 PE=1 SV=1	149	381.2	4	7	7	14.5	4.8	5.33	41	28
MBL1_MOUSE	Mannose-binding protein A OS=Mus musculus GN=Mbl1 PE=1 SV=1	119.1	291	3	5	12.6	28.9	4.73	3.29	50	37
MBL2_MOUSE	Mannose-binding protein C OS=Mus musculus GN=Mbl2 PE=1 SV=2	583.1	584.7	9	10	40.2	48	3.39	4.38	9	16
PRDX2_MOUSE	Peroxiredoxin-2 OS=Mus musculus GN=Prdx2 PE=1 SV=3	61.1	0	1	0	5.6	0	2.39	0	74	0
PLTP_MOUSE	Phospholipid transfer protein OS=Mus musculus GN=Pltp PE=1 SV=1	71	0	2	0	6.5	0	5.73	0	67	0
IC1_MOUSE	Plasma protease C1 inhibitor OS=Mus musculus GN=Serping1 PE=1 SV=3	94.9	147.8	2	3	8.1	10.3	3.03	4.05	61	67
PZP_MOUSE	Pregnancy zone protein OS=Mus musculus GN=Pzp PE=1 SV=3	101	408.7	3	9	2.1	10.1	3.81	4.53	57	25
PROP_MOUSE	Properdin OS=Mus musculus GN=Cfp PE=2 SV=2	104	250.8	3	6	9.9	15.7	2.17	4.12	55	47
THRB_MOUSE	Prothrombin OS=Mus musculus GN=F2 PE=1 SV=1	194.4	303.8	5	6	10	13.8	4.96	3.38	34	35
SPA3K_MOUSE	Serine protease inhibitor A3K OS=Mus musculus GN=Serpina3k PE=1 SV=2	147.1	234.6	2	7	10	28.7	6.08	4.32	43	50
TRFE_MOUSE	Serotransferrin OS=Mus musculus GN=Tf PE=1 SV=1	355.5	496.9	9	9	15.8	15.6	4.23	5.4	16	20
ALBU_MOUSE	Serum albumin OS=Mus musculus GN=Alb PE=1 SV=3	1709.3	2371.7	28	37	63.7	74.2	3.74	4.3	2	2
TSP1_MOUSE	Thrombospondin-1 OS=Mus musculus GN=Thbs1 PE=1 SV=1	324.4	666.7	9	17	9.2	16.3	4.09	4.67	21	12
TTHY_MOUSE	Transthyretin OS=Mus musculus GN=Ttr PE=1 SV=1	161.4	373.7	3	7	40.8	62.6	4.65	3.31	39	29
VTDB_MOUSE	Vitamin D-binding protein OS=Mus musculus GN=Gc PE=1 SV=2	102.1	175.7	3	5	10.7	17	1.19	4.37	56	59

Supplemental Data Table 4. Lipid Serum Venn Diagram Identification

Comparison of Proteins Associated with As Prepared and 2h Ball Milled SWCNTs
Accession Number

In Common	As Prepared Only	2h Ball Milled Only
A1AG1_MOUSE	CERU_MOUSE	A1AT2_MOUSE
A1AT3_MOUSE	CFAB_MOUSE	A2AP_MOUSE
A1AT4_MOUSE	F13B_MOUSE	ACTBL_MOUSE
ALBU_MOUSE	FA12_MOUSE	APOB_MOUSE
APOA1_MOUSE	FA5_MOUSE	FIBB_MOUSE
APOA2_MOUSE	FETUB_MOUSE	G3P_MOUSE
APOA4_MOUSE	HPT_MOUSE	HBB1_MOUSE
APOC1_MOUSE	HVM00_MOUSE	HVM10_MOUSE
APOC2_MOUSE	HVM09_MOUSE	HVM28_MOUSE
APOC3_MOUSE	HVM17_MOUSE	K1C10_MOUSE
APOE_MOUSE	HVM35_MOUSE	K2C1_MOUSE
APOH_MOUSE	IBP2_MOUSE	K2C6A_MOUSE
B2MG_MOUSE	IGHG1_MOUSE	KV3AE_MOUSE
C1QA_MOUSE	KV3AB_MOUSE	KV5AB_MOUSE
C1QB_MOUSE	KV5AC_MOUSE	LBP_MOUSE
C1QC_MOUSE	LAC2_MOUSE	LV1C_MOUSE
C1RA_MOUSE	LV1A_MOUSE	PLTP_MOUSE
C4BPA_MOUSE	MUG1_MOUSE	PRDX2_MOUSE
CD5L_MOUSE	MUP2_MOUSE	SPTA1_MOUSE
CFAH_MOUSE	RET4_MOUSE	
CLUS_MOUSE	SAA1_MOUSE	
CO3_MOUSE	SEPP1_MOUSE	
CO4B_MOUSE	SPA3M_MOUSE	
CRP_MOUSE	TETN_MOUSE	
CS1A_MOUSE		
FETUA_MOUSE		
FIBA_MOUSE		
FIBG_MOUSE		
FINC_MOUSE		
GCAB_MOUSE		
GELS_MOUSE		
HA10_MOUSE		
HBA_MOUSE		
HEMO_MOUSE		

HRG_MOUSE
HVM03_MOUSE
HVM05_MOUSE
HVM06_MOUSE
HVM14_MOUSE
HVM16_MOUSE
HVM36_MOUSE
HVM51_MOUSE
HVM52_MOUSE
HVM57_MOUSE
HVM60_MOUSE
IC1_MOUSE
IGG2B_MOUSE
IGHG3_MOUSE
IGHM_MOUSE
IGJ_MOUSE
IGKC_MOUSE
ITIH4_MOUSE
K22E_MOUSE
KNG1_MOUSE
KV2A6_MOUSE
KV2A7_MOUSE
KV3A1_MOUSE
KV3A4_MOUSE
KV3A8_MOUSE
KV3AC_MOUSE
KV3AI_MOUSE
KV3AM_MOUSE
KV4A1_MOUSE
KV5A1_MOUSE
KV5A3_MOUSE
KV5A4_MOUSE
KV5A6_MOUSE
KV5A7_MOUSE
KV5A9_MOUSE
KV6A7_MOUSE
KV6AB_MOUSE
LAC1_MOUSE
LOXL1_MOUSE
MASP1_MOUSE
MASP2_MOUSE

MBL1_MOUSE
MBL2_MOUSE
PLMN_MOUSE
PROP_MOUSE
PZP_MOUSE
SPA3K_MOUSE
THRB_MOUSE
TRFE_MOUSE
TSP1_MOUSE
TTHY_MOUSE
VTDB_MOUSE

Comparison of Proteins Associated with As Prepared and 4h Ball Milled SWCNTs
Accession Number

**Shared**	**As Prepared Only**	**4h Ball Milled Only**
A1AT3_MOUSE	A1AG1_MOUSE	A1AT2_MOUSE
A1AT4_MOUSE	APOH_MOUSE	APOB_MOUSE
ALBU_MOUSE	CERU_MOUSE	APOC4_MOUSE
APOA1_MOUSE	CFAB_MOUSE	FIBB_MOUSE
APOA2_MOUSE	F13B_MOUSE	HBB1_MOUSE
APOA4_MOUSE	FA12_MOUSE	K1C10_MOUSE
APOC1_MOUSE	FA5_MOUSE	K1C15_MOUSE
APOC2_MOUSE	FETUB_MOUSE	K2C6A_MOUSE
APOC3_MOUSE	HPT_MOUSE	K2C74_MOUSE
APOE_MOUSE	HVM00_MOUSE	KRA65_MOUSE
B2MG_MOUSE	IBP2_MOUSE	KRT86_MOUSE
C1QA_MOUSE	IGHG1_MOUSE	KT33A_MOUSE
C1QB_MOUSE	KNG1_MOUSE	KV3A3_MOUSE
C1QC_MOUSE	KV3A1_MOUSE	KV5AB_MOUSE
C1RA_MOUSE	KV3A4_MOUSE	LBP_MOUSE
C4BPA_MOUSE	KV3AB_MOUSE	LV1B_MOUSE
CD5L_MOUSE	KV5AC_MOUSE	PLF4_MOUSE
CFAH_MOUSE	KV6A7_MOUSE	ZEP2_MOUSE
CLUS_MOUSE	LAC1_MOUSE	
CO3_MOUSE	LAC2_MOUSE	
CO4B_MOUSE	LV1A_MOUSE	
CRP_MOUSE	MUG1_MOUSE	
CS1A_MOUSE	MUP2_MOUSE	
FETUA_MOUSE	PLMN_MOUSE	

FIBA_MOUSE
FIBG_MOUSE
FINC_MOUSE
GCAB_MOUSE
GELS_MOUSE
HA10_MOUSE
HBA_MOUSE
HEMO_MOUSE
HRG_MOUSE
HVM03_MOUSE
HVM05_MOUSE
HVM06_MOUSE
HVM09_MOUSE
HVM14_MOUSE
HVM16_MOUSE
HVM17_MOUSE
HVM35_MOUSE
HVM36_MOUSE
HVM51_MOUSE
HVM52_MOUSE
HVM57_MOUSE
HVM60_MOUSE
IC1_MOUSE
IGG2B_MOUSE
IGHG3_MOUSE
IGHM_MOUSE
IGJ_MOUSE
IGKC_MOUSE
ITIH4_MOUSE
K22E_MOUSE
KV2A6_MOUSE
KV2A7_MOUSE
KV3A8_MOUSE
KV3AC_MOUSE
KV3AI_MOUSE
KV3AM_MOUSE
KV4A1_MOUSE
KV5A1_MOUSE
KV5A3_MOUSE
KV5A4_MOUSE
KV5A6_MOUSE
RET4_MOUSE
SAA1_MOUSE
SEPP1_MOUSE
SPA3M_MOUSE

KV5A7_MOUSE
KV5A9_MOUSE
KV6AB_MOUSE
LOXL1_MOUSE
MASP1_MOUSE
MASP2_MOUSE
MBL1_MOUSE
MBL2_MOUSE
PROP_MOUSE
PZP_MOUSE
SPA3K_MOUSE
TETN_MOUSE
THRB_MOUSE
TRFE_MOUSE
TSP1_MOUSE
TTHY_MOUSE
VTDB_MOUSE

Comparison of Proteins Associated with As Prepared and 6h Ball Milled SWCNTs
Accession Number

**Shared**	**As Prepared Only**	**6h Ball Milled Only**
A1AT3_MOUSE	A1AG1_MOUSE	A1AT2_MOUSE
A1AT4_MOUSE	CERU_MOUSE	ACTB_MOUSE
ALBU_MOUSE	CFAB_MOUSE	APOB_MOUSE
APOA1_MOUSE	F13B_MOUSE	CO5_MOUSE
APOA2_MOUSE	FA12_MOUSE	FIBB_MOUSE
APOA4_MOUSE	FETUB_MOUSE	GPX3_MOUSE
APOC1_MOUSE	HPT_MOUSE	HBB1_MOUSE
APOC2_MOUSE	HVM00_MOUSE	K1C10_MOUSE
APOC3_MOUSE	HVM17_MOUSE	K1C13_MOUSE
APOE_MOUSE	IBP2_MOUSE	K1C14_MOUSE
APOH_MOUSE	IGHG1_MOUSE	K2C1_MOUSE
B2MG_MOUSE	K22E_MOUSE	K2C1B_MOUSE
C1QA_MOUSE	KNG1_MOUSE	K2C6A_MOUSE
C1QB_MOUSE	KV3A1_MOUSE	K2C79_MOUSE
C1QC_MOUSE	KV3A8_MOUSE	KRT35_MOUSE
C1RA_MOUSE	KV3AB_MOUSE	KV3A7_MOUSE
C4BPA_MOUSE	KV3AI_MOUSE	LBP_MOUSE
CD5L_MOUSE	LV1A_MOUSE	LV1B_MOUSE

CFAH_MOUSE
CLUS_MOUSE
CO3_MOUSE
CO4B_MOUSE
CRP_MOUSE
CS1A_MOUSE
FA5_MOUSE
FETUA_MOUSE
FIBA_MOUSE
FIBG_MOUSE
FINC_MOUSE
GCAB_MOUSE
GELS_MOUSE
HA10_MOUSE
HBA_MOUSE
HEMO_MOUSE
HRG_MOUSE
HVM03_MOUSE
HVM05_MOUSE
HVM06_MOUSE
HVM09_MOUSE
HVM14_MOUSE
HVM16_MOUSE
HVM35_MOUSE
HVM36_MOUSE
HVM51_MOUSE
HVM52_MOUSE
HVM57_MOUSE
HVM60_MOUSE
IC1_MOUSE
IGG2B_MOUSE
IGHG3_MOUSE
IGHM_MOUSE
IGJ_MOUSE
IGKC_MOUSE
ITIH4_MOUSE
KV2A6_MOUSE
KV2A7_MOUSE
KV3A4_MOUSE
KV3AC_MOUSE
KV3AM_MOUSE
MUP2_MOUSE
PLMN_MOUSE
RET4_MOUSE
SAA1_MOUSE
SEPP1_MOUSE
SPA3M_MOUSE
TETN_MOUSE
PLTP_MOUSE
QSOX1_MOUSE

KV4A1_MOUSE
KV5A1_MOUSE
KV5A3_MOUSE
KV5A4_MOUSE
KV5A6_MOUSE
KV5A7_MOUSE
KV5A9_MOUSE
KV5AC_MOUSE
KV6A7_MOUSE
KV6AB_MOUSE
LAC1_MOUSE
LAC2_MOUSE
LOXL1_MOUSE
MASP1_MOUSE
MASP2_MOUSE
MBL1_MOUSE
MBL2_MOUSE
MUG1_MOUSE
PROP_MOUSE
PZP_MOUSE
SPA3K_MOUSE
THRB_MOUSE
TRFE_MOUSE
TSP1_MOUSE
TTHY_MOUSE
VTDB_MOUSE

Comparison of Proteins Associated with As Prepared and 8h Ball Milled SWCNTs
Accession Number

**Shared**	**As Prepared Only**	**8h Ball Milled Only**
A1AT3_MOUSE	A1AG1_MOUSE	A1AT2_MOUSE
A1AT4_MOUSE	APOH_MOUSE	A1AT5_MOUSE
ALBU_MOUSE	C4BPA_MOUSE	ACTB_MOUSE
APOA1_MOUSE	CERU_MOUSE	APOB_MOUSE
APOA2_MOUSE	CFAB_MOUSE	FIBB_MOUSE
APOA4_MOUSE	F13B_MOUSE	K1C10_MOUSE
APOC1_MOUSE	FA12_MOUSE	K2C74_MOUSE
APOC2_MOUSE	FA5_MOUSE	KV5AB_MOUSE
APOC3_MOUSE	FETUB_MOUSE	LBP_MOUSE

APOE_MOUSE
B2MG_MOUSE
C1QA_MOUSE
C1QB_MOUSE
C1QC_MOUSE
C1RA_MOUSE
CD5L_MOUSE
CFAH_MOUSE
CLUS_MOUSE
CO3_MOUSE
CO4B_MOUSE
CRP_MOUSE
CS1A_MOUSE
FETUA_MOUSE
FIBA_MOUSE
FIBG_MOUSE
FINC_MOUSE
GELS_MOUSE
HA10_MOUSE
HBA_MOUSE
HEMO_MOUSE
HRG_MOUSE
HVM06_MOUSE
HVM09_MOUSE
HVM16_MOUSE
HVM35_MOUSE
HVM51_MOUSE
HVM52_MOUSE
HVM57_MOUSE
HVM60_MOUSE
IC1_MOUSE
IGHG3_MOUSE
IGHM_MOUSE
IGJ_MOUSE
IGKC_MOUSE
ITIH4_MOUSE
KNG1_MOUSE
KV2A6_MOUSE
KV2A7_MOUSE
KV3A4_MOUSE
KV3A8_MOUSE
GCAB_MOUSE
HPT_MOUSE
HVM00_MOUSE
HVM03_MOUSE
HVM05_MOUSE
HVM14_MOUSE
HVM17_MOUSE
HVM36_MOUSE
IBP2_MOUSE
IGG2B_MOUSE
IGHG1_MOUSE
K22E_MOUSE
KV3A1_MOUSE
KV3AI_MOUSE
KV5AC_MOUSE
KV6A7_MOUSE
LAC1_MOUSE
LAC2_MOUSE
LV1A_MOUSE
MUG1_MOUSE
MUP2_MOUSE
PLMN_MOUSE
RET4_MOUSE
SAA1_MOUSE
SEPP1_MOUSE
SPA3M_MOUSE
TETN_MOUSE
LV1C_MOUSE
PLTP_MOUSE
PRDX2_MOUSE

KV3AB_MOUSE
KV3AC_MOUSE
KV3AM_MOUSE
KV4A1_MOUSE
KV5A1_MOUSE
KV5A3_MOUSE
KV5A4_MOUSE
KV5A6_MOUSE
KV5A7_MOUSE
KV5A9_MOUSE
KV6AB_MOUSE
LOXL1_MOUSE
MASP1_MOUSE
MASP2_MOUSE
MBL1_MOUSE
MBL2_MOUSE
PROP_MOUSE
PZP_MOUSE
SPA3K_MOUSE
THRB_MOUSE
TRFE_MOUSE
TSP1_MOUSE
TTHY_MOUSE
VTDB_MOUSE

Supplemental Data Table 5. Normal Serum Relative Protein Quantification

Relative Quantification of Proteins Associated with SWCNTs Following Incubation in Normal Serum
All values are ratios of As Prepared SWCNTs : Ball Milled SWCNTs

Accession	Protein	2h Ball Milled	CV %	4h Ball Milled	CV %	6h Ball Milled	CV %	8h Ball Milled	CV %
A1AG1_MOUSE	Alpha-1-acid glycoprotein 1 OS=Mus musculus GN=Orm1 PE=1 SV=1	3.448	0.00	9.091	0.00				
A1AT2_MOUSE	Alpha-1-antitrypsin 1-2 OS=Mus musculus GN=Serpina1b PE=1 SV=2					1.370	0.00		
A1AT3_MOUSE	Alpha-1-antitrypsin 1-3 OS=Mus musculus GN=Serpina1c PE=1 SV=2					1.370	0.00		
ALBU_MOUSE	Serum albumin OS=Mus musculus GN=Alb PE=1 SV=3			2.174	19.23	1.852	13.55	1.961	8.70
APOA1_MOUSE	Apolipoprotein A-I OS=Mus musculus GN=Apoa1 PE=1 SV=2			2.083	6.42	2.703	16.20	2.083	18.81
APOA2_MOUSE	Apolipoprotein A-II OS=Mus musculus GN=Apoa2 PE=1 SV=2					1.408	0.00	2.000	0.00
APOA4_MOUSE	Apolipoprotein A-IV OS=Mus musculus GN=Apoa4 PE=1 SV=3	2.632	10.90	2.632	21.91	3.448	12.41	3.448	13.31
APOC1_MOUSE	Apolipoprotein C-I OS=Mus musculus GN=Apoc1 PE=1 SV=1							5.263	0.00
APOC3_MOUSE	Apolipoprotein C-III OS=Mus musculus GN=Apoc3 PE=1 SV=2					1.429	0.00		
APOE_MOUSE	Apolipoprotein E OS=Mus musculus GN=Apoe PE=1 SV=2							0.372	20.48
APOH_MOUSE	Beta-2-glycoprotein 1 OS=Mus musculus GN=Apoh PE=1 SV=1	5.000	2.95	5.000	12.05	7.692	0.00	5.263	16.55
C1QA_MOUSE	Complement C1q subcomponent subunit A OS=Mus musculus GN=C1qa PE=1 SV=2	0.322	5.14	0.245	0.00	0.239	2.55		
C1QB_MOUSE	Complement C1q subcomponent subunit B OS=Mus musculus GN=C1qb PE=1 SV=2	0.450	22.37	0.403	27.74	0.383	18.24	0.257	0.00
C1QC_MOUSE	Complement C1q subcomponent subunit C OS=Mus musculus GN=C1qc PE=1 SV=2	0.277	4.70	0.234	2.88	0.225	7.82		
C4BPA_MOUSE	C4b-binding protein OS=Mus musculus GN=C4bpa PE=1 SV=3							0.196	28.35
CD5L_MOUSE	CD5 antigen-like OS=Mus musculus GN=Cd5l PE=1 SV=3			0.676	0.00				
CFAB_MOUSE	Complement factor B OS=Mus musculus GN=Cfb PE=1 SV=2							0.266	0.00
CFAH_MOUSE	Complement factor H OS=Mus musculus GN=Cfh PE=1 SV=2	1.754	0.00	2.000	12.49	2.273	4.50	0.225	24.08
CFAI_MOUSE	Complement factor I OS=Mus musculus GN=Cfi PE=1 SV=3	1.695	0.00	2.439	0.00				
CLUS_MOUSE	Clusterin OS=Mus musculus GN=Clu PE=1 SV=1	3.704	11.82	2.174	8.92	2.326	1.28	1.961	0.00
CO3_MOUSE	Complement C3 OS=Mus musculus GN=C3 PE=1 SV=3	1.754	2.91	1.818	8.84	2.439	28.12	0.165	16.35
CO4B_MOUSE	Complement C4-B OS=Mus musculus GN=C4b PE=1 SV=3	2.326	0.00	2.857	21.59	3.704	28.80	0.255	41.38
CO5_MOUSE	Complement C5 OS=Mus musculus GN=C5 PE=1 SV=2							0.175	0.00
CO8A_MOUSE	Complement component C8 alpha chain OS=Mus musculus GN=C8a PE=1 SV=1							0.448	0.00
CRP_MOUSE	C-reactive protein OS=Mus musculus GN=Crp PE=1 SV=2	0.568	7.09	0.463	15.87	0.500	8.02		
CS1A_MOUSE	Complement C1s-A subcomponent OS=Mus musculus GN=C1sa PE=2 SV=2			0.476	10.40	0.521	0.00	0.515	0.00
EST1C_MOUSE	Carboxylesterase 1C OS=Mus musculus GN=Ces1c PE=1 SV=4					1.515	0.00	1.639	0.00
F13B_MOUSE	Coagulation factor XIII B chain OS=Mus musculus GN=F13b PE=1 SV=2							3.846	32.93
FA12_MOUSE	Coagulation factor XII OS=Mus musculus GN=F12 PE=1 SV=2	0.559	0.00			0.562	0.00		
FA5_MOUSE	Coagulation factor V OS=Mus musculus GN=F5 PE=1 SV=1	1.176	127.47			0.483	0.00	0.578	0.00
FETUA_MOUSE	Alpha-2-HS-glycoprotein OS=Mus musculus GN=Ahsg PE=1 SV=1	3.846	5.25	4.167	13.49	5.263	5.71	5.882	7.64
FIBA_MOUSE	Fibrinogen alpha chain OS=Mus musculus GN=Fga PE=1 SV=1			0.725	0.00			0.457	0.00
FIBG_MOUSE	Fibrinogen gamma chain OS=Mus musculus GN=Fgg PE=1 SV=1			0.806	0.00			0.513	0.00
FINC_MOUSE	Fibronectin OS=Mus musculus GN=Fn1 PE=1 SV=4	0.694	0.00	1.408	0.00	0.735	0.00	0.203	25.31
GELS_MOUSE	Gelsolin OS=Mus musculus GN=Gsn PE=1 SV=3	1.818	9.17	1.887	0.00	2.083	10.24	0.461	11.75
GUF1_MOUSE	Translation factor Guf1, mitochondrial OS=Mus musculus GN=Guf1 PE=1 SV=1	0.568	0.00						
HA10_MOUSE	H-2 class I histocompatibility antigen, Q10 alpha chain OS=Mus musculus GN=H2-Q10 PE=1 SV=3	4.167	7.81	5.263	6.33	2.041	142.15	4.545	16.87
HBB1_MOUSE	Hemoglobin subunit beta-1 OS=Mus musculus GN=Hbb-b1 PE=1 SV=2					1.887	0.00		
HEMO_MOUSE	Hemopexin OS=Mus musculus GN=Hpx PE=1 SV=2			2.381	0.60	2.857	20.27	4.348	12.51
HEPC_MOUSE	Hepcidin OS=Mus musculus GN=Hamp PE=2 SV=1	2.632	0.00	2.778	0.00	4.348	0.00		
HPT_MOUSE	Haptoglobin OS=Mus musculus GN=Hp PE=1 SV=1			0.457	0.00				
HRG_MOUSE	Histidine-rich glycoprotein OS=Mus musculus GN=Hrg PE=1 SV=2	3.226	8.80	3.846	21.05	3.571	14.73		
HVM00_MOUSE	Ig heavy chain V region OS=Mus musculus PE=1 SV=1	0.610	0.00	0.521	0.00			0.498	0.00

HVM06_MOUSE	Ig heavy chain V region 102 OS=Mus musculus PE=1 SV=1			0.595	0.00				
HVM09_MOUSE	Ig heavy chain V region 186-1 OS=Mus musculus PE=4 SV=2			0.513	0.00			0.437	0.00
HVM16_MOUSE	Ig heavy chain V region MOPC 21 (Fragment) OS=Mus musculus PE=1 SV=1			0.629	0.00				
HVM17_MOUSE	Ig heavy chain V region MOPC 47A OS=Mus musculus PE=1 SV=1			0.629	0.00				
HVM32_MOUSE	Ig heavy chain V-III region J606 OS=Mus musculus PE=1 SV=1			0.629	0.00				
HVM44_MOUSE	Ig heavy chain V region PJ14 OS=Mus musculus PE=1 SV=1			0.568	0.00				
HVM53_MOUSE	Ig heavy chain V region RF OS=Mus musculus PE=1 SV=1			0.629	0.00				
HVM54_MOUSE	Ig heavy chain V region 5-84 OS=Mus musculus PE=1 SV=1			0.629	0.00				
HVM56_MOUSE	Ig heavy chain V region 914 OS=Mus musculus PE=1 SV=1			0.629	0.00				
HVM57_MOUSE	Ig heavy chain V region 6.96 OS=Mus musculus PE=4 SV=1			0.629	0.00				
IGG2B_MOUSE	Ig gamma-2B chain C region OS=Mus musculus GN=Igh-3 PE=1 SV=3	0.662	0.00						
IGHG1_MOUSE	Ig gamma-1 chain C region secreted form OS=Mus musculus GN=Ighg1 PE=1 SV=1					2.632	0.00		
IGHG3_MOUSE	Ig gamma-3 chain C region OS=Mus musculus PE=1 SV=2	0.495	14.42	0.402	24.77	0.380	0.00	0.325	16.59
IGHM_MOUSE	Ig mu chain C region OS=Mus musculus GN=Ighm PE=1 SV=2	0.420	32.50	0.431	43.05	0.336	7.33	0.676	66.09
IGJ_MOUSE	Immunoglobulin J chain OS=Mus musculus GN=Jchain PE=1 SV=4	0.625	0.00	0.467	23.20	0.439	0.00		
IGKC_MOUSE	Ig kappa chain C region OS=Mus musculus PE=1 SV=1			0.420	0.00				
ITIH4_MOUSE	Inter alpha-trypsin inhibitor, heavy chain 4 OS=Mus musculus GN=Itih4 PE=1 SV=2	2.941	17.17	3.333	4.51	3.333	16.98	0.529	0.00
KISS1_MOUSE	Metastasis-suppressor KiSS-1 OS=Mus musculus GN=Kiss1 PE=1 SV=2			0.452	0.00				
KNG1_MOUSE	Kininogen-1 OS=Mus musculus GN=Kng1 PE=1 SV=1	2.941	7.07	3.571	11.81	4.545	16.25	2.222	11.67
KV2A7_MOUSE	Ig kappa chain V-II region 26-10 OS=Mus musculus PE=1 SV=1	0.694	0.00			0.637	0.00		
KV3AI_MOUSE	Ig kappa chain V-III region PC 6684 OS=Mus musculus PE=1 SV=1							2.381	0.00
KV5A1_MOUSE	Ig kappa chain V19-17 OS=Mus musculus GN=Igk-V19-17 PE=1 SV=1			0.448	0.00				
KV5A4_MOUSE	Ig kappa chain V-V region MOPC 149 OS=Mus musculus PE=1 SV=1							2.857	0.00
KV5A6_MOUSE	Ig kappa chain V-V region L6 (Fragment) OS=Mus musculus PE=4 SV=1			0.613	0.00			0.625	0.00
KV5AA_MOUSE	Ig kappa chain V-V region MOPC 173 OS=Mus musculus PE=1 SV=1							2.381	0.00
KV5AC_MOUSE	Ig kappa chain V-V region HP 93G7 OS=Mus musculus PE=1 SV=1							2.381	0.00
KV5AF_MOUSE	Ig kappa chain V-V region HP 91A3 OS=Mus musculus PE=1 SV=1							2.381	0.00
KV5AG_MOUSE	Ig kappa chain V-V regions OS=Mus musculus PE=1 SV=1							2.381	0.00
MASP2_MOUSE	Mannan-binding lectin serine protease 2 OS=Mus musculus GN=Masp2 PE=1 SV=1							0.337	13.87
MATN2_MOUSE	Matrilin-2 OS=Mus musculus GN=Matn2 PE=2 SV=2			2.326	0.00				
MBL1_MOUSE	Mannose-binding protein A OS=Mus musculus GN=Mbl1 PE=1 SV=1			0.526	0.00			0.219	9.04
MBL2_MOUSE	Mannose-binding protein C OS=Mus musculus GN=Mbl2 PE=1 SV=2			0.658	0.00			0.488	0.00
MUP1_MOUSE	Major urinary protein 1 OS=Mus musculus GN=Mup1 PE=1 SV=1	7.143	0.00	12.500	0.00	14.286	5.57		
MUP2_MOUSE	Major urinary protein 2 OS=Mus musculus GN=Mup2 PE=1 SV=1	6.250	0.00			14.286	3.08		
NPY_MOUSE	Pro-neuropeptide Y OS=Mus musculus GN=Npy PE=1 SV=2	1.429	0.00	2.000	0.00	2.326	0.00		
PEDF_MOUSE	Pigment epithelium-derived factor OS=Mus musculus GN=Serpinf1 PE=1 SV=2							0.230	0.00
PLF4_MOUSE	Platelet factor 4 OS=Mus musculus GN=Pf4 PE=1 SV=1	0.465	0.00			0.355	0.00	0.510	0.00
PLMN_MOUSE	Plasminogen OS=Mus musculus GN=Plg PE=1 SV=3							0.303	16.25
PROP_MOUSE	Properdin OS=Mus musculus GN=Cfp PE=2 SV=2	0.538	0.00					0.306	3.86
PZP_MOUSE	Pregnancy zone protein OS=Mus musculus GN=Pzp PE=1 SV=3	0.667	0.00	2.326	0.00	1.163	44.45	1.786	0.00
SEPP1_MOUSE	Selenoprotein P OS=Mus musculus GN=Sepp1 PE=1 SV=3							0.565	0.00
THRB_MOUSE	Prothrombin OS=Mus musculus GN=F2 PE=1 SV=1	3.448	11.45	3.226	0.00	4.762	21.43	0.379	0.00
TRFE_MOUSE	Serotransferrin OS=Mus musculus GN=Tf PE=1 SV=1					2.041	5.49	3.333	1.95
TSP1_MOUSE	Thrombospondin-1 OS=Mus musculus GN=Thbs1 PE=1 SV=1	0.549	0.00	0.478	9.07			0.383	11.84
TTHY_MOUSE	Transthyretin OS=Mus musculus GN=Ttr PE=1 SV=1	4.000	4.71	5.263	0.00	5.882	0.00	7.143	0.00

Supplemental Data Table 6. Lipid Serum Relative Quantification

Relative Quantification of Proteins Associated with SWCNTs Following Incubation in Hyperlipidemic Serum
All values are ratios of As Prepared SWCNTs : Ball Milled SWCNTs

Accession	Protein	2h Ball Milled	CV %	4h Ball Milled	CV %	6h Ball Milled	CV %	8h Ball Milled	CV %
A1AG1_MOUSE	Alpha-1-acid glycoprotein 1 OS=Mus musculus GN=Orm1 PE=1 SV=1	4.762	0.00						
A1AT2_MOUSE	Alpha-1-antitrypsin 1-2 OS=Mus musculus GN=Serpina1b PE=1 SV=2					2.222	0.00	2.273	0.00
A1AT3_MOUSE	Alpha-1-antitrypsin 1-3 OS=Mus musculus GN=Serpina1c PE=1 SV=2					2.273	0.00	2.703	0.00
A1AT4_MOUSE	Alpha-1-antitrypsin 1-4 OS=Mus musculus GN=Serpina1d PE=1 SV=1								
A1AT5_MOUSE	Alpha-1-antitrypsin 1-5 OS=Mus musculus GN=Serpina1e PE=1 SV=1								
A2AP_MOUSE	Alpha-2-antiplasmin OS=Mus musculus GN=Serpinf2 PE=1 SV=1								
ACTB_MOUSE	Actin, cytoplasmic 1 OS=Mus musculus GN=Actb PE=1 SV=1								
ALBU_MOUSE	Serum albumin OS=Mus musculus GN=Alb PE=1 SV=3	1.754	7.99			2.703	18.19	2.703	28.08
ANT3_MOUSE	Antithrombin-III OS=Mus musculus GN=Serpinc1 PE=1 SV=1								
APOA1_MOUSE	Apolipoprotein A-I OS=Mus musculus GN=Apoa1 PE=1 SV=2	2.941	13.19	1.852	0.00	5.263	13.52	5.556	9.91
APOA2_MOUSE	Apolipoprotein A-II OS=Mus musculus GN=Apoa2 PE=1 SV=2					2.381	0.00	2.041	0.00
APOA4_MOUSE	Apolipoprotein A-IV OS=Mus musculus GN=Apoa4 PE=1 SV=3	4.545	11.64			7.143	21.45	7.143	7.22
APOB_MOUSE	Apolipoprotein B-100 OS=Mus musculus GN=Apob PE=1 SV=1								
APOC1_MOUSE	Apolipoprotein C-I OS=Mus musculus GN=Apoc1 PE=1 SV=1	2.564	0.00			3.846	13.74	3.226	21.93
APOC2_MOUSE	Apolipoprotein C-II OS=Mus musculus GN=Apoc2 PE=2 SV=1	4.545	0.00						
APOC3_MOUSE	Apolipoprotein C-III OS=Mus musculus GN=Apoc3 PE=1 SV=2					2.778	3.04	2.174	0.00
APOC4_MOUSE	Apolipoprotein C-IV OS=Mus musculus GN=Apoc4 PE=1 SV=1								
APOE_MOUSE	Apolipoprotein E OS=Mus musculus GN=Apoe PE=1 SV=2	1.852	3.03			2.381	12.27	2.381	15.24
APOH_MOUSE	Beta-2-glycoprotein 1 OS=Mus musculus GN=Apoh PE=1 SV=1			5.882	0.00				
ARAF_MOUSE	Serine/threonine-protein kinase A-Raf OS=Mus musculus GN=Araf PE=1 SV=2	2.381	0.00			2.500	0.00	2.326	0.00
ARRD2_MOUSE	Arrestin domain-containing protein 2 OS=Mus musculus GN=Arrdc2 PE=2 SV=1								
B2MG_MOUSE	Beta-2-microglobulin OS=Mus musculus GN=B2m PE=1 SV=2								
C1QA_MOUSE	Complement C1q subcomponent subunit A OS=Mus musculus GN=C1qa PE=1 SV=2			0.246	0.00				
C1QB_MOUSE	Complement C1q subcomponent subunit B OS=Mus musculus GN=C1qb PE=1 SV=2			0.309	23.29	1.471	0.00	1.961	0.65
C1QC_MOUSE	Complement C1q subcomponent subunit C OS=Mus musculus GN=C1qc PE=1 SV=2			0.226	0.00			1.205	0.00
C1RA_MOUSE	Complement C1r-A subcomponent OS=Mus musculus GN=C1ra PE=1 SV=1					2.439	0.00	3.333	14.92
C4BPA_MOUSE	C4b-binding protein OS=Mus musculus GN=C4bpa PE=1 SV=3	6.667	0.00					6.250	0.00
CD5L_MOUSE	CD5 antigen-like OS=Mus musculus GN=Cd5l PE=1 SV=3							2.632	2.55
CERU_MOUSE	Ceruloplasmin OS=Mus musculus GN=Cp PE=1 SV=2								
CFAB_MOUSE	Complement factor B OS=Mus musculus GN=Cfb PE=1 SV=2								
CFAH_MOUSE	Complement factor H OS=Mus musculus GN=Cfh PE=1 SV=2	4.000	10.85			4.000	23.84	6.250	25.75
CL056_MOUSE	Uncharacterized protein C12orf56 homolog OS=Mus musculus PE=2 SV=1								
CLUS_MOUSE	Clusterin OS=Mus musculus GN=Clu PE=1 SV=1	3.125	0.00	4.000	0.00	5.556	11.79	4.167	5.51
CO3_MOUSE	Complement C3 OS=Mus musculus GN=C3 PE=1 SV=3	3.333	42.56			3.125	15.13	4.000	27.28
CO4B_MOUSE	Complement C4-B OS=Mus musculus GN=C4b PE=1 SV=3							2.632	0.00
CO5_MOUSE	Complement C5 OS=Mus musculus GN=C5 PE=1 SV=2	1.786	0.00			2.632	0.00		
CO9_MOUSE	Complement component C9 OS=Mus musculus GN=C9 PE=1 SV=2								
CRP_MOUSE	C-reactive protein OS=Mus musculus GN=Crp PE=1 SV=2	0.418	0.00	0.518	10.77				
CS1A_MOUSE	Complement C1s-A subcomponent OS=Mus musculus GN=C1sa PE=2 SV=2	2.632	0.00			2.941	4.12	2.857	3.69
DI3L2_MOUSE	DIS3-like exonuclease 2 OS=Mus musculus GN=Dis3l2 PE=1 SV=1							4.762	0.00
ECM1_MOUSE	Extracellular matrix protein 1 OS=Mus musculus GN=Ecm1 PE=1 SV=2								
F13B_MOUSE	Coagulation factor XIII B chain OS=Mus musculus GN=F13b PE=1 SV=2							4.762	0.00
FA12_MOUSE	Coagulation factor XII OS=Mus musculus GN=F12 PE=1 SV=2								
FA5_MOUSE	Coagulation factor V OS=Mus musculus GN=F5 PE=1 SV=1								

FETUA_MOUSE	Alpha-2-HS-glycoprotein OS=Mus musculus GN=Ahsg PE=1 SV=1	3.704	0.00	3.333	0.00	5.556	0.00	6.667	0.00
FETUB_MOUSE	Fetuin-B OS=Mus musculus GN=Fetub PE=1 SV=1								
FIBA_MOUSE	Fibrinogen alpha chain OS=Mus musculus GN=Fga PE=1 SV=1	2.326	0.00			2.632	0.00	0.667	0.00
FIBB_MOUSE	Fibrinogen beta chain OS=Mus musculus GN=Fgb PE=1 SV=1								
FIBG_MOUSE	Fibrinogen gamma chain OS=Mus musculus GN=Fgg PE=1 SV=1					0.295	0.00		
FINC_MOUSE	Fibronectin OS=Mus musculus GN=Fn1 PE=1 SV=4					1.852	10.19		
G3P_MOUSE	Glyceraldehyde-3-phosphate dehydrogenase OS=Mus musculus GN=Gapdh PE=1 SV=2								
GCAB_MOUSE	Ig gamma-2A chain C region secreted form OS=Mus musculus PE=1 SV=1								
GELS_MOUSE	Gelsolin OS=Mus musculus GN=Gsn PE=1 SV=3	2.222	13.66			2.941	16.54	2.703	25.38
GEN_MOUSE	Flap endonuclease GEN homolog 1 OS=Mus musculus GN=Gen1 PE=1 SV=2								
GPX3_MOUSE	Glutathione peroxidase 3 OS=Mus musculus GN=Gpx3 PE=1 SV=2								
GUF1_MOUSE	Translation factor Guf1, mitochondrial OS=Mus musculus GN=Guf1 PE=1 SV=1			0.515	0.00				
HA10_MOUSE	H-2 class I histocompatibility antigen, Q10 alpha chain OS=Mus musculus GN=H2-Q10 PE=1 SV=3	2.703	13.28	4.348	12.32	3.448	0.00	3.571	5.60
HBA_MOUSE	Hemoglobin subunit alpha OS=Mus musculus GN=Hba PE=1 SV=2								
HBB1_MOUSE	Hemoglobin subunit beta-1 OS=Mus musculus GN=Hbb-b1 PE=1 SV=2								
HEMO_MOUSE	Hemopexin OS=Mus musculus GN=Hpx PE=1 SV=2	1.563	0.00			3.030	5.81	3.704	27.10
HEPC_MOUSE	Hepcidin OS=Mus musculus GN=Hamp PE=2 SV=1								
HPT_MOUSE	Haptoglobin OS=Mus musculus GN=Hp PE=1 SV=1	2.703	0.00						
HRG_MOUSE	Histidine-rich glycoprotein OS=Mus musculus GN=Hrg PE=1 SV=2	5.882	9.18	4.348	13.02	10.000	0.00	10.000	6.63
HVM00_MOUSE	Ig heavy chain V region OS=Mus musculus PE=1 SV=1			0.474	0.00			2.632	0.00
HVM03_MOUSE	Ig heavy chain V region 36-65 OS=Mus musculus PE=1 SV=1							2.941	0.00
HVM05_MOUSE	Ig heavy chain V region 3 OS=Mus musculus GN=Ighv1-61 PE=1 SV=1								
HVM06_MOUSE	Ig heavy chain V region 102 OS=Mus musculus PE=1 SV=1							1.471	0.00
HVM10_MOUSE	Ig heavy chain V region 1-62-3 OS=Mus musculus GN=Ighv1-62-3 PE=4 SV=2								
HVM14_MOUSE	Ig heavy chain V region 108A OS=Mus musculus GN=Igh-VJ558 PE=4 SV=1								
HVM16_MOUSE	Ig heavy chain V region MOPC 21 (Fragment) OS=Mus musculus PE=1 SV=1								
HVM17_MOUSE	Ig heavy chain V region MOPC 47A OS=Mus musculus PE=1 SV=1								
HVM28_MOUSE	Ig heavy chain V-III region U61 OS=Mus musculus PE=1 SV=1								
HVM36_MOUSE	Ig heavy chain V region 441 OS=Mus musculus PE=4 SV=1								
HVM51_MOUSE	Ig heavy chain V region AC38 205.12 OS=Mus musculus PE=1 SV=1								
HVM52_MOUSE	Ig heavy chain V region VH558 A1/A4 OS=Mus musculus GN=Gm5629 PE=2 SV=1								
HVM53_MOUSE	Ig heavy chain V region RF OS=Mus musculus PE=1 SV=1								
HVM57_MOUSE	Ig heavy chain V region 6.96 OS=Mus musculus PE=4 SV=1								
HVM60_MOUSE	Ig heavy chain V region 3-6 OS=Mus musculus GN=Ighv3-6 PE=1 SV=1							1.786	11.50
IBP2_MOUSE	Insulin-like growth factor-binding protein 2 OS=Mus musculus GN=Igfbp2 PE=2 SV=2								
IBP4_MOUSE	Insulin-like growth factor-binding protein 4 OS=Mus musculus GN=Igfbp4 PE=1 SV=2								
IBP6_MOUSE	Insulin-like growth factor-binding protein 6 OS=Mus musculus GN=Igfbp6 PE=2 SV=2								
IC1_MOUSE	Plasma protease C1 inhibitor OS=Mus musculus GN=Serping1 PE=1 SV=3								
IGG2B_MOUSE	Ig gamma-2B chain C region OS=Mus musculus GN=Igh-3 PE=1 SV=3								
IGHG1_MOUSE	Ig gamma-1 chain C region secreted form OS=Mus musculus GN=Ighg1 PE=1 SV=1								
IGHG3_MOUSE	Ig gamma-3 chain C region OS=Mus musculus PE=1 SV=2								
IGHM_MOUSE	Ig mu chain C region OS=Mus musculus GN=Ighm PE=1 SV=2	1.205	123.36	0.735	0.00	2.564	0.00	2.632	29.61
IGJ_MOUSE	Immunoglobulin J chain OS=Mus musculus GN=Jchain PE=1 SV=4			0.369	0.00	2.703	0.00	2.941	0.00
IGKC_MOUSE	Ig kappa chain C region OS=Mus musculus PE=1 SV=1					1.818	0.00	3.030	0.00
ITIH4_MOUSE	Inter alpha-trypsin inhibitor, heavy chain 4 OS=Mus musculus GN=Itih4 PE=1 SV=2	3.704	19.17	2.326	0.00	5.556	8.46	4.000	19.71
K1C10_MOUSE	Keratin, type I cytoskeletal 10 OS=Mus musculus GN=Krt10 PE=1 SV=3								
K1C13_MOUSE	Keratin, type I cytoskeletal 13 OS=Mus musculus GN=Krt13 PE=1 SV=2							2.222	0.00
K1C17_MOUSE	Keratin, type I cytoskeletal 17 OS=Mus musculus GN=Krt17 PE=1 SV=3							2.222	0.00
K1H1_MOUSE	Keratin, type I cuticular Ha1 OS=Mus musculus GN=Krt31 PE=1 SV=2								
K22E_MOUSE	Keratin, type II cytoskeletal 2 epidermal OS=Mus musculus GN=Krt2 PE=1 SV=1								

K2C1_MOUSE	Keratin, type II cytoskeletal 1 OS=Mus musculus GN=Krt1 PE=1 SV=4	2.381	0.00			2.500	0.00	2.326	0.00
K2C1B_MOUSE	Keratin, type II cytoskeletal 1b OS=Mus musculus GN=Krt77 PE=1 SV=1								
K2C6A_MOUSE	Keratin, type II cytoskeletal 6A OS=Mus musculus GN=Krt6a PE=1 SV=3								
K2C79_MOUSE	Keratin, type II cytoskeletal 79 OS=Mus musculus GN=Krt79 PE=1 SV=2								
KNG1_MOUSE	Kininogen-1 OS=Mus musculus GN=Kng1 PE=1 SV=1	3.846	0.00	3.125	0.00	5.556	8.56	7.143	19.26
KRA31_MOUSE	Keratin-associated protein 3-1 OS=Mus musculus GN=Krtap3-1 PE=3 SV=1								
KRA65_MOUSE	Keratin-associated protein 6-5 OS=Mus musculus GN=Krtap6-5 PE=2 SV=1								
KRT34_MOUSE	Keratin, type I cuticular Ha4 OS=Mus musculus GN=Krt34 PE=2 SV=1								
KRT81_MOUSE	Keratin, type II cuticular Hb1 OS=Mus musculus GN=Krt81 PE=2 SV=2								
KT33A_MOUSE	Keratin, type I cuticular Ha3-I OS=Mus musculus GN=Krt33a PE=1 SV=1								
KV2A5_MOUSE	Ig kappa chain V-II region 17S29.1 OS=Mus musculus PE=1 SV=1								
KV2A6_MOUSE	Ig kappa chain V-II region 7S34.1 OS=Mus musculus PE=1 SV=1	2.703	0.00			2.174	0.00	3.125	0.00
KV2A7_MOUSE	Ig kappa chain V-II region 26-10 OS=Mus musculus PE=1 SV=1	2.439	0.00	0.395	0.00	2.174	0.00	3.333	11.18
KV3A1_MOUSE	Ig kappa chain V-III region PC 2880/PC 1229 OS=Mus musculus PE=1 SV=1	2.439	3.14			2.703	17.92	3.125	3.24
KV3A4_MOUSE	Ig kappa chain V-III region 50S10.1 OS=Mus musculus PE=1 SV=1	2.439	3.14			2.857	20.89	3.125	3.24
KV3A8_MOUSE	Ig kappa chain V-III region PC 3741/TEPC 111 OS=Mus musculus PE=1 SV=1	3.030	20.18			3.448	4.28	3.846	25.86
KV3AB_MOUSE	Ig kappa chain V-III region PC 4050 OS=Mus musculus PE=1 SV=1	2.500	0.41			3.125	10.54	3.226	5.41
KV3AC_MOUSE	Ig kappa chain V-III region CBPC 101 OS=Mus musculus PE=1 SV=1	2.703	8.37			3.448	0.00	3.030	0.00
KV3AE_MOUSE	Ig kappa chain V-III region PC 7183 OS=Mus musculus PE=1 SV=1	2.703	8.37			3.448	0.00	3.030	0.00
KV3AI_MOUSE	Ig kappa chain V-III region PC 6684 OS=Mus musculus PE=1 SV=1	2.500	0.00			3.448	0.00	3.030	0.00
KV3AM_MOUSE	Ig kappa chain V-III region PC 2154 OS=Mus musculus PE=1 SV=1								
KV4A1_MOUSE	Ig kappa chain V-IV region S107B OS=Mus musculus PE=4 SV=1	1.724	0.00			2.778	0.00	3.030	0.00
KV5A1_MOUSE	Ig kappa chain V19-17 OS=Mus musculus GN=Igk-V19-17 PE=1 SV=1							2.857	0.00
KV5A3_MOUSE	Ig kappa chain V-V region K2 (Fragment) OS=Mus musculus PE=1 SV=1							2.222	0.00
KV5A4_MOUSE	Ig kappa chain V-V region MOPC 149 OS=Mus musculus PE=1 SV=1								
KV5A6_MOUSE	Ig kappa chain V-V region L6 (Fragment) OS=Mus musculus PE=4 SV=1								
KV5A7_MOUSE	Ig kappa chain V-V region MOPC 41 OS=Mus musculus GN=Gm5571 PE=1 SV=1								
KV5A9_MOUSE	Ig kappa chain V-V region L7 (Fragment) OS=Mus musculus GN=Gm10881 PE=1 SV=1							3.704	0.00
KV5AB_MOUSE	Ig kappa chain V-V region HP R16.7 OS=Mus musculus PE=1 SV=1					2.439	0.00	3.448	0.00
KV6A7_MOUSE	Ig kappa chain V-VI region NQ2-48.2.2 OS=Mus musculus PE=2 SV=1								
KV6AB_MOUSE	Ig kappa chain V-VI region NQ2-6.1 OS=Mus musculus PE=2 SV=1								
LAC1_MOUSE	Ig lambda-1 chain C region OS=Mus musculus PE=1 SV=1	3.704	0.00			4.167	0.00		
LAC2_MOUSE	Ig lambda-2 chain C region OS=Mus musculus GN=Iglc2 PE=1 SV=1								
LBP_MOUSE	Lipopolysaccharide-binding protein OS=Mus musculus GN=Lbp PE=1 SV=2								
LOXL1_MOUSE	Lysyl oxidase homolog 1 OS=Mus musculus GN=Loxl1 PE=2 SV=3	3.704	11.79			3.571	2.67	3.030	7.88
LV1B_MOUSE	Ig lambda-1 chain V regions MOPC 104E/RPC20/J558/S104 OS=Mus musculus PE=1 SV=1							3.226	0.00
MASP1_MOUSE	Mannan-binding lectin serine protease 1 OS=Mus musculus GN=Masp1 PE=1 SV=2					2.703	0.00		
MASP2_MOUSE	Mannan-binding lectin serine protease 2 OS=Mus musculus GN=Masp2 PE=1 SV=1					2.500	7.88	3.704	0.00
MATN2_MOUSE	Matrilin-2 OS=Mus musculus GN=Matn2 PE=2 SV=2	5.556	0.00			3.333	0.00	4.762	0.00
MBL1_MOUSE	Mannose-binding protein A OS=Mus musculus GN=Mbl1 PE=1 SV=1	14.286	329.19	3.571	0.00	8.333	141.90	16.667	182.26
MBL2_MOUSE	Mannose-binding protein C OS=Mus musculus GN=Mbl2 PE=1 SV=2					3.333	0.00	3.226	17.25
MUG1_MOUSE	Murinoglobulin-1 OS=Mus musculus GN=Mug1 PE=1 SV=3	2.439	0.00						
MUP2_MOUSE	Major urinary protein 2 OS=Mus musculus GN=Mup2 PE=1 SV=1								
MYH14_MOUSE	Myosin-14 OS=Mus musculus GN=Myh14 PE=1 SV=1								
PLF4_MOUSE	Platelet factor 4 OS=Mus musculus GN=Pf4 PE=1 SV=1			0.403	0.00			1.724	0.00
PLMN_MOUSE	Plasminogen OS=Mus musculus GN=Plg PE=1 SV=3	2.041	0.00			1.961	0.00		
PLTP_MOUSE	Phospholipid transfer protein OS=Mus musculus GN=Pltp PE=1 SV=1								
PRDX2_MOUSE	Peroxiredoxin-2 OS=Mus musculus GN=Prdx2 PE=1 SV=3								
PROP_MOUSE	Properdin OS=Mus musculus GN=Cfp PE=2 SV=2	3.226	0.00			2.564	0.00	6.667	0.00
PZP_MOUSE	Pregnancy zone protein OS=Mus musculus GN=Pzp PE=1 SV=3	0.617	0.00						

QSOX1_MOUSE	Sulfhydryl oxidase 1 OS=Mus musculus GN=Qsox1 PE=1 SV=1								
RET4_MOUSE	Retinol-binding protein 4 OS=Mus musculus GN=Rbp4 PE=1 SV=2	5.556	0.00			6.667	0.00	7.692	0.00
RFIP2_MOUSE	Rab11 family-interacting protein 2 OS=Mus musculus GN=Rab11fip2 PE=1 SV=1								
SAA1_MOUSE	Serum amyloid A-1 protein OS=Mus musculus GN=Saa1 PE=1 SV=2								
SEPP1_MOUSE	Selenoprotein P OS=Mus musculus GN=Sepp1 PE=1 SV=3								
SLF2_MOUSE	SMC5-SMC6 complex localization factor protein 2 OS=Mus musculus GN=Slf2 PE=2 SV=2								
SPA3K_MOUSE	Serine protease inhibitor A3K OS=Mus musculus GN=Serpina3k PE=1 SV=2	3.030	0.00			2.273	0.00	2.128	
SPA3M_MOUSE	Serine protease inhibitor A3M OS=Mus musculus GN=Serpina3m PE=1 SV=2	3.030	0.00			2.273	0.00	2.632	20.35
SPA3N_MOUSE	Serine protease inhibitor A3N OS=Mus musculus GN=Serpina3n PE=1 SV=1	3.030	0.00						0.00
SPTA1_MOUSE	Spectrin alpha chain, erythrocytic 1 OS=Mus musculus GN=Spta1 PE=1 SV=3								
TBPL1_MOUSE	TATA box-binding protein-like protein 1 OS=Mus musculus GN=Tbpl1 PE=1 SV=1								
TETN_MOUSE	Tetranectin OS=Mus musculus GN=Clec3b PE=1 SV=2								
THRB_MOUSE	Prothrombin OS=Mus musculus GN=F2 PE=1 SV=1	2.703	0.00	3.125	0.00	4.000	0.00	4.545	
TOPB1_MOUSE	DNA topoisomerase 2-binding protein 1 OS=Mus musculus GN=Topbp1 PE=1 SV=2								0.00
TRFE_MOUSE	Serotransferrin OS=Mus musculus GN=Tf PE=1 SV=1					1.852	18.09	2.326	
TSP1_MOUSE	Thrombospondin-1 OS=Mus musculus GN=Thbs1 PE=1 SV=1							2.778	22.31
TTHY_MOUSE	Transthyretin OS=Mus musculus GN=Ttr PE=1 SV=1	4.545	41.43			6.667	24.31	7.692	8.17
TYRP2_MOUSE	L-dopachrome tautomerase OS=Mus musculus GN=Dct PE=1 SV=2								11.56
VTDB_MOUSE	Vitamin D-binding protein OS=Mus musculus GN=Gc PE=1 SV=2	1.538	0.00						
VTNC_MOUSE	Vitronectin OS=Mus musculus GN=Vtn PE=1 SV=2								
ZEP2_MOUSE	Transcription factor HIVEP2 OS=Mus musculus GN=Hivep2 PE=1 SV=1								

Supplemental Data Table 7. Comparison of Proteins found to Bind each SWCNT in either Normal or Hyperlipidemic Serum

Comparison of Proteins Associated with SWCNTs in Normal or Hyperlipidemic Serum
Accession Number

As Prepared SWCNTs

In Common	Normal Serum Only	Hyperlipidemic Serum Only
A1AG1_MOUSE	A1AT4_MOUSE	A1AT2_MOUSE
A1AT3_MOUSE	GCAB_MOUSE	ACTB_MOUSE
ALBU_MOUSE	HBA_MOUSE	ANT3_MOUSE
APOA1_MOUSE	HVM03_MOUSE	CFAD_MOUSE
APOA2_MOUSE	HVM09_MOUSE	CFAI_MOUSE
APOA4_MOUSE	HVM14_MOUSE	ECM1_MOUSE
APOC1_MOUSE	HVM35_MOUSE	G3P_MOUSE
APOC2_MOUSE	HVM36_MOUSE	GCAM_MOUSE
APOC3_MOUSE	HVM52_MOUSE	HBB1_MOUSE
APOE_MOUSE	K22E_MOUSE	HEPC_MOUSE
APOH_MOUSE	KV3A1_MOUSE	HVM13_MOUSE
B2MG_MOUSE	KV3AC_MOUSE	HVM21_MOUSE
C1QA_MOUSE	KV5AC_MOUSE	HVM32_MOUSE
C1QB_MOUSE	LAC1_MOUSE	HVM37_MOUSE
C1QC_MOUSE	LAC2_MOUSE	HVM45_MOUSE
C1RA_MOUSE	LV1A_MOUSE	HVM53_MOUSE
C4BPA_MOUSE	SPA3M_MOUSE	HVM54_MOUSE
CD5L_MOUSE		HVM56_MOUSE
CERU_MOUSE		IBP4_MOUSE
CFAB_MOUSE		KV2A5_MOUSE
CFAH_MOUSE		KV3AG_MOUSE
CLUS_MOUSE		KV3AJ_MOUSE
CO3_MOUSE		KV5AB_MOUSE
CO4B_MOUSE		KV5AG_MOUSE
CRP_MOUSE		KV6A5_MOUSE
CS1A_MOUSE		LV1B_MOUSE
F13B_MOUSE		MUP1_MOUSE
FA12_MOUSE		NPY_MOUSE
FA5_MOUSE		QSOX1_MOUSE
FETUA_MOUSE		SAMP_MOUSE
FETUB_MOUSE		SBSN_MOUSE
FIBA_MOUSE		VTNC_MOUSE
FIBG_MOUSE		

FINC_MOUSE
GELS_MOUSE
HA10_MOUSE
HEMO_MOUSE
HPT_MOUSE
HRG_MOUSE
HVM00_MOUSE
HVM05_MOUSE
HVM06_MOUSE
HVM16_MOUSE
HVM17_MOUSE
HVM51_MOUSE
HVM57_MOUSE
HVM60_MOUSE
IBP2_MOUSE
IC1_MOUSE
IGG2B_MOUSE
IGHG1_MOUSE
IGHG3_MOUSE
IGHM_MOUSE
IGJ_MOUSE
IGKC_MOUSE
ITIH4_MOUSE
KNG1_MOUSE
KV2A6_MOUSE
KV2A7_MOUSE
KV3A4_MOUSE
KV3A8_MOUSE
KV3AB_MOUSE
KV3AI_MOUSE
KV3AM_MOUSE
KV4A1_MOUSE
KV5A1_MOUSE
KV5A3_MOUSE
KV5A4_MOUSE
KV5A6_MOUSE
KV5A7_MOUSE
KV5A9_MOUSE
KV6A7_MOUSE
KV6AB_MOUSE
LOXL1_MOUSE

MASP1_MOUSE
MASP2_MOUSE
MBL1_MOUSE
MBL2_MOUSE
MUG1_MOUSE
MUP2_MOUSE
PLMN_MOUSE
PROP_MOUSE
PZP_MOUSE
RET4_MOUSE
SAA1_MOUSE
SEPP1_MOUSE
SPA3K_MOUSE
TETN_MOUSE
THRB_MOUSE
TRFE_MOUSE
TSP1_MOUSE
TTHY_MOUSE
VTDB_MOUSE

2h Ball Milled SWCNTs

**Shared**	**Normal Serum Only**	**Hyperlipidemic Serum Only**
A1AG1_MOUSE	ACTB_MOUSE	A1AT4_MOUSE
A1AT2_MOUSE	BPIA2_MOUSE	A2AP_MOUSE
A1AT3_MOUSE	CFAB_MOUSE	ACTBL_MOUSE
ALBU_MOUSE	CFAI_MOUSE	APOB_MOUSE
APOA1_MOUSE	FA5_MOUSE	B2MG_MOUSE
APOA2_MOUSE	FETUB_MOUSE	G3P_MOUSE
APOA4_MOUSE	GCAM_MOUSE	GCAB_MOUSE
APOC1_MOUSE	HPT_MOUSE	HBB1_MOUSE
APOC2_MOUSE	HVM00_MOUSE	HVM03_MOUSE
APOC3_MOUSE	HVM09_MOUSE	HVM10_MOUSE
APOE_MOUSE	HVM17_MOUSE	HVM14_MOUSE
APOH_MOUSE	HVM21_MOUSE	HVM28_MOUSE
C1QA_MOUSE	HVM27_MOUSE	HVM52_MOUSE
C1QB_MOUSE	HVM44_MOUSE	K1C10_MOUSE
C1QC_MOUSE	HVM45_MOUSE	K22E_MOUSE
C1RA_MOUSE	HVM54_MOUSE	K2C1_MOUSE
C4BPA_MOUSE	HVM56_MOUSE	KV3A1_MOUSE
CD5L_MOUSE	IGHA_MOUSE	KV3AC_MOUSE

CFAH_MOUSE
CLUS_MOUSE
CO3_MOUSE
CO4B_MOUSE
CRP_MOUSE
CS1A_MOUSE
FETUA_MOUSE
FIBA_MOUSE
FIBB_MOUSE
FIBG_MOUSE
FINC_MOUSE
GELS_MOUSE
HA10_MOUSE
HBA_MOUSE
HEMO_MOUSE
HRG_MOUSE
HVM05_MOUSE
HVM06_MOUSE
HVM16_MOUSE
HVM36_MOUSE
HVM51_MOUSE
HVM57_MOUSE
HVM60_MOUSE
IC1_MOUSE
IGG2B_MOUSE
IGHG3_MOUSE
IGHM_MOUSE
IGJ_MOUSE
IGKC_MOUSE
ITIH4_MOUSE
K2C6A_MOUSE
KNG1_MOUSE
KV2A6_MOUSE
KV2A7_MOUSE
KV3A4_MOUSE
KV3A8_MOUSE
KV3AI_MOUSE
KV3AM_MOUSE
KV4A1_MOUSE
KV5A1_MOUSE
KV5A3_MOUSE
IGHG1_MOUSE
KV3AG_MOUSE
KV3AJ_MOUSE
KV6A5_MOUSE
KVM5_MOUSE
MUG1_MOUSE
NPY_MOUSE
PLF4_MOUSE
SAA1_MOUSE
SAMP_MOUSE
VTNC_MOUSE
KV3AE_MOUSE
LBP_MOUSE
LV1C_MOUSE
PLTP_MOUSE
PRDX2_MOUSE
SPTA1_MOUSE

KV5A4_MOUSE
KV5A6_MOUSE
KV5A7_MOUSE
KV5A9_MOUSE
KV5AB_MOUSE
KV6A7_MOUSE
KV6AB_MOUSE
LAC1_MOUSE
LOXL1_MOUSE
MASP1_MOUSE
MASP2_MOUSE
MBL1_MOUSE
MBL2_MOUSE
PLMN_MOUSE
PROP_MOUSE
PZP_MOUSE
SPA3K_MOUSE
THRB_MOUSE
TRFE_MOUSE
TSP1_MOUSE
TTHY_MOUSE
VTDB_MOUSE

4h Ball Milled SWCNTs

**Shared**	**Normal Serum Only**	**Hyperlipidemic Serum Only**
A1AT3_MOUSE	ACTG_MOUSE	A1AT2_MOUSE
A1AT4_MOUSE	ANT3_MOUSE	APOB_MOUSE
ALBU_MOUSE	APOH_MOUSE	APOC4_MOUSE
APOA1_MOUSE	BPIA2_MOUSE	B2MG_MOUSE
APOA2_MOUSE	CFAB_MOUSE	GCAB_MOUSE
APOA4_MOUSE	ECM1_MOUSE	HVM03_MOUSE
APOC1_MOUSE	EST1C_MOUSE	HVM14_MOUSE
APOC2_MOUSE	FA5_MOUSE	HVM35_MOUSE
APOC3_MOUSE	FETUB_MOUSE	HVM52_MOUSE
APOE_MOUSE	GCAA_MOUSE	K1C15_MOUSE
C1QA_MOUSE	HPT_MOUSE	K2C6A_MOUSE
C1QB_MOUSE	HVM00_MOUSE	KRA65_MOUSE
C1QC_MOUSE	HVM21_MOUSE	KRT86_MOUSE
C1RA_MOUSE	HVM32_MOUSE	KT33A_MOUSE
C4BPA_MOUSE	HVM44_MOUSE	KV3A3_MOUSE

CD5L_MOUSE
CFAH_MOUSE
CLUS_MOUSE
CO3_MOUSE
CO4B_MOUSE
CRP_MOUSE
CS1A_MOUSE
FETUA_MOUSE
FIBA_MOUSE
FIBB_MOUSE
FIBG_MOUSE
FINC_MOUSE
GELS_MOUSE
HA10_MOUSE
HBA_MOUSE
HBB1_MOUSE
HEMO_MOUSE
HRG_MOUSE
HVM05_MOUSE
HVM06_MOUSE
HVM09_MOUSE
HVM16_MOUSE
HVM17_MOUSE
HVM36_MOUSE
HVM51_MOUSE
HVM57_MOUSE
HVM60_MOUSE
IC1_MOUSE
IGG2B_MOUSE
IGHG3_MOUSE
IGHM_MOUSE
IGJ_MOUSE
IGKC_MOUSE
ITIH4_MOUSE
K1C10_MOUSE
K22E_MOUSE
K2C74_MOUSE
KV2A6_MOUSE
KV2A7_MOUSE
KV3A8_MOUSE
KV3AI_MOUSE
HVM45_MOUSE
HVM53_MOUSE
HVM54_MOUSE
HVM56_MOUSE
IGHG1_MOUSE
K1C13_MOUSE
KNG1_MOUSE
KV2A5_MOUSE
KV3A4_MOUSE
KV3A5_MOUSE
KV3AB_MOUSE
KV3AE_MOUSE
KV3AJ_MOUSE
KV5AA_MOUSE
KV5AC_MOUSE
KV5AF_MOUSE
KV6A5_MOUSE
KV6AA_MOUSE
LAC1_MOUSE
LAC2_MOUSE
LV1A_MOUSE
MUG1_MOUSE
NPY_MOUSE
PLMN_MOUSE
SAA1_MOUSE
SAA2_MOUSE
SAMP_MOUSE
VTNC_MOUSE
KV3AC_MOUSE
KV4A1_MOUSE
KV5AB_MOUSE
LBP_MOUSE
LV1B_MOUSE
TETN_MOUSE
ZEP2_MOUSE

KV3AM_MOUSE
KV5A1_MOUSE
KV5A3_MOUSE
KV5A4_MOUSE
KV5A6_MOUSE
KV5A7_MOUSE
KV5A9_MOUSE
KV6AB_MOUSE
LOXL1_MOUSE
MASP1_MOUSE
MASP2_MOUSE
MBL1_MOUSE
MBL2_MOUSE
PLF4_MOUSE
PROP_MOUSE
PZP_MOUSE
SPA3K_MOUSE
THRB_MOUSE
TRFE_MOUSE
TSP1_MOUSE
TTHY_MOUSE
VTDB_MOUSE

6h Ball Milled SWCNTs

Shared	Normal Serum Only	Hyperlipidemic Serum Only
A1AT3_MOUSE	ANT3_MOUSE	A1AT2_MOUSE
ACTB_MOUSE	CFAB_MOUSE	A1AT4_MOUSE
ALBU_MOUSE	GCAA_MOUSE	APOB_MOUSE
APOA1_MOUSE	HVM00_MOUSE	B2MG_MOUSE
APOA2_MOUSE	HVM17_MOUSE	CO5_MOUSE
APOA4_MOUSE	HVM18_MOUSE	GCAB_MOUSE
APOC1_MOUSE	HVM45_MOUSE	GPX3_MOUSE
APOC2_MOUSE	HVM54_MOUSE	HBA_MOUSE
APOC3_MOUSE	HVM56_MOUSE	HVM03_MOUSE
APOE_MOUSE	IGHA_MOUSE	HVM14_MOUSE
APOH_MOUSE	IGHG1_MOUSE	HVM52_MOUSE
C1QA_MOUSE	K22E_MOUSE	K1C10_MOUSE
C1QB_MOUSE	K2C74_MOUSE	K1C13_MOUSE
C1QC_MOUSE	KNG1_MOUSE	K1C14_MOUSE
C1RA_MOUSE	KV3A8_MOUSE	K2C1_MOUSE

C4BPA_MOUSE
CD5L_MOUSE
CFAH_MOUSE
CLUS_MOUSE
CO3_MOUSE
CO4B_MOUSE
CRP_MOUSE
CS1A_MOUSE
FA5_MOUSE
FETUA_MOUSE
FIBA_MOUSE
FIBB_MOUSE
FIBG_MOUSE
FINC_MOUSE
GELS_MOUSE
HA10_MOUSE
HBB1_MOUSE
HEMO_MOUSE
HRG_MOUSE
HVM05_MOUSE
HVM06_MOUSE
HVM09_MOUSE
HVM16_MOUSE
HVM35_MOUSE
HVM36_MOUSE
HVM51_MOUSE
HVM57_MOUSE
HVM60_MOUSE
IC1_MOUSE
IGG2B_MOUSE
IGHG3_MOUSE
IGHM_MOUSE
IGJ_MOUSE
IGKC_MOUSE
ITIH4_MOUSE
KV2A6_MOUSE
KV2A7_MOUSE
KV3A4_MOUSE
KV3AM_MOUSE
KV4A1_MOUSE
KV5A1_MOUSE
KV3AB_MOUSE
KV3AG_MOUSE
KV3AI_MOUSE
KV3AJ_MOUSE
KV5AB_MOUSE
KV6A5_MOUSE
KV6A6_MOUSE
KVM5_MOUSE
PLF4_MOUSE
PLMN_MOUSE
SAA1_MOUSE
SAMP_MOUSE
SPTB1_MOUSE
VTNC_MOUSE
K2C1B_MOUSE
K2C6A_MOUSE
K2C79_MOUSE
KRT35_MOUSE
KV3A7_MOUSE
KV3AC_MOUSE
KV5AC_MOUSE
KV6A7_MOUSE
LAC1_MOUSE
LAC2_MOUSE
LBP_MOUSE
LV1B_MOUSE
PLTP_MOUSE
QSOX1_MOUSE

KV5A3_MOUSE
KV5A4_MOUSE
KV5A6_MOUSE
KV5A7_MOUSE
KV5A9_MOUSE
KV6AB_MOUSE
LOXL1_MOUSE
MASP1_MOUSE
MASP2_MOUSE
MBL1_MOUSE
MBL2_MOUSE
MUG1_MOUSE
PROP_MOUSE
PZP_MOUSE
SPA3K_MOUSE
THRB_MOUSE
TRFE_MOUSE
TSP1_MOUSE
TTHY_MOUSE
VTDB_MOUSE

8h Ball Milled SWCNTs

Shared	Normal Serum Only	Hyperlipidemic Serum Only
A1AT2_MOUSE	A1AG1_MOUSE	A1AT4_MOUSE
A1AT3_MOUSE	AMBP_MOUSE	A1AT5_MOUSE
ACTB_MOUSE	APOH_MOUSE	APOB_MOUSE
ALBU_MOUSE	C4BPA_MOUSE	B2MG_MOUSE
APOA1_MOUSE	CBPB2_MOUSE	HVM06_MOUSE
APOA2_MOUSE	CFAB_MOUSE	HVM09_MOUSE
APOA4_MOUSE	CFAD_MOUSE	HVM52_MOUSE
APOC1_MOUSE	CFAI_MOUSE	IC1_MOUSE
APOC2_MOUSE	CLC11_MOUSE	K2C74_MOUSE
APOC3_MOUSE	CO5_MOUSE	KV2A6_MOUSE
APOE_MOUSE	CO8A_MOUSE	KV3AC_MOUSE
C1QA_MOUSE	CO8B_MOUSE	KV5A9_MOUSE
C1QB_MOUSE	CO8G_MOUSE	LBP_MOUSE
C1QC_MOUSE	CO9_MOUSE	LOXL1_MOUSE
C1RA_MOUSE	ECM1_MOUSE	LV1C_MOUSE
CD5L_MOUSE	EST1C_MOUSE	PLTP_MOUSE
CFAH_MOUSE	FA12_MOUSE	PRDX2_MOUSE

CLUS_MOUSE
CO3_MOUSE
CO4B_MOUSE
CRP_MOUSE
CS1A_MOUSE
FETUA_MOUSE
FIBA_MOUSE
FIBB_MOUSE
FIBG_MOUSE
FINC_MOUSE
GELS_MOUSE
HA10_MOUSE
HBA_MOUSE
HEMO_MOUSE
HRG_MOUSE
HVM16_MOUSE
HVM35_MOUSE
HVM51_MOUSE
HVM57_MOUSE
HVM60_MOUSE
IGHG3_MOUSE
IGHM_MOUSE
IGJ_MOUSE
IGKC_MOUSE
ITIH4_MOUSE
K1C10_MOUSE
KNG1_MOUSE
KV2A7_MOUSE
KV3A4_MOUSE
KV3A8_MOUSE
KV3AB_MOUSE
KV3AM_MOUSE
KV4A1_MOUSE
KV5A1_MOUSE
KV5A3_MOUSE
KV5A4_MOUSE
KV5A6_MOUSE
KV5A7_MOUSE
KV5AB_MOUSE
KV6AB_MOUSE
MASP1_MOUSE

FA5_MOUSE
GCAA_MOUSE
GP1BA_MOUSE
HBB1_MOUSE
HVM00_MOUSE
HVM17_MOUSE
HVM21_MOUSE
HVM36_MOUSE
HVM45_MOUSE
HVM54_MOUSE
HVM56_MOUSE
HVM63_MOUSE
IGG2B_MOUSE
IGHA_MOUSE
IGHG1_MOUSE
ITIH3_MOUSE
K1C15_MOUSE
K22E_MOUSE
K2C6A_MOUSE
K2C73_MOUSE
K2C79_MOUSE
KV3AG_MOUSE
KV3AJ_MOUSE
KV6A5_MOUSE
LAC1_MOUSE
LAC2_MOUSE
LV1B_MOUSE
MUG1_MOUSE
PEDF_MOUSE
PLF4_MOUSE
PLMN_MOUSE
SEPP1_MOUSE
TIMP3_MOUSE
VTNC_MOUSE

TTHY_MOUSE

MASP2_MOUSE
MBL1_MOUSE
MBL2_MOUSE
PROP_MOUSE
PZP_MOUSE
SPA3K_MOUSE
THRB_MOUSE
TRFE_MOUSE
TSP1_MOUSE
VTDB_MOUSE

Supplemental Data Table 8. Relative Quantification of Proteins Associated with SWCNTs Following Incubation in either Normal or Hyperlipidemic Serum

All values are ratios of Normal Serum : Hyperlipidemic Serum

Accession	Protein	As Prepared	CV %	2h Ball Milled	CV %	4h Ball Milled	CV %	6h Ball Milled	CV %	8h Ball Milled	CV %
A1AT2_MOUSE	Alpha-1-antitrypsin 1-2 OS=Mus musculus GN=Serpina1b PE=1 SV=2	4.348	0			4.167	0	3.333	16.53	3.571	1.71
A1AT3_MOUSE	Alpha-1-antitrypsin 1-3 OS=Mus musculus GN=Serpina1c PE=1 SV=2	4.000	0			4.167	0	4.000	23.64	5.263	45.04
A1AT4_MOUSE	Alpha-1-antitrypsin 1-4 OS=Mus musculus GN=Serpina1d PE=1 SV=1					4.167	0	3.846	0	3.448	0
A1AT5_MOUSE	Alpha-1-antitrypsin 1-5 OS=Mus musculus GN=Serpina1e PE=1 SV=1									3.448	0
ACTBL_MOUSE	Beta-actin-like protein 2 OS=Mus musculus GN=Actbl2 PE=1 SV=1			0.658	0						
ALBU_MOUSE	Serum albumin OS=Mus musculus GN=Alb PE=1 SV=3	1.818	21.31			3.125	27.35	2.326	22	3.333	32.07
APC2_MOUSE	Adenomatous polyposis coli protein 2 OS=Mus musculus GN=Apc2 PE=1 SV=1	1.639	0								
APOA1_MOUSE	Apolipoprotein A-I OS=Mus musculus GN=Apoa1 PE=1 SV=2	0.595	0			5.000	0	1.961	39.44	3.125	63.61
APOA2_MOUSE	Apolipoprotein A-II OS=Mus musculus GN=Apoa2 PE=1 SV=2	0.298	0	0.361	0					0.305	0
APOA4_MOUSE	Apolipoprotein A-IV OS=Mus musculus GN=Apoa4 PE=1 SV=3			0.602	8.38					1.587	7.09
APOC1_MOUSE	Apolipoprotein C-I OS=Mus musculus GN=Apoc1 PE=1 SV=1	0.137	0							0.100	0
APOC2_MOUSE	Apolipoprotein C-II OS=Mus musculus GN=Apoc2 PE=2 SV=1							2.857	0	1.471	123.22
APOC3_MOUSE	Apolipoprotein C-III OS=Mus musculus GN=Apoc3 PE=1 SV=2									3.448	0
APOE_MOUSE	Apolipoprotein E OS=Mus musculus GN=Apoe PE=1 SV=2	0.108	22.06	0.149	23.62					0.592	8.92
APOH_MOUSE	Beta-2-glycoprotein 1 OS=Mus musculus GN=Apoh PE=1 SV=1	3.226	0					1.639	0		
C1QA_MOUSE	Complement C1q subcomponent subunit A OS=Mus musculus GN=C1qa PE=1 SV=2					4.762	27.06				
C1QB_MOUSE	Complement C1q subcomponent subunit B OS=Mus musculus GN=C1qb PE=1 SV=2	0.326	15.91							2.632	0
C1QC_MOUSE	Complement C1q subcomponent subunit C OS=Mus musculus GN=C1qc PE=1 SV=2					3.704	8.36	2.941	6.56		
C1RA_MOUSE	Complement C1r-A subcomponent OS=Mus musculus GN=C1ra PE=1 SV=1	0.694	0	3.030	0	3.846	4.97	2.857	8.19	3.333	7.85
C4BPA_MOUSE	C4b-binding protein OS=Mus musculus GN=C4bpa PE=1 SV=3					3.333	0			25.000	36.25
CD5L_MOUSE	CD5 antigen-like OS=Mus musculus GN=Cd5l PE=1 SV=3									1.818	1.85
CFAH_MOUSE	Complement factor H OS=Mus musculus GN=Cfh PE=1 SV=2			2.500	11.66	2.564	0	1.786	0	20.000	45.8
CL056_MOUSE	Uncharacterized protein C12orf56 homolog OS=Mus musculus PE=2 SV=1	3.333	0								
CLUS_MOUSE	Clusterin OS=Mus musculus GN=Clu PE=1 SV=1			0.481	0					2.041	22.8
CO3_MOUSE	Complement C3 OS=Mus musculus GN=C3 PE=1 SV=3	0.645	71			3.226	23.93	2.041	95.13	14.286	51.75
CO4B_MOUSE	Complement C4-B OS=Mus musculus GN=C4b PE=1 SV=3	1.887	129.12	3.846	36.74	7.692	20.5	3.846	47.07	7.143	198.37
CRP_MOUSE	C-reactive protein OS=Mus musculus GN=Crp PE=1 SV=2			0.599	0	3.030	25.68				
CS1A_MOUSE	Complement C1s-A subcomponent OS=Mus musculus GN=C1sa PE=2 SV=2	0.581	0					2.273	12.01	3.571	31.93
DI3L2_MOUSE	DIS3-like exonuclease 2 OS=Mus musculus GN=Dis3l2 PE=1 SV=1							2.941	0		
FA5_MOUSE	Coagulation factor V OS=Mus musculus GN=F5 PE=1 SV=1	1.266	0	3.571	21.05			5.000	0	5.263	0
FETUA_MOUSE	Alpha-2-HS-glycoprotein OS=Mus musculus GN=Ahsg PE=1 SV=1	4.000	0	1.075	129.21			2.326	70.82	3.333	4.14
FIBA_MOUSE	Fibrinogen alpha chain OS=Mus musculus GN=Fga PE=1 SV=1							1.449	0		
FIBG_MOUSE	Fibrinogen gamma chain OS=Mus musculus GN=Fgg PE=1 SV=1			0.337	0			0.318	0	1.563	0
FINC_MOUSE	Fibronectin OS=Mus musculus GN=Fn1 PE=1 SV=4							0.758	0	5.000	31.2
GELS_MOUSE	Gelsolin OS=Mus musculus GN=Gsn PE=1 SV=3			2.222	24.28	2.500	0	1.923	37.44	5.263	37.65
GUF1_MOUSE	Translation factor Guf1, mitochondrial OS=Mus musculus GN=Guf1 PE=1 SV=1									0.588	0
HA10_MOUSE	H-2 class I histocompatibility antigen, Q10 alpha chain OS=Mus musculus GN=H2-Q10 PE=1 SV=3	2.041	19.39					1.220	0	1.471	0
HBA_MOUSE	Hemoglobin subunit alpha OS=Mus musculus GN=Hba PE=1 SV=2							2.041	0	2.222	0
HBB1_MOUSE	Hemoglobin subunit beta-1 OS=Mus musculus GN=Hbb-b1 PE=1 SV=2	2.381	0					1.449	0		
HEMO_MOUSE	Hemopexin OS=Mus musculus GN=Hpx PE=1 SV=2							1.563	10.32		
HRG_MOUSE	Histidine-rich glycoprotein OS=Mus musculus GN=Hrg PE=1 SV=2	0.214	0					2.000	0	12.500	0
HVM00_MOUSE	Ig heavy chain V region OS=Mus musculus PE=1 SV=1							2.564	0	5.000	0
HVM09_MOUSE	Ig heavy chain V region 186-1 OS=Mus musculus PE=4 SV=2									0.316	0
HVM17_MOUSE	Ig heavy chain V region MOPC 47A OS=Mus musculus PE=1 SV=1					7.692	0	3.125	0		
HVM21_MOUSE	Ig heavy chain V region M511 OS=Mus musculus PE=1 SV=1			12.500	0	33.333	0				
HVM28_MOUSE	Ig heavy chain V-III region U61 OS=Mus musculus PE=1 SV=1			9.091	0						
HVM32_MOUSE	Ig heavy chain V-III region J606 OS=Mus musculus PE=1 SV=1					20.000	0				
HVM35_MOUSE	Ig heavy chain V-III region HPC76 (Fragment) OS=Mus musculus PE=4 SV=1									11.111	0

HVM36_MOUSE	Ig heavy chain V region 441 OS=Mus musculus PE=4 SV=1			2.941	0			3.704	0		
HVM51_MOUSE	Ig heavy chain V region AC38 205.12 OS=Mus musculus PE=1 SV=1	0.123	0							0.322	0
HVM54_MOUSE	Ig heavy chain V region 5-84 OS=Mus musculus PE=1 SV=1	3.846	0	2.564	0					5.882	0
HVM60_MOUSE	Ig heavy chain V region 3-6 OS=Mus musculus GN=Ighv3-6 PE=1 SV=1	0.538	0								
IC1_MOUSE	Plasma protease C1 inhibitor OS=Mus musculus GN=Serping1 PE=1 SV=3	2.857	0								
IGHG1_MOUSE	Ig gamma-1 chain C region secreted form OS=Mus musculus GN=Ighg1 PE=1 SV=1	5.263	0								
IGHG3_MOUSE	Ig gamma-3 chain C region OS=Mus musculus PE=1 SV=2	2.941	4.23	3.030	22.49	10.000	30.94	5.000	28.17	16.667	26.1
IGHM_MOUSE	Ig mu chain C region OS=Mus musculus GN=Ighm PE=1 SV=2	0.592	16.49	3.226	193.51	4.167	68.2	2.703	39.53	4.000	114.66
IGJ_MOUSE	Immunoglobulin J chain OS=Mus musculus GN=Jchain PE=1 SV=4					2.778	19.55			1.754	10.27
IGKC_MOUSE	Ig kappa chain C region OS=Mus musculus PE=1 SV=1					5.000	1.26	2.222	0	2.439	33.46
ITIH4_MOUSE	Inter alpha-trypsin inhibitor, heavy chain 4 OS=Mus musculus GN=Itih4 PE=1 SV=2	2.941	0	1.923	0	3.448	32.61	2.381	15.62	3.704	0
KV2A6_MOUSE	Ig kappa chain V-II region 7S34.1 OS=Mus musculus PE=1 SV=1			2.778	0	11.111	50.4	2.632	0		
KV2A7_MOUSE	Ig kappa chain V-II region 26-10 OS=Mus musculus PE=1 SV=1			5.000	20.91			3.704	36.57	7.692	41.3
KV3A1_MOUSE	Ig kappa chain V-III region PC 2880/PC 1229 OS=Mus musculus PE=1 SV=1	0.478	0.03								
KV3A4_MOUSE	Ig kappa chain V-III region 50S10.1 OS=Mus musculus PE=1 SV=1	0.478	0.03			3.125	0				
KV3A5_MOUSE	Ig kappa chain V-III region PC 2413 OS=Mus musculus PE=1 SV=1					3.125	0				
KV3A8_MOUSE	Ig kappa chain V-III region PC 3741/TEPC 111 OS=Mus musculus PE=1 SV=1	0.348	49.28			3.226	1.28				
KV3AA_MOUSE	Ig kappa chain V-III region ABPC 22/PC 9245 OS=Mus musculus PE=1 SV=1							4.348	70.44		
KV3AB_MOUSE	Ig kappa chain V-III region PC 4050 OS=Mus musculus PE=1 SV=1	0.478	0			5.000	64.99				
KV3AC_MOUSE	Ig kappa chain V-III region CBPC 101 OS=Mus musculus PE=1 SV=1	0.478	0								
KV3AE_MOUSE	Ig kappa chain V-III region PC 7183 OS=Mus musculus PE=1 SV=1					3.125	0				
KV3AI_MOUSE	Ig kappa chain V-III region PC 6684 OS=Mus musculus PE=1 SV=1	0.478	0	6.667	0	9.091	87.01	8.333	5.74		
KV3AJ_MOUSE	Ig kappa chain V-III region PC 7175 OS=Mus musculus PE=1 SV=1	0.478	0	6.667	0	9.091	87.01	8.333	5.74		
KV3AM_MOUSE	Ig kappa chain V-III region PC 2154 OS=Mus musculus PE=1 SV=1							0.610	0		
KV4A1_MOUSE	Ig kappa chain V-IV region S107B OS=Mus musculus PE=4 SV=1									4.167	0
KV5A1_MOUSE	Ig kappa chain V19-17 OS=Mus musculus GN=Igk-V19-17 PE=1 SV=1	0.485	0					4.762	0	11.111	0
KV5A3_MOUSE	Ig kappa chain V-V region K2 (Fragment) OS=Mus musculus PE=1 SV=1			1.538	0			2.381	0	2.941	0
KV5A3_MOUSE	Ig kappa chain V-V region K2 (Fragment) OS=Mus musculus PE=1 SV=1										
KV5A6_MOUSE	Ig kappa chain V-V region L6 (Fragment) OS=Mus musculus PE=4 SV=1	0.426	0	1.389	120.32	11.111	0	5.882	0	5.882	0
KV5A7_MOUSE	Ig kappa chain V-V region MOPC 41 OS=Mus musculus GN=Gm5571 PE=1 SV=1			2.857	0						
KV5A7_MOUSE	Ig kappa chain V-V region MOPC 41 OS=Mus musculus GN=Gm5571 PE=1 SV=1					6.667	0	2.632	8.26	2.632	0.6
KV5AB_MOUSE	Ig kappa chain V-V region HP R16.7 OS=Mus musculus PE=1 SV=1					3.333	0				
KV5AF_MOUSE	Ig kappa chain V-V region HP 91A3 OS=Mus musculus PE=1 SV=1					3.333	0				
KV5AG_MOUSE	Ig kappa chain V-V regions OS=Mus musculus PE=1 SV=1	0.493	0								
KV6A5_MOUSE	Ig kappa chain V-VI region J539 OS=Mus musculus PE=1 SV=1			5.263	0						
KV6A7_MOUSE	Ig kappa chain V-VI region NQ2-48.2.2 OS=Mus musculus PE=2 SV=1									4.000	0
KV6AB_MOUSE	Ig kappa chain V-VI region NQ2-6.1 OS=Mus musculus PE=2 SV=1									0.290	0
LAC1_MOUSE	Ig lambda-1 chain C region OS=Mus musculus PE=1 SV=1			0.201	0						
LOXL1_MOUSE	Lysyl oxidase homolog 1 OS=Mus musculus GN=Loxl1 PE=2 SV=3	0.543	0								
LV1B_MOUSE	Ig lambda-1 chain V regions MOPC 104E/RPC20/J558/S104 OS=Mus musculus PE=1 SV=1									4.000	0
MASP2_MOUSE	Mannan-binding lectin serine protease 2 OS=Mus musculus GN=Masp2 PE=1 SV=1	0.481	0.05			2.326	0			5.000	0
MBL1_MOUSE	Mannose-binding protein A OS=Mus musculus GN=Mbl1 PE=1 SV=1			2.381	0	4.167	0	2.439	0	12.500	0
MBL2_MOUSE	Mannose-binding protein C OS=Mus musculus GN=Mbl2 PE=1 SV=2	0.455	0	2.439	0	3.846	1.7	2.500	28.2	3.571	32.49
MUP1_MOUSE	Major urinary protein 1 OS=Mus musculus GN=Mup1 PE=1 SV=1	3.333	30.28								
MUP2_MOUSE	Major urinary protein 2 OS=Mus musculus GN=Mup2 PE=1 SV=1	2.941	23.91								
PLF4_MOUSE	Platelet factor 4 OS=Mus musculus GN=Pf4 PE=1 SV=1							2.857	0	3.226	0
PLMN_MOUSE	Plasminogen OS=Mus musculus GN=Plg PE=1 SV=3	0.485	0	1.961	16.72			1.923	0		
PROP_MOUSE	Properdin OS=Mus musculus GN=Cfp PE=2 SV=2									9.091	31.22
PZP_MOUSE	Pregnancy zone protein OS=Mus musculus GN=Pzp PE=1 SV=3			0.667	0						
SBSN_MOUSE	Suprabasin OS=Mus musculus GN=Sbsn PE=2 SV=1	0.450	0								
SPA3K_MOUSE	Serine protease inhibitor A3K OS=Mus musculus GN=Serpina3k PE=1 SV=2							2.439	15.47	2.857	10.37
SSBP_MOUSE	Single-stranded DNA-binding protein, mitochondrial OS=Mus musculus GN=Ssbp1 PE=1 SV=1									7.143	0

TETN_MOUSE	Tetranectin OS=Mus musculus GN=Clec3b PE=1 SV=2	2.083	0								
THRB_MOUSE	Prothrombin OS=Mus musculus GN=F2 PE=1 SV=1			1.667	0	2.381	0	1.515	13.07		
TRFE_MOUSE	Serotransferrin OS=Mus musculus GN=Tf PE=1 SV=1	2.326	17.96		26.12	4.000	0	1.887	19.52	2.000	0
TSP1_MOUSE	Thrombospondin-1 OS=Mus musculus GN=Thbs1 PE=1 SV=1			4.762				5.556	13.86	16.667	22.2
VTDB_MOUSE	Vitamin D-binding protein OS=Mus musculus GN=Gc PE=1 SV=2							1.852	0	2.632	0
VTNC_MOUSE	Vitronectin OS=Mus musculus GN=Vtn PE=1 SV=2			3.226	0	14.286	9.29				

Supplemental Data Table 9. Differences in pathways and protein characteristics due to defects following incubation in either normal or hyperlipidemic serum.

All proteins that associated with the As Prepared SWCNTs were uploaded into geneontology.org's enrichment analysis tool and search for biological processes and Mus musculus. Analysis utilized the Bonferroni correction for multiple testing with a statistical significance established at $p<0.05$. The table presents all pathways that were identified regardless of signifiicance in order to demonstrate differences between groups. All mapped IDs were utilized for PANTHER analysis of pathways and protein characteristics. Separately, all proteins that were found to associate following introduction of defects by ball milling were uploaded into geneontology's enrichment analysis tool and the same assessment was performed. This analysis was performed individually for both serum types (normal and hyperlipidemic). Assessment of PANTHER pathways allows for identification of distinct biological pathways that may be disrupted due to introduction of SWCNTs whereas assessment of protein characteristics allows for the understanding of protein properties that may result in association with SWCNTs.

Biological pathways related to the BC that formed following incubation in normal serum

As Prepared

PANTHER Pathways	#	Fold Enrichment	P-value
Blood coagulation (P00011)	11	53.49	4.93E-14
Plasminogen activating cascade (P00050)	3	43.77	7.80E-03
Vitamin D metabolism and pathway (P04396)	2	38.16	2.07E-01
Integrin signalling pathway (P00034)	2	2.6	1.00E+00
Huntington disease (P00029)	2	3.42	1.00E+00
CCKR signaling map (P06959)	1	1.5	1.00E+00
p53 pathway (P00059)	1	2.95	1.00E+00
Wnt signaling pathway (P00057)	1	0.82	1.00E+00
T cell activation (P00053)	1	2.7	1.00E+00
Nicotinic acetylcholine receptor signaling pathway (P00044)	1	2.61	1.00E+00
Inflammation mediated by chemokine and cytokine signaling pathway (P00031)	1	0.95	1.00E+00
Glycolysis (P00024)	1	8.86	1.00E+00
FAS signaling pathway (P00020)	1	7.75	1.00E+00

Cytoskeletal regulation by Rho GTPase (P00016)	1	3.14	1.00E+00
Cadherin signaling pathway (P00012)	1	1.61	1.00E+00
B cell activation (P00010)	1	3.49	1.00E+00
Alzheimer disease-presenilin pathway (P00004)	1	2.02	1.00E+00

Ball Milled

PANTHER Pathways	#	Fold Enrichment	P-Value
Blood coagulation (P00011)	13	52.2	1.39E-16
Plasminogen activating cascade (P00050)	5	60.23	4.43E-06
Integrin signalling pathway (P00034)	3	3.22	1.00E+00
Inflammation mediated by chemokine and cytokine signaling pathway (P00031)	3	2.36	1.00E+00
Wnt signaling pathway (P00057)	2	1.35	1.00E+00
Nicotinic acetylcholine receptor signaling pathway (P00044)	2	4.31	1.00E+00
Huntington disease (P00029)	2	2.82	1.00E+00
Cytoskeletal regulation by Rho GTPase (P00016)	2	5.18	1.00E+00
Cadherin signaling pathway (P00012)	2	2.66	1.00E+00
Alzheimer disease-presenilin pathway (P00004)	2	3.33	1.00E+00
CCKR signaling map (P06959)	1	1.24	1.00E+00
p53 pathway (P00059)	1	2.44	1.00E+00
Vitamin D metabolism and pathway (P04396)	1	15.75	1.00E+00
FAS signaling pathway (P00020)	1	6.4	1.00E+00

Characteristics of proteins found to associate with the BC that formed following incubation in normal serum

As Prepared

Category Name (Accession)	# genes	% genes hit against total genes	% gene hit against total # protein class hits
enzyme modulator (PC00095)	17	18.90%	14.70%
hydrolase (PC00121)	16	17.80%	13.80%
transfer/carrier protein (PC00219)	14	15.60%	12.10%

defense/immunity protein (PC00090)	14	15.60%	12.10%
signaling molecule (PC00207)	14	15.60%	12.10%
receptor (PC00197)	9	10.00%	7.80%
transporter (PC00227)	7	7.80%	6.00%
oxidoreductase (PC00176)	6	6.70%	5.20%
extracellular matrix protein (PC00102)	6	6.70%	5.20%
calcium-binding protein (PC00060)	5	5.60%	4.30%
cell adhesion molecule (PC00069)	4	4.40%	3.40%
isomerase (PC00135)	2	2.20%	1.70%
cytoskeletal protein (PC00085)	2	2.20%	1.70%

Ball Milled

Category Name (Accession)	# genes	% genes hit against total genes	% gene hit against total # protein class hits
enzyme modulator (PC00095)	22	20.20%	15.80%
signaling molecule (PC00207)	18	16.50%	12.90%
hydrolase (PC00121)	17	15.60%	12.20%
defense/immunity protein (PC00090)	16	14.70%	11.50%
transfer/carrier protein (PC00219)	13	11.90%	9.40%
cytoskeletal protein (PC00085)	12	11.00%	8.60%
receptor (PC00197)	9	8.30%	6.50%
structural protein (PC00211)	8	7.30%	5.80%
transporter (PC00227)	7	6.40%	5.00%
extracellular matrix protein (PC00102)	6	5.50%	4.30%
calcium-binding protein (PC00060)	5	4.60%	3.60%
oxidoreductase (PC00176)	3	2.80%	2.20%
cell adhesion molecule (PC00069)	3	2.80%	2.20%

Biological pathways related to the BC that formed following incubation in hyperlipidemic serum

As Prepared

PANTHER Pathways	#	Fold Enrichment	P-value
Blood coagulation (P00011)	10	54.71	8.72E-13
Plasminogen activating cascade (P00050)	3	49.24	5.49E-03
Vitamin D metabolism and pathway (P04396)	2	42.93	1.64E-01
CCKR signaling map (P06959)	1	1.69	1.00E+00
p53 pathway (P00059)	1	3.32	1.00E+00
T cell activation (P00053)	1	3.03	1.00E+00
Integrin signalling pathway (P00034)	1	1.46	1.00E+00
FAS signaling pathway (P00020)	1	8.72	1.00E+00
B cell activation (P00010)	1	3.93	1.00E+00

Ball Milled

PANTHER Pathways	#	Fold Enrichment	P-value
Blood coagulation (P00011)	12	52	3.16E-15
Plasminogen activating cascade (P00050)	5	65	3.02E-06
Integrin signalling pathway (P00034)	3	3.47	1.00E+00
Inflammation mediated by chemokine and cytokine signaling pathway (P00031)	3	2.55	1.00E+00
Huntington disease (P00029)	3	4.57	1.00E+00
Nicotinic acetylcholine receptor signaling pathway (P00044)	2	4.65	1.00E+00
Cytoskeletal regulation by Rho GTPase (P00016)	2	5.6	1.00E+00
Cadherin signaling pathway (P00012)	2	2.87	1.00E+00
Alzheimer disease-presenilin pathway (P00004)	2	3.59	1.00E+00
CCKR signaling map (P06959)	1	1.34	1.00E+00
p53 pathway (P00059)	1	2.63	1.00E+00
Wnt signaling pathway (P00057)	1	0.73	1.00E+00
T cell activation (P00053)	1	2.4	1.00E+00
Vitamin D metabolism and pathway (P04396)	1	17	1.00E+00

Glycolysis (P00024)	1	7.89	1.00E+00
FAS signaling pathway (P00020)	1	6.91	1.00E+00
B cell activation (P00010)	1	3.11	1.00E+00

Characteristics of proteins found to associate with the BC that formed following incubation in hyperlipidemic serum

As Prepared

Category Name (Accession)	# genes	% genes hit against total genes	% gene hit against total # protein class hits
enzyme modulator (PC00095)	15	18.80%	14.90%
hydrolase (PC00121)	14	17.50%	13.90%
defense/immunity protein (PC00090)	14	17.50%	13.90%
transfer/carrier protein (PC00219)	13	16.30%	12.90%
signaling molecule (PC00207)	11	13.80%	10.90%
transporter (PC00227)	7	8.80%	6.90%
receptor (PC00197)	7	8.80%	6.90%
extracellular matrix protein (PC00102)	6	7.50%	5.90%
oxidoreductase (PC00176)	4	5.00%	4.00%
cell adhesion molecule (PC00069)	3	3.80%	3.00%
calcium-binding protein (PC00060)	3	3.80%	3.00%
cytoskeletal protein (PC00085)	2	2.50%	2.00%
isomerase (PC00135)	1	1.30%	1.00%
structural protein (PC00211)	1	1.30%	1.00%

Ball Milled

Category Name (Accession)	# genes	% genes hit against total genes	% gene hit against total # protein class hits
cytoskeletal protein (PC00085)	17	16.80%	14.20%
defense/immunity protein (PC00090)	16	15.80%	13.30%
enzyme modulator (PC00095)	15	14.90%	12.50%

structural protein (PC00211)	13	12.90%	10.80%
transfer/carrier protein (PC00219)	12	11.90%	10.00%
hydrolase (PC00121)	11	10.90%	9.20%
signaling molecule (PC00207)	11	10.90%	9.20%
oxidoreductase (PC00176)	7	6.90%	5.80%
transporter (PC00227)	5	5.00%	4.20%
receptor (PC00197)	4	4.00%	3.30%
extracellular matrix protein (PC00102)	4	4.00%	3.30%
cell adhesion molecule (PC00069)	2	2.00%	1.70%
transcription factor (PC00218)	1	1.00%	0.80%
nucleic acid binding (PC00171)	1	1.00%	0.80%
calcium-binding protein (PC00060)	1	1.00%	0.80%

Supplemental Data Table 10. Differences in pathways and protein characteristics due to incubation in either normal or hyperlipidemic serum

All proteins found to associate with SWCNTs following incubation in either normal or hyperlipidemic serum were individually uploaded into genontology.org's enrichment analysis tool and serached for biological processes and Mus musculus. This search provided lists of PANTHER pathways and protein characteristics. Analysis utilized the Bonferroni correction for multiple testing with a statistical significance established at p<0.05. The table presents all pathways that were identified regardless of signifiicance in order to demonstrate differences between groups. All mapped IDs were utilized for PANTHER analysis of pathways and protein characteristics. Assessment of PANTHER pathways allows for the identification of distinct pathways that may be disrupted by SWCNTs in differing environments. Evaluation of protein characteristics allows for the understanding of protein properties in these normal or hyperlipidemic envrionments that may result in differential association with SWCNTs.

Biological pathways related to the BC that formed following incubation normal or hyperlipidemic serum

Normal BC

PANTHER Pathways	# genes	Fold Enrichment	P-value
Blood coagulation (P00011)	14	51.49	7.89E-18
Plasminogen activating cascade (P00050)	5	55.17	6.88E-06
Integrin signalling pathway (P00034)	3	2.95	1.00E+00
Inflammation mediated by chemokine and cytokine signaling pathway (P00031)	3	2.16	1.00E+00
Wnt signaling pathway (P00057)	2	1.23	1.00E+00
Vitamin D metabolism and pathway (P04396)	2	28.86	3.60E-01
Nicotinic acetylcholine receptor signaling pathway (P00044)	2	3.95	1.00E+00
Huntington disease (P00029)	2	2.59	1.00E+00
Cytoskeletal regulation by Rho GTPase (P00016)	2	4.75	1.00E+00
Cadherin signaling pathway (P00012)	2	2.44	1.00E+00
Alzheimer disease-presenilin pathway (P00004)	2	3.05	1.00E+00
CCKR signaling map (P06959)	1	1.14	1.00E+00
p53 pathway (P00059)	1	2.23	1.00E+00
T cell activation (P00053)	1	2.04	1.00E+00
FAS signaling pathway (P00020)	1	5.86	1.00E+00
B cell activation (P00010)	1	2.64	1.00E+00

Lipid BC

PANTHER Pathways	# genes	Fold Enrichment	P-value
Blood coagulation (P00011)	15	52.95	2.41E-19
Plasminogen activating cascade (P00050)	5	52.95	8.45E-06
Integrin signalling pathway (P00034)	3	2.83	1.00E+00
Inflammation mediated by chemokine and cytokine signaling pathway (P00031)	3	2.08	1.00E+00
Huntington disease (P00029)	3	3.72	1.00E+00
Vitamin D metabolism and pathway (P04396)	2	27.69	3.90E-01
Nicotinic acetylcholine receptor signaling pathway (P00044)	2	3.79	1.00E+00
Cytoskeletal regulation by Rho GTPase (P00016)	2	4.56	1.00E+00
Cadherin signaling pathway (P00012)	2	2.34	1.00E+00
Alzheimer disease-presenilin pathway (P00004)	2	2.93	1.00E+00
CCKR signaling map (P06959)	1	1.09	1.00E+00
p53 pathway (P00059)	1	2.14	1.00E+00
Wnt signaling pathway (P00057)	1	0.59	1.00E+00
T cell activation (P00053)	1	1.96	1.00E+00
Glycolysis (P00024)	1	6.43	1.00E+00
FAS signaling pathway (P00020)	1	5.63	1.00E+00
B cell activation (P00010)	1	2.54	1.00E+00

Characteristics of proteins found to associate with the BC that formed following incubation in normal or hyperlipidemic serum

Normal BC

Category Name (Accession)	# genes	% genes hit against total genes	% gene hit against total # protein class hits
enzyme modulator (PC00095)	24	20.20%	15.60%
signaling molecule (PC00207)	19	16.00%	12.30%
hydrolase (PC00121)	18	15.10%	11.70%

defense/immunity protein (PC00090)	17	14.30%	11.00%
transfer/carrier protein (PC00219)	16	13.40%	10.40%
cytoskeletal protein (PC00085)	12	10.10%	7.80%
receptor (PC00197)	10	8.40%	6.50%
transporter (PC00227)	8	6.70%	5.20%
structural protein (PC00211)	8	6.70%	5.20%
extracellular matrix protein (PC00102)	8	6.70%	5.20%
calcium-binding protein (PC00060)	5	4.20%	3.20%
oxidoreductase (PC00176)	4	3.40%	2.60%
cell adhesion molecule (PC00069)	4	3.40%	2.60%
isomerase (PC00135)	1	0.80%	0.60%

Lipid BC

Category Name (Accession)	# genes	% genes hit against total genes	% gene hit against total # protein class hits
enzyme modulator (PC00095)	21	16.90%	13.00%
transfer/carrier protein (PC00219)	17	13.70%	10.60%
defense/immunity protein (PC00090)	17	13.70%	10.60%
cytoskeletal protein (PC00085)	17	13.70%	10.60%
signaling molecule (PC00207)	17	13.70%	10.60%
hydrolase (PC00121)	16	12.90%	9.90%
structural protein (PC00211)	13	10.50%	8.10%
receptor (PC00197)	9	7.30%	5.60%
oxidoreductase (PC00176)	8	6.50%	5.00%
transporter (PC00227)	7	5.60%	4.30%
extracellular matrix protein (PC00102)	6	4.80%	3.70%
calcium-binding protein (PC00060)	5	4.00%	3.10%
cell adhesion molecule (PC00069)	4	3.20%	2.50%
isomerase (PC00135)	2	1.60%	1.20%
transcription factor (PC00218)	1	0.80%	0.60%
nucleic acid binding (PC00171)	1	0.80%	0.60%
